# Multiplexed mobilization and expression of biosynthetic gene clusters

**DOI:** 10.1038/s41467-022-32858-0

**Published:** 2022-09-06

**Authors:** Vincent Libis, Logan W. MacIntyre, Rabia Mehmood, Liliana Guerrero, Melinda A. Ternei, Niv Antonovsky, Ján Burian, Zongqiang Wang, Sean F. Brady

**Affiliations:** Laboratory of Genetically Encoded Small Molecules, The Rockefeller University, 1230 York Avenue, New York, NY 10065 USA

**Keywords:** Antibiotics, Applied microbiology, Metabolic engineering

## Abstract

Bacterial genomes contain large reservoirs of biosynthetic gene clusters (BGCs) that are predicted to encode unexplored natural products. Heterologous expression of previously unstudied BGCs should facilitate the discovery of additional therapeutically relevant bioactive molecules from bacterial culture collections, but the large-scale manipulation of BGCs remains cumbersome. Here, we describe a method to parallelize the identification, mobilization and heterologous expression of BGCs. Our solution simultaneously captures large numbers of BGCs by cloning the genomes of a strain collection in a large-insert library and uses the CONKAT-seq (co-occurrence network analysis of targeted sequences) sequencing pipeline to efficiently localize clones carrying intact BGCs which represent candidates for heterologous expression. Our discovery of several natural products, including an antibiotic that is active against multi-drug resistant *Staphylococcus aureus*, demonstrates the potential of leveraging economies of scale with this approach to systematically interrogate cryptic BGCs contained in strain collections.

## Abstract (web summary)

Efficient mobilization and expression of biosynthetic gene clusters (BGCs) into heterologous hosts is needed for microbial natural products discovery. Here, the authors improve the CONKAT-seq strategy by simultaneously capturing the BCGs into a single large insert library and demonstrate its ability to discover natural products with new structures and potent antibacterial activity.

## Introduction

Microbial natural products have been a uniquely generous source of life-saving drugs, unfortunately discovery rates are no longer keeping pace with unresolved medical needs, especially in the face of the growing antibiotic resistance crisis^1^. Bioinformatic analysis of sequenced microbial genomes has uncovered a vast reservoir of uncharacterized biosynthetic gene clusters (BGCs) that are predicted to encode unexplored metabolites. Accessing natural products from these cryptic BGCs by culturing wild strains is challenging as most BGCs are not readily expressed under laboratory conditions, and when they are expressed, their products can be masked by complex natural metabolite mixtures. Together with these issues, the pervasive transfer of BGCs across species boundaries has burdened strain-centric approaches with diminishing returns due to high rediscovery rates. Emerging BGC-centric discovery methods circumvent the diminishing returns of strain-based efforts by dereplicating BGCs at an earlier stage through bioinformatics and facilitate natural product discovery through mobilization of cryptic BGCs into heterologous hosts where they can be interrogated in well-defined genetic and metabolic backgrounds. Despite these advantages, examining large numbers of BGCs with existing methods remains cumbersome, which has restricted BGC-centric discovery approaches to relatively small-scale, carefully curated BGC collections^2^.

Here, we describe an efficient strategy to capture, prioritize, and mobilize the uncharacterized BGCs present in a strain collection, regardless of their size. Our solution leverages CONKAT-seq (co-occurrence network analysis of targeted sequences), a strategy originally developed to interrogate BGCs present in metagenomes^3^, and improves it to generate economies of scale when mobilizing BGCs originating from strain collections. This easily scalable approach enabled us to interrogate 70 large nonribosomal peptide synthetase (NRPS) and polyketide synthase (PKS) BGCs in multiple heterologous expression hosts. We detect the production of differential chemical entities by 24% of the cryptic BGCs and illustrate the potential of this approach by characterizing three natural products, including an antibiotic.

## Results

### Capture and detection of large numbers of BGCs in parallel

Three main strategies are commonly used to study a BGC in a heterologous host. These include targeted methods that involve genomic sequencing followed by either DNA synthesis^4^ or direct cloning^5,6^, as well as untargeted methods that involve constructing a random genomic library followed by recovery of a clone of interest^7^. We reasoned that random cloning is the most compatible with multiplexing and could provide significant economies of scale if the number of BGCs cloned in parallel became high enough. The efficiency of accessing a BGC using a genomic library is limited by the cumbersome nature of cloning high molecular weight DNA, as well as the tedious screening required to localize a clone carrying a full BGC. Our solution deals with these bottlenecks by first simultaneously extracting and cloning multiple genomes into a single large-insert library. This dramatically reduces the effort required to clone individual genomes but results in a complex multi-genomic library. We overcome this complexity with CONKAT-seq^3^, a targeted sequencing workflow that uses library-wide co-occurrence analysis to detect, localize and evaluate thousands of BGCs simultaneously. Together these two steps transform the cumbersome process of individually cloning a large number of BGCs into a sequencing problem, which is efficiently solved in a multiplexed fashion using CONKAT-seq.

**Fig. 1:**
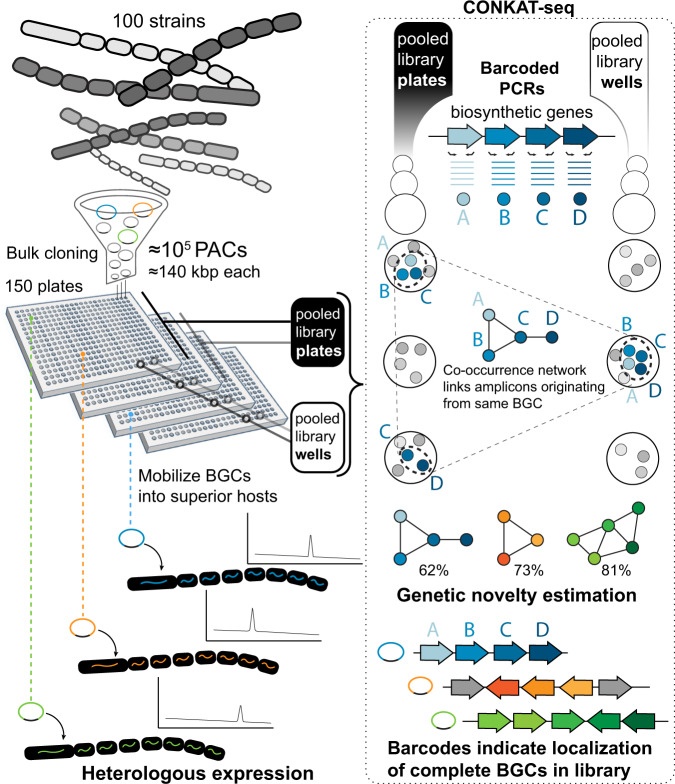
General strategy for multiplexed capture, identification, and mobilization of biosynthetic gene clusters (BGCs). A strain collection is cloned at random in the form of a large-insert genomic library (PACs P1-derived Artificial Chromosomes). CONKAT-seq (co-occurrence network analysis of targeted sequences) is used to detect captured BGCs, evaluate their novelty and determine their physical location in the arrayed library. Finally, cryptic BGCs are mobilized into heterologous hosts to access their products.

To test this two-step approach, we selected 100 *Streptomyces* strains, most of which lacked any associated genome sequence data (Supplementary Table 1), and pooled comparable amounts of their mycelia. A large-insert clone library was generated with DNA isolated from this pool using a PAC shuttle vector that replicates in *Escherichia coli* and contains all elements required for transfer and integration into *Streptomyces* hosts. The resulting library consisted of ~60,000 *E. coli*-based clones with an average insert size of ~140 kb and was stored as individual clones in separate wells across 150 microplates. To localize cloned BGCs to specific library wells using CONKAT-seq, barcoded degenerate primer pairs are used to specifically sequence biosynthetic genes with regions sufficiently conserved to be amplified. While any combination of primers targeting co-occurring biosynthetic genes can be used in such an analysis^3,8–10^, we focused our discovery efforts on NRPS and PKS biosynthesis because their repetitive modular architecture allows multiple domains of the same BGC to be amplified using a single set of primers (i.e., targeting adenylation and ketosynthase domains, respectively). In addition to this facilitated amplification, we chose to examine NRPS and PKS BGCs because they represent very large pools of unstudied biosynthetic diversity and are the source of the largest share of microbe-derived drugs^11^. The number of PCR reactions required to track cloned BGCs was reduced by compressing library aliquots into two types of pools: plate-pools contained clones found on the same plate, and well-pools contained clones found on different plates but in the same well position (Fig. 1). CONKAT-seq uses amplicon barcode information to identify the domain sequences in each pool, and with this data triangulates the good positions of each domain in the original library. As domains that belong to the same BGC are often captured on the same clones, they exhibit co-occurrence patterns that can be detected statistically (Fisher’s exact test) and used to create BGC-specific domain networks.

From library-wide adenylation and ketosynthase domain amplicon data, CONKAT-seq predicted 317 distinct domain networks. Degenerate primer and cloning biases can affect the fraction of BGCs in the source strain collection that is identified using this method. To estimate the success rate of our approach at recovering NPRS and PKS BGCs contained in the source collection, we compared the networks detected by CONKAT-seq to the BGCs present in 15 of the source strains for which complete genome sequences could be found in publicly available databases (Supplementary Table 16). In this analysis, we found that 72% of the NPRS and PKS BGCs present in these 15 genomes had been cloned in the library and detected by CONKAT-seq. Additional primer degeneracy, sequencing, or further expansion of the multi-genomic library would undoubtedly increase this number; however, attempts to examine all BGCs in a strain collection will likely lead to diminishing returns using any method. CONKAT-seq provides a unique means of maximizing the number of cryptic BGCs that can be interrogated from a culture collection with limited resources rather than exhaustively recovering BGCs from individual genomes.

### Prioritization of BGCs and interrogation by heterologous expression

**Fig. 2:**
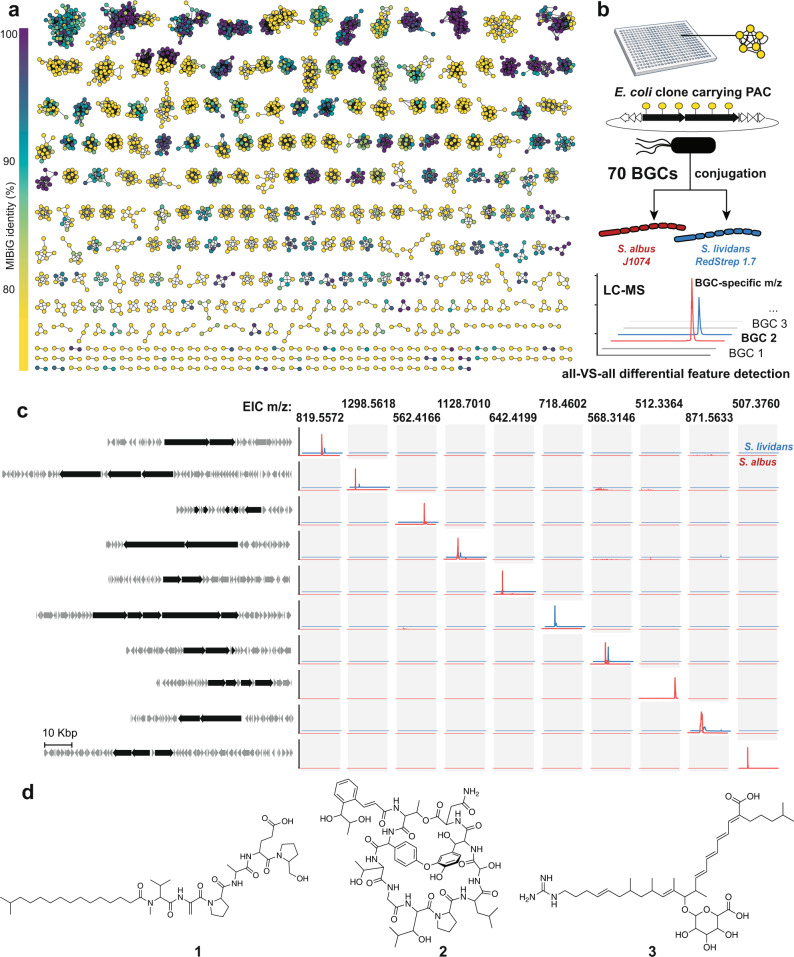
Experimental performance of a streamlined genes-to-molecules discovery pipeline. **a** Amplicons derived from biosynthetic genes found in close proximity on chromosomes are connected in the form of networks using CONKAT-seq. Networks may correspond to a single BGC or in some cases multiple BGCs found close to each other on the original chromosome. Each node in a network corresponds to an amplified biosynthetic domain and is colored based on its amino acid identity to the closest known BGC for which a metabolite has been reported. **b** Individual BGCs are transferred into hosts and cultures are interrogated by LC-HRMS. Untargeted metabolomics is used to identify BGC-specific mass features through an all-versus-all comparison. **c** Examples of extracted ion chromatograms (EIC) of BGC-specific masses associated with cryptic BGCs. BGCs are depicted on the left with their core PKS and NRPS genes colored in black. In each row, the signal associated with a BGC is depicted in blue when in *S. lividans* and red when in *S. albus*. **d** Metabolites identified in this study. Source data are provided as a Source Data file.

We evaluated the biosynthetic novelty of each network by comparing its sequences to known BGCs. A network was considered to arise from a BGC lacking a reported product if more than two-thirds of its domains displayed less than 80% amino acid identity to proteins in the MIBiG database^3^. According to this classification, 59% of the detected networks are associated with uncharacterized BGCs (Fig. 2a). Seventy BGCs associated with networks of various predicted biosynthetic novelty were recovered by selecting from the library array PAC clones that were expected to contain full BGCs (i.e., harbor all domains in a CONKAT-seq network). This corresponded to 50 networks classified as uncharacterized, 12 control networks harboring domains matching a known BGC at >90% amino acid identity, and 8 networks with intermediate similarity. Full sequencing and analysis with the ClusterBlast algorithm^12^ confirmed the novelty of the BGCs associated with the 50 low-similarity networks as well as the novelty of 7 of 8 intermediate networks. All 12 of the control networks closely matched a characterized BGC.

Cloned BGCs were transferred into two *Streptomyces* strains (*S. albus* J1074 and *S. lividans* RedStrep 1.7) with reported superior abilities as heterologous expression hosts (Fig. 2b)^13,14^. Following fermentation, cultures were extracted and analyzed by liquid chromatography-mass spectrometry. The identification of BGC-specific features was straightforward as the chemical composition of a culture harboring a particular BGC could be compared to all other strains harboring unrelated BGCs in order to detect unique chemical features. Ten known BGCs and 15 uncharacterized BGCs produced a unique differential mass spectral feature in at least one host (Fig. 2c and Supplementary Fig. 1). The exact mass for the expected product, or a reported congener, was detected in experiments with 8 out of 10 known BGCs, suggesting that the detection of a differential mass spectral feature is likely to indicate production of a natural BGC product in our heterologous expression setup. One uncharacterized BGC produced a mass matching an orphan natural product (i.e., no known BGC), lydiamycin, whose structure we confirmed (Supplementary Fig. 2). BGC activation only partially overlapped between the two hosts (14 unique to *S. albus*, two unique to *S. lividans*, nine in both, Supplementary Table 14), highlighting the advantage of being able to easily mobilize cloned BGCs to multiple genetic backgrounds to improve activation rates. We observed a higher rate of successful expression with BGCs associated with known molecules (69%) than with uncharacterized BGCs (24%), suggesting that known products may have been discovered early on because their BGCs are more prone to be expressed under laboratory conditions.

### Discovery of natural products

We selected three uncharacterized BGCs to investigate further and isolated their respective products, which we named prolinolexin (**1**), cinnamexin (**2**), and conkatamycin (**3**) (Supplementary Figs. 3–34, Supplementary Tables 2–9). Each natural product represents an uncharacterized structural family (Supplementary Fig. 35), suggesting we are, in fact, accessing previously underexplored biosynthetic diversity. Conkatamycin is particularly interesting as its biosynthesis is predicted to use a rare PKS building block (isohexylmalonyl-CoA) reported in only a small number of known natural products, and it showed potent antibacterial activity against all multi-drug resistant *Staphylococcus aureus* strains we tested (see Supplementary Tables 10 and 11). In addition to appearing unaffected by resistance to known antibiotics, we were unable to raise *S. aureus * mutants which were resistant to conkatamycin (≥4× the minimum inhibitory concentration (MIC), see Methods). Conkatamycin does not appear to lyse or depolarize the bacterial membrane nor inhibit cell wall biosynthesis (Supplementary Table 12, Supplementary Fig. 36). Finally, we observed that the MIC of conkatamycin is dependent on the pH of the media (Supplementary Table 13), similar to the ECO-0501 antibiotic, the closest known structure to conkatamycin^15^, whose true mechanism of action was never reported. While conkatamycin’s mechanism of action remains elusive, we believe that the discovery of a molecule that circumvents resistance to many commonly used antibiotics suggests an uncommon mechanism of action and is generally promising for the potential of this approach to uncover biomedically interesting natural products.

## Discussion

Compared to existing methods (see Supplementary Table 15), the BGC screening strategy outlined here replaces the current bottleneck of individually cloning BGCs with an easily scalable process that leverages next-generation sequencing. Consequently, the efficiency of this approach will only further increase over time as sequencing capabilities continue to improve. The approach does not require the ad hoc design of specific reagents for each BGC that is examined, and it does not require knowing the sequences of the target strains or their BGCs beforehand. While cloning bias and the use of degenerate primers may limit its ability to exhaustively interrogate BGCs contained in a strain collection, its efficient use of parallelization provides economies of scale that are not seen with other methods (see supplementary Note 1, Supplementary Table 17). As illustrated here with NRPS and PKS, arguably the hardest classes of BGCs to mobilize due to their size and repetitive sequences, we believe that the large-scale application of this method should enable the streamlined interrogation of cryptic BGCs found in culture collections.

## Methods

### PAC library cloning

Spore stocks of 100 Streptomyces species were grown individually in TSB medium, and mycelia were harvested just after reaching stationary phase. Mycelia were centrifuged, and 40 µL of each pellet was pooled together. The pooled mycelia were washed twice in 2 volumes of buffer (200 mM NaCl; 10 mM TrisHCl pH8; 100 mM EDTA), centrifuged, and flash frozen in liquid nitrogen. PAC library construction was performed by Bio S&T (Montreal, Canada) using the pESAC-13-Apramycin vector and maintained in *E. coli* DH10B. The average insert size of 140 kb was determined by pulse field gel electrophoresis of 18 randomly selected clones digested with DraI.

### Amplicons sequencing

Library plates were replicated using a 384 pin transfer tool, and the content of each plate was pooled to form 150 plate-pools. Batches of 25 library plates had 2 µL aliquots of each well taken and pooled on top of another in a single 384-well plate using a Viaflo (Integra) liquid handling platform. A total of 2304 well-pools were constituted in the form of six 384-well plates. PCR amplification reactions were setup with live cells as templates for these six plates, as well as for a 384-well plate containing the 150 plate-pools and the 100 *Streptomyces spp* genomic extractions. PCR reactions were setup in 384-well PCR plates using barcoded degenerate primer pairs targeting adenylation and ketosynthase domains^3^. Each reaction contained 6 μL of FailSafe Buffer G ×2 (Epicentre), 4.3 μL of water, 0.5 μL of each column/row barcoded primer (100 μM), 0.2 μL of rTaq polymerase (Bulldog Bio), and 0.5 μL of cells from a specific pool. PCR cycle conditions were set to: 95 °C 5 min, (95 °C 30 s, 56.3 °C 30 s, 72 °C 45 s) × 35 cycles, 72 °C 5 min. To permit parallel sequencing of amplicons from multiple library sub-pools, we used a set composed of 24 barcoded forward/columns primers and 16 barcoded reverse/rows primers forming 384 unique primer pairs. Primers were composed of (i) an invariant landing pad for Illumina p5 or p7 sequence, (ii) a unique golay barcode (12 bp) that identifies a column (forward primer) or a row (reverse primer), (iii) a spacer sequence required to phase amplicon sequence and increase bases diversity, and (iv) a degenerate sequence targeting adenylation (Fw: 5’-SATBTAYACSTCVGGHWCSAC Rvs: 5’-CCANRTCNCCBGTSYKGTACA) or ketosynthase domains (Fw: 5′-GTNCCSGTSCCRTGBGCYTCS Rvs: 5′-TGYTCSDSSTCGCTSGTSGCS). PCR products were pooled as collections of 384 reactions from each plate, size-selected according to expected amplicon length, and used as a template for a second round of PCR to append plate-specific sample indexes and sequencing adapters. The second PCR was set using 10 μL of FailSafe Buffer G ×2 (Epicentre), 3.8 μL of water, 0.5 μL of Illumina universal forward (100 μM), 0.5 μL of indexed reverse primers (100 μM), 0.2 μL of Q5 polymerase and 5 μL of purified amplicon product from the first round PCR (100 ng). PCR cycle conditions were set to: 98 °C for 1 min, (98 °C for 30 s, 62 °C for 30 s, and 72 °C for 45 s) × 6 cycles, and finally, 72 °C for 5 min. Second PCR products were size-selected, gel extracted and quantified with a Qubit fluorometer (Thermo Fisher Scientific). Purified second PCR products were mixed in an equal molar ratio to a final concentration of 4 nM, and the resulting library was sequenced on a MiSeq instrument (MS-103-1001, Illumina) according to standard amplicon sequencing workflow with 10% phiX.

### Predicting networks of biosynthetic domains and their physical location in the library

Following amplicon sequencing, a raw fastq file was obtained for each 384 PCR plate and for each type of domain (adenylation and ketosynthase). Reads were demultiplexed to single pool files based on the primer encoded barcodes using a custom Python script [https://github.com/brady-lab-rockefeller/paired-end-debarcoder]. Forward reads (R1) were trimmed using VSEARCH16^16^ (version 2.9.1) “-fastx_truncate” option with parameters “-stripleft 21 -trunclen 200” to remove primer sequence and set a fixed read length. Trimmed reads were de-replicated using VSEARCH “-fastx_uniques” option with “-size_out” flag for propagating de-replicated read counts. De-replicated reads from all library sub-pools were then sorted by a number of reads and clustered (95% sequence similarity cut-off) using VSEARCH “--cluster_size” option with “--id 0.95 --iddef 1 --sizein --sizeout --centroids --uc” parameters. The output clustering table was filtered by removing sequences with read count smaller than 3. For each cluster, we also removed sequences with low read counts (<5%) relative to the maximum read count of a sequence in the cluster, this was done independently for reads acquired in different MiSeq runs (well-pools / plate-pools / genomes) as the maximum read count observed for each cluster varied in each run. The amplicon clustering tables for adenylation and ketosynthase domains were merged prior to joint analysis. The merged amplicon clustering table contains the list of domain variants (95% sequence identity clusters) and specifies the set of pools in which each domain was detected in. To identify biosynthetic domains that originate from neighboring regions of genomic DNA, CONKAT-seq uses domain pool occurrences to construct the 2 × 2 contingency table for pairs of domain variants (specifying the number of pools in which both domain variants, one of the two only, or none of them were identified). Non-random co-occurrence of domain variants (i.e., domain pairs observed together more often than expected by chance under the null model of random, non-linked, and dispersal of domains across pools) is statistically tested using one-sided Fisher’s exact test as implemented in the “fisher_exact” function in “scipy.stats” module. This test was conducted independently for the 2304 well-pools, 150 plate-pools, and 100 genomes as the probability of random co-occurrence were different in each of the three cases. For each pair of domains, *p*-values lower than 1 obtained from each test were multiplied together. Pairs of domains with a *p*-value < 10^−5^ overall and odds >30 in the well-pools were considered to be physically linked. Based on a pairwise list of statistically significant links, we constructed a graph representation of domain networks where nodes represent biosynthetic domains and edges link domains that are predicted to be physically clustered on the genome. In some cases, we observed that sequencing errors or the presence of closely related BGCs in the library can lead to the presence of very similar sequences within the same network of domains. We also note that biases in the sampling of domain variants are expected to arise due to differential amplification efficiencies and variability in sequencing depth between pools. Such biases can contribute to a failure to detect co-occurring domains (false negatives) but are less likely to generate false-positive associations. While our empirical testing of CONKAT-seq results verified the vast majority of domain clustering predictions, we note that systematic errors during library preparation or in the assignment of amplicon reads to their pools of origin (for example, due to physical cross-contamination of library pools or index-switching of barcoded reads) could result in the false clustering of unrelated domain pairs. In addition, because PAC inserts can span >150 kb, the domains of neighboring—but unrelated—BGCs can sometimes be merged in a single network. To mitigate this effect, a final step iteratively analyzes the topology of each network and eliminates a node or an edge that is the sole connection between several otherwise unrelated communities of domains. This is done by calculating the betweenness centrality of each node or edge of a network and testing in order of decreasing centrality if their removal is sufficient to create multiple large components in the graph. Finally, in parallel to predicting networks of domains, CONKAT-seq can triangulate the location of domains to a single clone in the original clone library if a domain appears only once in a plate of 384 well-pools made of 25 library plates and only once in the corresponding 25 plate-pools. The clones harboring the complete BGC are most likely to be recovered from the locations where each domain of the corresponding network is predicted to be localized. Analysis script and graphML output are available online (https://github.com/VincentLibis/multiplexed_BGC_recovery).

### Full sequencing of BGCs

The selected library well was grown overnight in LB with apramycin selection and pooled together. PACs were extracted in batches using miniprep buffers to lyze the cells and neutralize the lysate (QIAprep, Qiagen), followed by ethanol precipitation. Eight hundred nanograms of purified PAC DNA comprising 30–60 different PACs were processed with a Rapid Sequencing Kit (SQK-RAD004) and sequenced with a MinION flowcell R10.3 on a MinION Mk1B (Oxford Nanopore). Raw reads were processed with minimap2^17^, and samtools^18^, following https://github.com/VincentLibis/multiplexed_BGC_recovery/blob/main/notebooks/nanopore_processing_PACs.ipynb and assembled with Flye^19^. The resulting contigs were assigned to their corresponding CONKAT-seq network by mapping at least two of the sequences composing the network on the contigs with a pairwise identity >93%. Sequences were further polished with short reads as follows. Sequencing libraries were prepared using a Nextera XT DNA Sample Preparation Kit (FC-131-1024) with Nextera XT Index kit (FC-131-1001) based on protocols provided by the manufacturer (Illumina) in batches of 10 PACs per index. The quality of the final library pool was verified using HS D1000 ScreenTape (TapeStation 2200, Agilent Technologies) prior to being sequenced using MiSeq Reagent Nano Kit v2 on a MiSeq sequencer (Illumina). For each PAC, short reads were mapped onto the corresponding long-read contig, and the resulting polished consensus sequence was analyzed using antiSMASH 6^12^. Sequences of the BGCs encoding Lydiamycin A, Prolinolexin (**1**), Cinnamexin (**2**), and Conkatamycin (**3**) are available from Genbank (Accession numbers OL452059 - OL452062), and all other sequences and analysis results are available at GitHub [https://github.com/VincentLibis/multiplexed_BGC_recovery/tree/main/BGCs].

### Conjugation and fermentation

*E. coli* DH10B clones harboring PACs of interest were electroporated with the pUZ8002 plasmid, and transformants were selected with apramycin and kanamycin. DH10B (PAC, pUZ8002) were grown until OD 0.4 in 50 mL of LB supplemented with antibiotics and conjugated with acceptor strains *S. albus* J1074 and *S. lividans* RedStrep 1.7 following the standard protocol. Three exconjugants were randomly selected for each PAC, and starter cultures were grown for 48 h in tryptic soy broth (2 mL, 30 °C, 220 rpm). 500 µL of starter culture was used to inoculate 50 mL of R5a production medium containing: 100 g/L sucrose, 10 g/L d-glucose, 5 g/L yeast extract, 10.12 g/L MgCl_2_·6H_2_O, 0.25 g/L K_2_SO_4_, 0.1 g/L casamino acids, 21 g/L MOPS, 2 g/L NaOH, 5.88 mg/L CaCl_2_, 80 μg/L ZnCl_2_, 400 μg/L FeCl_3_·6H_2_O, 20 μg/L MnCl_2_, 20 μg/L CuCl_2_, 20 μg/L Na_2_B_4_O_7_·10H_2_O, 20 μg/L (NH_4_)6Mo_7_O_24_·4H_2_O, pH = 6.85. Cultures were fermented in 125 mL baffled flasks (30 °C, 220 rpm) for 14 days.

### General analytical procedures

All solvents used for chromatography were HPLC grade or higher. For all liquid chromatography (unless noted), solvent A = H_2_O (0.1% v/v formic acid) and solvent B = CH_3_CN (0.1% v/v formic acid). UPLC-LRMS was performed on a Waters Acquity system equipped with QDa and PDA detectors, a Phenomenex Synergi Fusion-RP 80 Å column (2.0 × 50 mm, 4 μm) and controlled by Waters MassLynx software. The following chromatographic conditions were used for UPLC-LRMS: 5% B from 0.0 to 0.9 min, 5% to 95% B from 0.9 to 4.5 min, 95% B from 4.5 to 5.0 min (flow rate of 0.6 mL/min and 10 μL injection volume). UPLC-HRMS data was acquired on a SCIEX ExionLC UPLC coupled to an X500R QTOF mass spectrometer, equipped with a Phenomonex Kinetex PS C18 100 Å column (2.1 × 50 mm, 2.6 μm) and controlled by SCIEXOS software. The following chromatographic conditions were used for UPLC-HRMS: 5% B from 0.0 to 1.0 min, 5% to 95% B from 1.0 to 10.0 min, 95% B from 10.0 to 12.5 min, 95% to 5% B from 12.5 to 13.5 min, and 5% B from 13.5 to 17.0 min (flow rate of 0.4 mL/min and 5 μL injection volume). The following ESI + HRMS conditions were used: temperature of 500 °C, spray voltage of 5500 V. Automated flash column chromatography was performed using a CombiFlash Rf200 system (Teledyne ISCO). Semipreparative HPLC was performed on an Agilent 1200 Series HPLC with UV detection and equipped with an XBridge Prep C18 130 Å column (10 × 150 mm, 5 μm). All NMR spectra of **1**–**3**, unless noted, were acquired on a Bruker Avance DMX 600 MHz spectrometer equipped with a cryogenic probe (The Rockefeller University, New York, NY). All spectra were recorded at room temperature unless noted otherwise. HMBC NMR spectra of **2** in CD_3_OD were acquired on a Bruker Avance III HD 800 MHz spectrometer equipped with a 5 mm TCI cryogenic (CUNY Advanced Science Research Center Biomolecular NMR Facility, New York, NY). ^1^H NMR spectra of **1** below 25 °C were acquired on a Bruker Avance III 500 MHz spectrometer equipped with a 5 mm cryogenic probe (Analytical NMR Core Facility, Memorial Sloan Kettering Cancer Center, New York, NY). Chemical shift values are reported in ppm and referenced to residual solvent signals: CD_3_OD (^1^H: 3.31 ppm and ^13^C: 49.00 ppm), DMSO-*d*_*6*_ (^1^H: 2.50 ppm and ^13^C: 39.52 ppm), and acetone-*d*_*6*_ (^1^H: 2.05 ppm and ^13^C: 29.84 ppm). GC-qTOF data was acquired on an Agilent system equipped with a 5975 mass selective detector and an Agilent DB_5_-MS 30 m (0.25 mm, 0.25 µm) column (Cancer Metabolism Center, Memorial Sloan Kettering Cancer Center, New York, NY).

### Extraction and untargeted metabolomics analysis

Ten milliliters of each culture was extracted with an equal volume of ethyl acetate. The organic phase (10 mL) was dried under vacuum and resuspended in 500 µL of methanol. Five microliters were analyzed by UPLC/HRMS in positive ionization mode. Raw data was processed using MZmine 2.53^20^. Mass detection was performed using the centroid detector with a noise level value of 3000 counts/s. Chromatograms were built using the ADAP module with the following parameters: Min group size in # of scans = 5; Group intensity threshold = 300; Min highest intensity = 3000; *m/z* tolerance = 0.01 *m/z* or 10 ppm. Chromatogram deconvolution was performed using the local minimum search algorithm with the following parameters: Chromatographic threshold = 85%; Search minimum in RT range = 0.1 min; Minimum relative height = 1%; Minimum absolute height = 5000; Minimum ratio of peak top/edge = 1.3. Deistopting was performed with the following parameters: *m/z* tolerance = 0.01 *m/z* or 10 ppm; Retention time tolerance = 0.5; Maximum charge = 1. Chromatograms were aligned with the Join aligner with the following parameters: *m/z* tolerance = 0.01 or 10 ppm; Weight for *m/z* = 20; Retention time tolerance = 0.5 min; Weight for RT = 10. Gaps in the alignment were filled with Peak finder with the following parameters: Intensity tolerance = 0.05%; *m/z* tolerance = 0.01 *m/z* or 10 ppm; Retention time tolerance = 0.5 min. A gap-filled matrix of the peak area for each mass feature (columns) and cultures (rows) was used as a heatmap to look for unique mass features appearing specifically in all three culture replicates of a given BGC.

### Isolation

Spores of strains carrying BGCs of interest were used to seed starter cultures in tryptic soy broth, which were grown for 48 h (50 mL, 30 °C, 220 rpm). Three milliliters of starter culture was transferred into 2.8 L baffled Fernbach flasks containing 700 mL of R5a production medium and shaken at 200 rpm for 14 days at 30 °C. In the case of Conkatamycin, the flasks also contained 20 g of autoclaved HP20 resin. 10 Fernbach flasks (7 L of medium) for each strain were used to isolate each molecule. For Prolinolexin (**1**) and Cinnamexin (**2**) cultures were extracted with ethyl acetate 1:1. The dried extract was adsorbed onto C18 reversed-phase silica gel and was initially partitioned by medium-pressure liquid chromatography (50 g Gold HP C18 column, 10% B from 0.0 to 1.0 min, 10% to 95% B from 1.0 to 18.0 min, 95% B from 18.0 to 22.0 min, 40 mL/min). No formic acid was present in the solvents in the case of Cinnamexin (**2**), which appeared prone to degradation in acidic conditions. For Conkatamycin (**3**), the HP20 resin was collected from the cultures using cheesecloth and dried. The dried resin was packed into a column, washed with 2 L of H2O, and eluted with 2 L of methanol. The methanolic elution was concentrated in vacuo at 30 °C and adsorbed onto silica gel. The crude extract was initially partitioned by medium-pressure liquid chromatography (RediSep Rf Silica Gel Disposable Flash column, 12 g, gradient elution from 100% to 0% Hexanes/Ethyl acetate for 20 min, followed by 100% Chloroform isocratic elution for 10 min). For all three molecules, fractions were analyzed by UPLC-DAD-MS, and those containing the targeted masses were combined. Further separation was carried out by semipreparative HPLC to afford the pure form of each compound.

### Microbial susceptibility assay

MIC assays were conducted following the protocol recommended by the Clinical and Laboratory Standards Institute^21^. Briefly, assays were performed in duplicate in 96-well microliter plates (*n* = 2). A single colony of each assayed bacterial strain was inoculated into 5 mL of LB broth medium and grown overnight at 37 °C. The saturated overnight culture was then diluted 5000-fold in fresh LB, and transferred into the assay plates. Conkatamycin was dissolved in methanol to give a 1.6 mg/mL stock solution. The stock solution was diluted across 96-well plates using a two-fold serial dilution to give a concentration range of 16–0.125 µg/mL with a final volume of 100 µL in each well. The top and bottom rows of each plate were filled with 100 µL of LB broth without compound to avoid edge effects. The last well in each row contained bacteria but did not contain compounds and was treated as a negative control. MIC values were determined by visual inspection of the minimum concentration that prevented bacteria growth after 18 h static incubation at 37 °C.

### Structure elucidation of prolinolexin

Purified **1** (Supplementary Fig. 3) was determined to have a molecular formula of C_43_H_74_N_6_O_9_ based on *m/z* 819.5532 (Supplementary Fig. 4), which represents its protonated adduct (calculated mass for C_43_H_75_N_6_O_9_^+^ = 819.5590, Δ = 7.1 ppm). This formula requires 10 degrees of unsaturation. Analysis of the ^1^H NMR spectrum (Supplementary Fig. 5) revealed an apparent mixture of isomers based on pairs of resonances existing at an ~6:4 ratio in several regions of the spectrum (e.g., 10.06–10.08 ppm and 4.84–4.86 ppm). These peaks did not coalesce when ^1^H NMR spectra were collected in CD_3_OD at −35, −20, −5, 10, 25, 35, 45, and 55 °C. Compound **1** was characterized as a mixture of these isomers using MS/MS. By comparing the Stachelhaus codes of each A domain in the *plx* gene cluster to a manually constructed database of codes with known substrates, the following peptide sequence was predicted for **1**: lipid-*N*-Me-Val_1_-Ser_2_-Pro_3_-Ala_3_-MeAsp_4_-Pro_5_-OH (Supplementary Fig. 7a). The first and last modules in the NRPS assembly line were assigned based on the presence of a C_START_ domain in Plx19 and a thioreductase domain in Plx20. Plx2 is presumed to reduce the aldehyde-containing intermediate released from the NRPS to **1**. Detailed analysis of the MS^2^ spectrum of **1** (Supplementary Fig. 7b) revealed the following peptide sequence: lipid-*N*-Me-Val_1_-Dha_2_-Pro_3_-Ala_3_-[MeAsp/Glu]_4_-Pro_5_-OH where the acylium ion of the lipid tail (b_0_) has a molecular formula of C_16_H_31_O^+^ (observed *m/z* 239.2375, Δ = 2.5 ppm). This empirical peptide sequence is consistent with our bioinformatic prediction of **1** (Dha is well known to originate from dehydration of Ser after the latter is loaded onto its T domain). Marfey’s analysis of **1** (Supplementary Fig. 8) confirmed the presence of Glu in **1**, leading to the peptide sequence assignment of lipid-*N*-Me-Val_1_-Dha_2_-Pro_3_-Ala_3_-Glu_4_-Pro_5_-OH. To identify the fatty acid appended to **1**, a fatty acid methyl ester (FAME) was prepared from the hydrolysate of **1** (Supplementary Fig. 9a). The **1**-derived FAME was compared by GC-MS to FAMEs of three candidate fatty acids—palmitic acid, 13-methylpentadecanoic acid, and 14-methylpentadecanoic acid. Compound **1** was determined to be appended with 14-methylpentadecanoic acid to generate the final structure of **1** depicted in Fig. 2 (Supplementary Fig. 9b). The assignment of an *iso-*fatty acyl moiety is supported by the 2D NMR data for **1** presented in Supplementary Fig. 9c. We suspect the apparent duplicated signals in the ^1^H NMR spectrum to arise from *cis-trans* isomerization of the Pro-OH amide bond in **1**, as is seen with the prolinol-containing natural product viritidin^22^. The structure of **1** is consistent with the molecular formula C_43_H_74_N_6_O_9_ and its required degrees of unsaturation.

Marfey’s method^23^ was performed as follows. Compound **1** (0.2 mg) and Fmoc-D-Glu (1.0 mg, P3 BioSystems) were dissolved separately in 1 mL of 6 N and stirred at 100 °C for 2 h. Both mixtures were dried *in vacuo*. The dried hydrolysates of **1** (0.05 mg) and Fmoc-D-Glu (1.0 mg), and L-Glu (1.0 mg, Sigma) were separately suspended in 0.15 mL deionized H_2_O, 0.3 mL N_α_-(2,4-dinitro-5- fluorophenyl)-L-alaninamide (L-FDAA, Sigma, 10 mg/mL in acetone) and 0.07 mL 1 M NaHCO_3_ (aq). Each reaction mixture was incubated at 37 °C for 2 h and dried in vacuo, resuspended in 0.5 mL CH_3_OH and then diluted 10-fold with H_2_O. Each diluted sample was analyzed by LC-HRMS using the following chromatographic conditions: linear gradient from 5 to 50% B from 10 to 45 min, linear gradient from 50 to 95% B from 45 to 47.5 min, 95% B from 47.5 to 52.5 min, linear gradient from 95% B to 5% B from 52.5 to 53 min, 5% B from 53 to 60 min (flow rate = 0.4 mL/min).

FAME analysis of the fatty acyl moiety in **1** was performed as follows. 13-Methylpentadecanoic acid (1.0 mg, Larodan), palmitic acid (1.0 mg, Sigma), and the dried hydrolysate of **1** (0.05 mg) were dissolved separately in 0.2 mL toluene, 1.5 mL CH_3_OH and 0.3 mL of 8.0% *w/v* HCl in CH_3_OH then incubated at 100 °C for 1 h. Each reaction mixture was cooled to room temperature, and then 1 mL of H_2_O and 1 mL of hexanes were added to each. The reaction mixtures were vortexed, and the upper (hexanes) layer of each was removed to use as a GC-MS sample. The following oven ramp method was used: 100 °C from 0 to 1 min, 100 °C to 190 °C from 1 to 9.5 min, 190 °C to 220 °C from 9.5 to 25.5 min, and 220 °C to 280 °C from 35.5 to 31.5 min. Carrier gas (He) flow = 1 mL/min and reagent gas (methane) flow = 1 mL/min (20%). The FAMEs of 14-methylpentadecanoic acid, 13-methylpentadecanoic acid, and palmitic acid were all chromatographically resolved with baseline resolution (t_R_ = 12.550, 12.705, and 13.239 min, respectively).

### Structure elucidation of cinnamexin

Purified **2** was determined to have a molecular formula of C_62_H_81_N_11_O_21_ based on *m/z* 1298.5541, which represents its [M + H-H_2_O]^+^ adduct (calculated mass for C_62_H_80_N_11_O_20_^+^ = 1298.5576, Δ = 2.7 ppm). This formula requires 28 degrees of unsaturation. All NMR data was initially collected in acetone-*d*_*6*_. The COSY NMR spectrum of **2** revealed 16 spin systems that, combined with HMBC NMR correlations, established the 11 partial structures i-xi depicted in Supplementary Fig. 22. Crowding and overlapping of ^13^C resonances ranging from ~169-175 ppm precluded their definitive assignment from spectral data of **2** collected in acetone-*d*_*6*_. The corresponding signals were found to be much better resolved when **2** was dissolved in methanol-*d*_*4*_. HMBC NMR spectra collected in methanol-*d*_*4*_ enabled substructures i-xi to be connected to generate the three new substructures xii, xiii, and xiv (Supplementary Fig. 22). While no HMBC correlations connecting xii, xiii, and xiv were observed, the complete peptide backbone sequence of **2** could be readily inferred bioinformatically from *cmx* (Supplementary Fig. 23). By comparing the Stachelhaus codes of each A domain in the *cmx* gene cluster to a manually constructed database of codes with known substrates, the following peptide sequence was predicted for **2**: Thr_1_-Hpg_2_-Thr_3_-Gly_4_-Leu_5_-Pro_6_-Leu_7_-Gly_8_-Tyr_9_-Asn_10_. The first and last modules in the NRPS assembly line were assigned based on the presence of a C_START_ domain in Cmx34 and thioesterase domain in Cmx31. The partial structures xii-xiv map to this predicted sequence as shown in Supplementary Fig. 23 to generate the proposed peptide sequence of **2** represented by substructure (xv). The β-hydroxyl substituents of HyLeu_5_, HyGly_8_, and HyTyr_9_ are proposed to be installed after Tyr, Gly, and Leu have been loaded onto their respective T domains. A four-bond HMBC correlation supporting the crosslink between HyTyr_9_ and Hpg_2_ could not be detected. However, the ^1^H and ^13^C chemical shifts of this proposed biaryl ether motif are consistent with those of the structurally related natural product K-13^24^ (Supplementary Fig. 24). Furthermore, placing additional atoms on substructure xv exceeds the observed molecular weight of **2**. The structure of **2** is consistent with the molecular formula C_62_H_81_N_11_O_21_ and its required degrees of unsaturation. Tabulated 1D and 2D NMR data are described in Supplementary Tables 6 and 7.

### Structure elucidation of conkatamycin

Purified **3** was determined to have a molecular formula of C_39_H_63_N_3_O_9_ based on *m/z* 718.4568, which represents its protonated adduct (calculated mass for C_39_H_64_N_3_O_9_^+^ = 718.4637, Δ = 9.6 ppm). This formula requires 10 degrees of unsaturation. The COSY NMR spectrum of **3** revealed four apparent spin systems (i–iv). The connectivity of spin systems i–iv to one another was determined using the key HMBC correlations depicted in Supplementary Fig. 31a. The presence of a guanidino group was established by an HMBC correlation from H-32 (3.17 ppm) to carbon with no attached protons and a diagnostic chemical shift of 158.9 ppm (C-33). The chemical shift of C-33 is consistent with analogous quaternary carbons in reported guanidinyl-containing polyketides (e.g., 157.7 ppm in ECO-0501^15^, 158.29 ppm in copiamycin^25^, and 158.66 ppm in clethramycin^26^). The constitution of the saccharide was determined by NMR spectroscopy, mass spectrometry, the presence of specific biosynthetic genes in the *ktm* cluster, and Tanaka’s method. COSY NMR correlations involving H-35, H-36, H-37, and H-38 were obscured by the solvent signal; however, their connectivity was deduced by HMBC correlations. The assigned ^1^H and ^13^C chemical shifts for positions 35-38 are in agreement with the corresponding positions of the glucuronic acid (GlcA) moiety in ECO-0501^15^. While no HMBC correlations to C-38 were observed, the presence of a carboxylic acid at this position satisfies the molecular formula C_39_H_63_N_3_O_9_ and its required degrees of unsaturation. The HRMS fragment *m/z* 524.4203 (Supplementary Fig. 32) corresponding to the protonated aglycone of **3**, indicates the loss of a substituent with the same exact mass as GlcA. The *ktm* cluster encodes a putative UDP-glucose 6-dehydrogenase (Ktm30) predicted to generate the UDP-GlcA substrate for the predicted UDP-glucuronosyl transferase Ktm28. Finally, LC-HRMS comparison of the chemically derivatized saccharide cleaved from **1** (using Tanaka’s method) had the same retention time as the analogously derivatized D-GlcA. The structure of **3** annotated with all non-redundant HMBC correlations is depicted in Supplementary Fig. 31b, and tabulated 1D & 2D NMR data is described in Supplementary Table 9.

Tanaka’s method was performed as follows^27,28^. Compound **3** (0.01 mg) was hydrolyzed by the addition of 10 µL THF, 40 µL H_2_O and 50 µL 2 N HCl (aq) followed by overnight incubation at 100 °C. The reaction mixture was dried *in vacuo*. 50 µL of D-cysteine methyl ester (D-CME, 1 mg/mL in pyridine, TCI Chemicals) was added to the dried hydrolysate of **3** (0.01 mg), followed by 0.5 µL of neat *o*-tolyl isothiocyanate (Alfa Aesar). The reaction mixture was stirred at 60 °C for 1 h, dried in vacuo, and resuspended in CH_3_OH to a concentration of 0.5 mg/mL. D-GlcA (0.1 mg, Sigma) was subjected to the same reaction conditions as above and also with substitution of D-CME for L-cysteine methyl ester (L-CME, 1 mg/mL in pyridine, TCI Chemicals). All three samples (**3**-D-CME, GlcA-D-CME, and GlcA-L-CME) were analyzed by LC-HRMS using the following chromatographic conditions: 2.5% B from 0 to 2.5 min, linear gradient from 2.5% B to 30% B from 2.5 to 40 min, linear gradient from 30% B to 95% B from 40 to 42.5 min, 95% B from 42.5 to 50 min, linear gradient from 95% B to 2.5% B from 50 to 52.5 min and 2.5% B from 52.5 to 60 min (flow rate = 0.3 mL/min).

### Raising mutants

Attempts to raise conkatamycin (**3**) resistant mutants of *S. aureus* USA300 were carried out by plating on agar containing different antibiotic concentrations. A single colony of *S. aureus* USA300 was inoculated in 5 mL LB broth and grown overnight at 37 °C with continuous shaking (200 rpm). The overnight culture was washed with fresh LB broth and then diluted to 10^8^ cells/10 µL. Two milliliters of LB agar solution (50 °C) containing 4× and 8× conkatamycin was poured into 24-well plate (Corning Incorporated, USA). 10 µL of diluted cells was then plated onto an LB agar plate to give 10^8^ cells/well. >24 wells were seeded in each study. Plates were statically incubated at 37 °C and examined over the next 72 h for the appearance of resistant colonies. Attempts were carried out at pH 7 and pH 5.

### Membrane lysis assay

The lysis activity of conkatamycin (**3**) was tested using SYTOX green nucleic acid stain (Thermo Fisher, USA). 1% of Triton x100 (in PBS) and PBS were used as a positive and negative control, respectively. A single colony of *S. aureus* USA300 was inoculated in 5 mL LB broth and grown overnight at 37 °C with shaking (200 rpm). The overnight culture was diluted in fresh PBS to give an OD_600_ nm of 0.35. The cell suspension was then mixed with 50 uM SYTOX solution (9:1) and incubated at room temperature for 10 min. Thirty microliters of the mixture was then transferred into a 384-well flat bottom black microtiter plate. The initial fluorescence intensity of each well was recorded using an infinite 200 PRO (Excitation/Emission = 488/523 nm) at 9 s intervals for 10 min. 30 µL of a 16× MIC conkatamycin stock or 30 µL of a control solution was then added to each well. The fluorescence was then continually monitored for an additional 20 min. Data points were plotted using Prism 9.0. All assays were performed three independent times (*n* = 3).

### Membrane depolarization assay

The depolarization effect of conkatamycin (**3**) was evaluated using DiSC_3_(5) dye (Thermo Fisher, USA). Melittin and PBS were used as positive and negative control, respectively. An overnight culture of *S. aureus* USA300 was washed three times with PBS and then diluted to an OD_600_ nm of 0.35. The cell suspension was mixed (100:1) with 4 uM DiSC_3_(5) solution. After incubating at room temperature for 15 min, 30 µL of this mixture was transferred into the individual well of a 384-well flat bottom black microtiter plate. The initial fluorescence intensity of each well was measured using an infinite 200 PRO (Excitation/Emission=OD_620_ nm/OD_670_ nm) at 9 s intervals for 10 min. 30 µL of an 8× or 16× MIC stock of each compound was then added to each well. The fluorescence intensity of each well was measured for additional 20 min. Data points were plotted using Prism 9.0. All assays were performed three independent times (*n* = 3).

### UDP-MurNAc-pentapeptide accumulation assay

The effect of conkatamycin (**3**) on lipid II biosynthesis was investigated by tracking the accumulation of UDP-MurNAC-pentapeptide in antibiotic-treated cultures. *S. aureus* USA300 was used in this study. A single colony of *S. aureus* USA300 was inoculated into 5 mL fresh LB broth and grown overnight with shaking (200 rpm) at 37 °C. The overnight culture was then inoculated into fresh LB medium (1/1000) and grown to an OD_600_ nm of 0.5. Cultures were then treated with 130 ug/mL of chloramphenicol for 15 min. 5× the MIC of conkatamycin (**3**) or vancomycin was then added to the cell suspension. After incubating at 37 °C for 60 min, 0.5 mL of cells were harvested by centrifugation and resuspended in 30 µL ddH2O. Cell suspensions were then incubated in boiling water for 15 min and centrifuged at 15,000 × *g* for 15 min. The resulting supernatants were analyzed by UPLD-DAD-MS for the presence of UDP-MurNAC-pentapeptide: Acquity UPLC BEH C18, 2.1×50 mm, 1.7 µM, 130 Å, 0.45 mL/min isocratic elution at 97% water:acetonitrile (ACN) for 1 min, then from 97% to 5% water:ACN over 3 min, with constant 0.1% formic acid; negative ionization mode.

### Feeding assays

The effects of cell wall components on conkatamycin’s antimicrobial activity were investigated using *S. aureus* USA300 and general MIC assay methods described above with the following changes. The pH of the LB broth used in the feeding assay was adjusted to pH = 5.0 with 1 M HCl. 40 µL of pH-adjusted LB was added to each well of a 96-well microtiter plate. Concanamycin was then serially diluted across the plate (40 µL/well) as described above. Ten microliters of a cell wall component stock (10× the final concentration) was then added to each assay well. Fifty microliters of an overnight culture of *S. aureus* USA300 was diluted 5000-fold and added to each well to give a final volume of 100 µL per well. All assays were run in duplicate (*n* = 2) and repeated two independent times (*n* = 2).

### Reporting summary

Further information on research design is available in the Nature Research Reporting Summary linked to this article.

## Supplementary information

Supplementary Information

Reporting Summary

## Data Availability

Sequence data that support the findings of this study have been deposited in GenBank with the accession numbers OL452059, OL452060, OL452061, and OL452062. The 15 whole genomes used as reference in the study are available on GenBank with the accession numbers NZ_CP012382.1, NZ_CP086102.1, NZ_BMSY00000000.1, NZ_BMWF00000000.1, NZ_BMVE00000000.1, NZ_BNBZ00000000.1, NZ_BLIR00000000.1, NZ_BMSC00000000.1, NZ_BMTO00000000.1, NZ_BNBT00000000.1, NZ_KQ948550.1, NZ_BMSE00000000.1, NZ_BMSO00000000.1, NZ_BMSZ00000000.1, and NZ_BMUW00000000.1. Source data are provided with this paper.

## Source data

Source Data

## Code availability

Code used in this study is available at GitHub [https://github.com/VincentLibis/multiplexed_BGC_recovery].
