# Supplementary material for: Multiplexed mobilization and expression of biosynthetic gene clusters
Source: Nat Commun. 2022 Sep 6;13:5256. doi: 10.1038/s41467-022-32858-0 (PMC9448795; doi:10.1038/s41467-022-32858-0)
Supplement: Supplementary file 1 — Supplementary Information [file 41467_2022_32858_MOESM1_ESM.pdf]

Multiplexed mobilization and expression of biosynthetic gene clusters

Libis *et al.*

Supplementary Note 1. Differences between the current study and the original cosmid-based CONKAT-seq

CONKAT-seq was originally used to study the BGCs present in complex metagenomes[1]. While the core concept of detecting BGCs through partitioning of genome fragments and detection of co-occurring biosynthetic gene amplicons remains unchanged, several advances have been introduced in this work. The focus of the original approach was to detect and access the biosynthetic potential of uncultured bacteria. The sheer diversity of a soil metagenome requires the construction of clone libraries containing typically more than 10 million clones, which can currently only be achieved by using cosmid vectors. While providing access to uncultured genomes, cosmid libraries limit the rate at which BGCs can be mobilized into new hosts for several reasons. For example, the isolation of a cosmid of interest from a large cosmid library requires multiple tedious rounds of dilutions and PCR screenings. In addition, the relatively small size of cosmids (40 kb) typically necessitates the recovery of several overlapping cosmids that must be re-assembled by transformation assisted recombination (TAR) into a shuttle vector prior to being introduced into a heterologous host. These two rate limiting steps have been removed in the current work to enable a parallelization of the heterologous expression process and the generation of economies of scale. In the current approach large intact BGCs are cloned directly into a final vector in a single step, libraries are arrayed as single clones allowing for immediate access without a PCR screening step and lastly, unlike metagenomic libraries, the current implementation benefits from knowing the phylum of the donor strains which translates into higher activation rate by using closely related heterologous hosts.

Supplementary Table 1. List of *Streptomyces* species in the collection.*

1	*S. achromogenes*
2	*S. albaduncus*
3	*S. albidoflavus*
4	*S. albogriseolus*
5	*S. albolungus*
6	*S. almquistii*
7	*S. amakuensis*
8	*S. ambofaciens*
9	*S. anandii*
10	*S. antibioticus*
11	*S. anulatus*
12	*S. argenteolus*
13	*S. atroolivaceus*
14	*S. aureoverticillatus*
15	*S. avermitilis*
16	*S. avicennae*
17	*S. baarensis*
18	*S. badius*
19	*S. bangladeshiensis*
20	*S. bellus*
21	*S. bikiniensis*
22	*S. bottropensis*
23	*S. cacoi*
24	*S. canus*
25	*S. capoamus*
26	*S. carpineus*
27	*S. catanulae*
28	*S. cinereus*
29	*S. colombiensis*
30	*S. corchorusii*
31	*S. cremeus*
32	*S. demanii*
33	*S. diastatochromogenes*
34	*S. durhamensis*
35	*S. endus*
36	*S. enissocaensis*
37	*S. erythrogriseus*
38	*S. felleus*
39	*S. flavidovirens*
40	*S. flocculus*
41	*S. galbus*
42	*S. gelaticus*
43	*S. geyseriensis*
44	*S. ghanaensis*
45	*S. globisporus*
46	*S. goshkiensis*
47	*S. gougerotii*
48	*S. griseiniger*
49	*S. griseofuscus*
50	*S. griseostraminus*

51	*S. griseoviridus*
52	*S. griseus*
53	*S. halstedii*
54	*S. hawaiiensis*
55	*S. helvaticus*
56	*S. humidus*
57	*S. hygroscopicus*
58	*S. labedae*
59	*S. lateritus*
60	*S. lavendulae*
61	*S. levis*
62	*S. lilacinus*
63	*S. longisporoflavus*
64	*S. longispororuber*
65	*S. longwoodensis*
66	*S. lusitanus*
67	*S. macrosporeus*
68	*S. mashaensis*
69	*S. massasporeus*
70	*S. melanosporofaciens*
71	*S. mexicanus*
72	*S. noboritiensis*
73	*S. pactum*
74	*S. pathocidini*
75	*S. pilosus*
76	*S. pluricoloroscens*
77	*S. puniceus*
78	*S. pyridomyceticus*
79	*S. ramulosus*
80	*S. racechromogenes*
81	*S. rubiogriseus*
82	*S. seoulensis*
83	*S. sp. F8211*
84	*S. sp. S-350*
85	*S. sparsogenes*
86	*S. speibona*
87	*S. spheroides*
88	*S. spiralis*
89	*S. sulfonofaciens*
90	*S. terminatum*
91	*S. thermocoprohilus*
92	*S. tubercidius*
93	*S. violaceolatus*
94	*S. violaceusniger*
95	*S. viridochromogenes*
96	*S. xanthachromogenes*
97	*S. xinghaensis*
98	*S. yataensis*
99	*S. zaomycetius*
100	*S. ennissocaesilis*

*Strains were obtained from either ATCC or the Agricultural Research Service Culture (ARCS/NRRL) Collection.

Supplementary Table 2. Lydiamycin A biosynthetic gene analysis.

Gene	Proposed function	NCBI similarity	Species	%ID
lyd1	Regulation	helix-turn-helix domain-containing protein	*Streptomyces aureoverticillatus*	100
lyd2		peptide deformylase	*Streptomyces aureoverticillatus*	100
lyd3		NAD(P)/FAD-dependent oxidoreductase	*Streptomyces aureoverticillatus*	100
lyd4	NRPS	non-ribosomal peptide synthetase	*Streptomyces venezuelae*	79
lyd5		methylmalonyl-CoA mutase family protein	*Streptomyces aureoverticillatus*	100
lyd6		acyl carrier protein	*Streptomyces aureoverticillatus*	100
lyd7		AMP-binding protein	*Streptomyces aureoverticillatus*	100
lyd8		condensation domain-containing protein	*Streptomyces aureoverticillatus*	100
lyd9		crotonyl-CoA carboxylase/reductase	*Streptomyces aureoverticillatus*	100
lyd10		methylmalonyl-CoA mutase	*Streptomyces aureoverticillatus*	100
lyd11		methylmalonyl Co-A mutase-associated GTPase MeaB	*Streptomyces aureoverticillatus*	100
lyd12		lysine N(6)-hydroxylase/L-ornithine N(5)-oxygenase family protein	*Streptomyces aureoverticillatus*	100
lyd13	Regulation	FMN-binding negative transcriptional regulator	*Streptomyces aureoverticillatus*	100
lyd14	Regulation	helix-turn-helix transcriptional regulator	*Streptomyces aureoverticillatus*	100
lyd15		alpha/beta hydrolase	*Streptomyces aureoverticillatus*	100
lyd16	Regulation	LysR family transcriptional regulator	*Streptomyces aureoverticillatus*	100

Supplementary Table 3. Prolinolexin (1) biosynthetic gene analysis.

Gene	Proposed function	NCBI similarity	Species	%ID
plx1		hypothetical protein	Streptomyces purpurascens	100
plx2		FAD-binding oxidoreductase	*Streptomyces purpurascens*	100
plx3	Regulation	PadR family transcriptional regulator	*Streptomyces purpurascens*	100
plx4	Transport	ABC transporter ATP-binding protein	*Streptomyces venezuelae*	79
plx5	Transport	ABC transporter permease	*Streptomyces purpurascens*	100
plx6		helix-turn-helix domain-containing protein	*Streptomyces purpurascens*	100
plx7		DUF475 domain-containing protein	*Streptomyces purpurascens*	100
plx8		DUF2975 domain-containing protein	*Streptomyces purpurascens*	100
plx9		SDR family oxidoreductase	*Streptomyces purpurascens*	100
plx10	Regulation	TetR/AcrR family transcriptional regulator	*Streptomyces purpurascens*	100
plx11		hypothetical protein GCM10010303_80280	*Streptomyces purpurascens*	98
plx12		-	-	<30
plx13		alpha/beta hydrolase	*Streptomyces purpurascens*	100
plx14		phytanoyl-CoA dioxygenase family protein	*Streptomyces purpurascens*	100
plx15		restriction endonuclease	*Streptomyces purpurascens*	100
plx16		hypothetical protein GCM10010303_80340	*Streptomyces purpurascens*	100
plx17		hypothetical protein GCM10010303_80340	*Streptomyces purpurascens*	100
plx18		NAD(P)-dependent oxidoreductase	*Streptomyces purpurascens*	100
plx19	NRPS	non-ribosomal peptide synthetase	*Streptosporangium nondiastaticum*	83
plx20	NRPS	amino acid adenylation domain-containing protein	*Streptomyces klenkii*	81
plx21		MbtH family protein	*Streptomyces purpurascens*	98
plx22		FAD-dependent monooxygenase	*Streptomyces purpurascens*	100
plx23		hypothetical protein	*Streptomyces purpurascens*	100
plx24		CGNR zinc finger domain-containing protein	*Streptomyces purpurascens*	100
plx25		epoxide hydrolase	*Streptomyces purpurascens*	100
plx26		VOC family protein	*Streptomyces purpurascens*	100
plx27	Regulation	XRE family transcriptional regulator	*Streptomyces violarus*	94
plx28	Regulation	response regulator	*Streptomyces purpurascens*	100
plx29	Regulation	histidine kinase	*Streptomyces purpurascens*	100

Supplementary Table 4. MS^2 fragment analysis for prolinolexin.

Ion	Observed	Calculated	Δ in ppm
b_0	239.2375	239.2369	2.5
b_1	352.3184	352.3210	7.4
b_2	421.3432	421.3425	1.7
b_3	518.3941	518.3952	2.1
b_4	589.4325	589.4323	0.3
b_5	718.4740	718.4749	1.3
y_0	n.o.	581.3293	N/A
y_1	468.2494	468.2453	8.8
y_2	399.2230	399.2238	2.0
y_3	n.o.	302.1270	N/A
y_4	231.1356	231.1339	7.4
y_5	102.0915	102.0913	2.0
a_1	324.3280	324.3261	5.9
z_2	451.2232	451.2187	10.0
$[M+H]^+$	819.5633	819.5590	5.2
$[M+H-H_2O]^+$	801.5506	801.5484	2.7

*Observed as in-source fragmentation in MS^1.
n.o. = not observed

Supplementary Table 5. Cinnamexin (2) biosynthetic gene analysis.

Gene	Proposed function	NCBI similarity	Species	%ID
cmx1	Regulation	LysR family transcriptional regulator	*Streptomyces antioxidans*	100
cmx2	Regulation	response regulator transcription factor	*Streptomyces melanosporofaciens*	100
cmx3	Regulation	LuxR family transcriptional regulator	*Streptomyces antimycoticus*	100
cmx4		alpha/beta fold hydrolase	*Streptomyces antimycoticus*	99
cmx5	Regulation	DNA-binding transcriptional regulator, IclR family	*Streptomyces melanosporofaciens*	100
cmx6	Regulation	DNA-binding transcriptional activator of the SARP family	*Streptomyces melanosporofaciens*	99
cmx7		hypothetical protein	*Streptomyces melanosporofaciens*	99
cmx8		hypothetical protein	*Streptomyces melanosporofaciens*	99
cmx9		acyl carrier protein	*Streptomyces hygroscopicus*	99
cmx10		beta-ketoacyl-[acyl-carrier-protein] synthase family protein	*Streptomyces sp. 4503*	99
cmx11		3-oxoacyl-ACP synthase	*Streptomyces melanosporofaciens*	100
cmx12		3-oxoacyl-ACP synthase	*Streptomyces melanosporofaciens*	100
cmx13		alpha/beta hydrolase	*Streptomyces melanosporofaciens*	100
cmx14		hypothetical protein	*Streptomyces melanosporofaciens*	100
cmx15		3-oxoacyl-ACP synthase	*Streptomyces melanosporofaciens*	100
cmx16		3-hydroxyacyl-[acyl-carrier-protein] dehydratase	*Streptomyces melanosporofaciens*	100
cmx17		beta-hydroxyacyl-ACP dehydratase	*Streptomyces melanosporofaciens*	100
cmx18		3-oxoacyl-[acyl-carrier-protein] reductase	*Streptomyces antimycoticus*	100
cmx19		DsbA family protein	*Streptomyces melanosporofaciens*	100
cmx20		MbtH family protein	*Streptomyces (multispecies)*	100
cmx21		AarF/UbiB family protein	*Streptomyces melanosporofaciens*	100
cmx22		prephenate dehydrogenase	*Streptomyces melanosporofaciens*	100
cmx23		aminotransferase class I/II-fold pyridoxal phosphate-dependent enzyme	*Streptomyces melanosporofaciens*	100
cmx24		4-hydroxyphenylpyruvate dioxygenase	*Streptomyces melanosporofaciens*	100
cmx25		cytochrome P450	*Streptomyces melanosporofaciens*	100
cmx26	Transport	ATP-binding cassette domain-containing protein	*Streptomyces melanosporofaciens*	100
cmx27	Transport	ABC transporter permease	*Streptomyces iranensis*	98
cmx28		LLM class flavin-dependent oxidoreductase	*Streptomyces antimycoticus*	99
cmx29		cytochrome P450	*Streptomyces antimycoticus*	99
cmx30	NRPS	non-ribosomal peptide synthetase	*Streptomyces melanosporofaciens*	100
cmx31	NRPS	non-ribosomal peptide synthetase	*Streptomyces melanosporofaciens*	100

cmx32		cytochrome P450	*Streptomyces melanosporofaciens*	100
cmx33		hypothetical protein	*Streptomyces melanosporofaciens*	100
cmx34		non-ribosomal peptide synthase domain TIGR01720/amino acid adenylation domain-containing protein	*Streptomyces melanosporofaciens*	100
cmx35		flavin reductase family protein	*Streptomyces melanosporofaciens*	100
cmx36		MBL fold metallo-hydrolase	*Streptomyces violaceusniger*	100
cmx37		S-(hydroxymethyl)mycothiol dehydrogenase	*Streptomyces melanosporofaciens*	100
cmx38		M4 family metallopeptidase	*Streptomyces melanosporofaciens*	100
cmx39		NAD(P)-dependent alcohol dehydrogenase	*Streptomyces melanosporofaciens*	100
cmx40	Regulation	TetR family transcriptional regulator	*Streptomyces melanosporofaciens*	99

Supplementary Table 6. NMR spectroscopic data of cinnamexin (2) in acetone-d6 (1H: 600MHz, 13C:151 MHz).

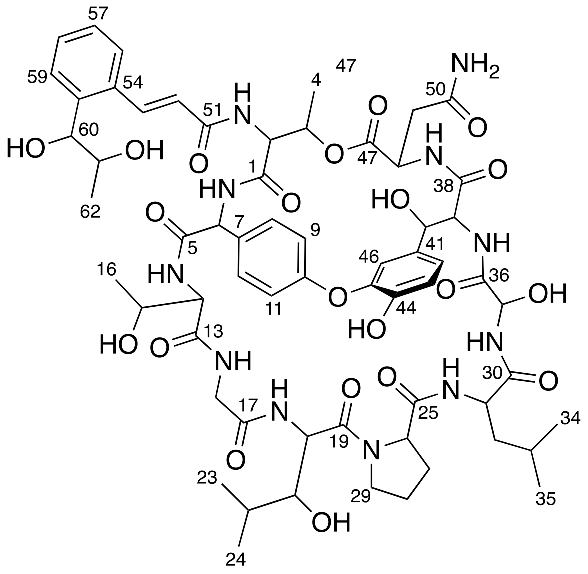

Residue	Position	δ_C	Type	δ_H (*J* in Hz)	COSY	^{1}H-^{13}C HMBC
Thr$_1$	1	174.5	C			
	2	62.7	CH	5.06, d (3.4)	3, Thr$_1$-NH	1
	3	70.1	CH	5.32, q (6.5)	2, 4	1, 4
	4	18.2	CH_3	1.32, d (6.6)	3	2, 3
	NH			9.18 d (3.2)	1	2, 3
Hpg$_2$	5	173.5	C			
	6	61.7	CH	5.03, d(3.1)	Hpg$_9$-NH	7, 8, 12
	7	133.7	C			
	8	129.9	CH	7.27, d (8.6)	9	12, 10
	9	124.8	CH	6.58, dd(8.6, 1.6)	8	7, 10, 9
	10	161.0	C			
	11	123.4	CH	7.27, d (8.6)	12	9, 7
	12	132.3	CH	7.44, app. d (9.1)	11	6, 8, 10
	NH			7.63, d(2.9)	6	7
Thr$_3$	13	173.1	C			
	14	58.7	CH	4.68, m	15, Thr$_3$-NH	13, 16
	15	68.0	CH	4.59, m	14, 16, 15-OH	
	16	20.7	CH_3	1.24, d (6.4)	15	14, 15
	NH			8.35, m	14	
	15-OH			3.84, d (6.4)	15	14, 15, 16
Gly$_4$	17	172.8	C			
	18a	43.9	CH_2	3.42, d (17.2, 4.9)	18b, Gly$_4$-NH	
	18b	43.9	CH_2	4.32, d (17.2, 8.0)	18a, Gly$_4$-NH	
	NH			7.89, app. t (6.2)	18a, 18b	18
HyLeu$_5$	19	171.9	C			
	20	53.8	CH	4.80, m	21, HyLeu$_7$-NH	21, 22
	21	76.2	CH	3.41, m	20, 21-OH	20, 23, 24
	22	29.3	CH	1.88, m	21, 23, 24	21, 23, 24
	23	14.8	CH_3	0.81, d (6.8)	22	21, 22, 24
	24	21.0	CH_3	0.86, d (6.8)	22	21, 22, 23
	NH			7.70, d (9.0)	20	20, 21
	21-OH			7.89, app. t (6.2)	21	
Pro$_6$	25	171.5	C			
	26	61.7	CH	4.48, app. t (7.1)	27a, 27b	27, 28, 29
	27a	31.1	CH_2	1.80, m	26, 27b	26, 28, 29
	27b	31.1	CH_2	2.36, m	26, 27a, 28	26, 28, 29
	28	25.8	CH_2	1.90, m	27b, 29a, 29b	26, 27, 29
	29a	48.4	CH_2	3.63, m	28, 29b	27
	29b	48.4	CH_2	3.89, app. q (7.4)	28, 29a	27, 28
Leu$_7$	30	171.4	C			
	31	51.9	CH	4.67, m	Leu$_2$-NH, 32b	
	32a	44.4	CH_2	1.42, m	31, 32b, 33	31, 33, 34, 35
	32b	44.4	CH_2	1.51, m	31, 32a	31, 33, 34, 35
	33	25.4	CH	1.59, m	34, 35	31, 32, 34, 35
	34	22.4	CH_3	0.89, app. t (5.9)	33	35
HyGly$_8$	36	170.9	C			
	37	73.1	CH	6.03, app. d (9.1)	37-OH, HyGly$_8$-NH	
	NH			8.32, d (9.1)	37	
	37-OH			6.35, s (br)	37	37
HyTyr$_9$	38	170.3	C			
	39	65.1	CH	3.66, m	40, HyTyr$_5$-NH	40, 41
	40	71.6	CH	4.95, d (3.4)	39, 40-OH	39, 41, 46
	41	133.3	C			
	42	121.4	CH	7.18, m	43	40, 44, 46
	43	116.8	CH	6.90, d (8.4)	42	20, 44
	44	148.7	C			
	45	148.7	C			
	46	121.0	CH	6.39, s		40, 42, 44
	NH			8.34, m	39	
	40-OH			5.30, d (6.3)	40	39
	44-OH			8.22, s		43, 44
Asn$_{10}$	47	170.2	C			
	48	50.5	CH	4.21, m	49, Asn$_{10}$-NH	49, 50
	49	35.9	CH_2	2.80, obs	48	48, 50
	50	172.0	C			
	NH			8.03, d (7.3)	48	48
	$CONH_2$			6.41, s (br)		49
Cinn	51	169.5	C			
	52	125.2	CH	6.99, d(15.3)	53	51, 54
	53	138.6	CH	8.16, d(15.5)	52	51, 52, 54, 55, 59
	54	135.5	C			
	55	128.2	CH	7.52, d (7.7)	56	57, 59
	56	128.9	CH	7.06, m	55, 57	58, 54
	57	130.5	CH	7.31, app. t (7.5)	56, 58	55, 59
	58	125.5	CH	7.16, m	57	54, 56, 60
	59	138.2	C			
	60	56.3	CH	4.07, s	61, 60-OH	58, 59, 61, 62
	61	59.9	CH	2.97, q (4.8)	60, 62, 61-OH	62
	62	18.4	CH_3	1.47, d (4.8)	61	60, 61
	60-OH			n.o		
	61-OH			n.o		

^{13}C resonances for the following positions are interchangeable: 1, 5, 13, 17, 19, 25, 30, 36, 38, 47, 51. HMBC correlations to these signals were not tabulated.

Supplementary Table 7. NMR spectroscopic data of cinnamexin (**2**) in methanol-d_4 (^{1}H: 600MHz, ^{13}C:151 MHz). HMBC correlations to carbonyl signals were observed at 800 MHz (^{1}H) and 201 MHz (^{13}C).

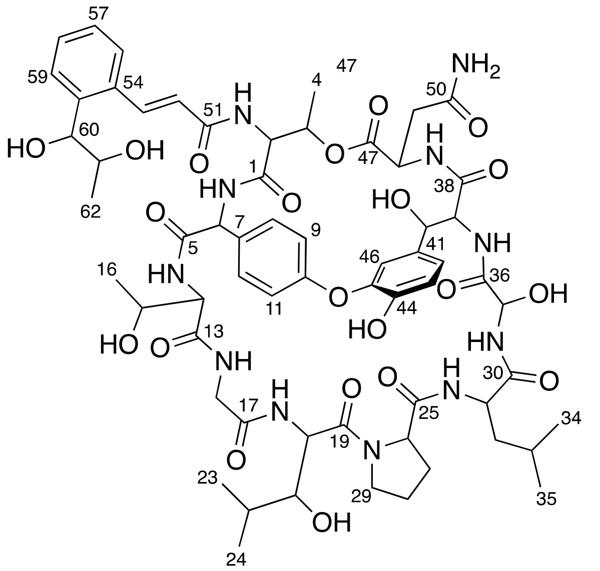

Position		δC, Type		δH (J in Hz)	COSY	^{1}H-^{13}C HMBC
	1	175.9	C			
**Thr₁**	2	63.3	CH	4.95, d (3.8)	3	1, 3, 4, 51
	3	70.8	CH	5.39, q (6.8)	2, 4	1, 4, 47
	4	18.33	CH_3	1.36, d (6.8)	3	2, 3
	5	172.81	C			
	6	62.06*	CH	5.07, d (3.3)		1, 5, 8, 12
	7	132.8*	C			
**Hpg₂**	8	133.26*	CH	7.42, dd (8.4, 2.3)	9	6, 10, 12
	9	124.3	CH	7.197, d (8.2)	8	7, 11
	10	162.3	C			
	11	125.8	CH	6.70, dd (8.5, 2.4)	12	9, 11
	12	130.7	CH	7.31, m	11	6, 8, 10
	13	172.75	C			
**Thr₃**	14	60.0	CH	4.70, m	15	13, 15, 16
	15	67.8	CH	4.62, qd (6.6, 2.7)	14, 16	13
	16	21.1	CH_3	1.24, d (6.7)	15	
	17	171.60	C			
**Gly₄**	18a	44.1	CH_2	3.64, dd (17.2, 5.6)	18b	13, 17
	18b	44.1	CH_2	4.35, dd (17.5, 7.5)	18a	13, 17
	19	172.2	C			
	20	54.5	CH	4.80, obs	21	17, 21, 22
**HyLeu₅**	21	77.3	CH	3.50, dd (10.0, 1.9)	20, 22	22, 23, 24
	22	29.8	CH	1.87, m	21, 23, 24	21, 23, 24
	23	14.9	CH_3	0.83, d (6.8)	22	21, 22, 24
	24	20.8	CH_3	0.83, d (6.8)	22	21, 22, 23
	25	173.93	C			
	26	61.99*	CH	4.40	27a, 27b	25, 27, 28, 29
	27a	31.6	CH_2	1.76, m	26, 27b, 28	26, 28, 29
**Pro₆**	27b	31.6	CH_2	2.29, m	26, 27a, 28	26, 28, 29
	28	26.1	CH_2	1.91, m	27a, 27b, 29a, 29b	26, 27, 29
	29a	49.2	CH_2	3.67, m	28, 29b	26, 27, 28
	29b	49.2	CH_2	3.86, dt (9.7, 7.1)	28, 29a	26, 27, 29
	30	173.8	C			
	31	52.4	CH	4.70, m	32a, 32b	25, 30, 32, 33
	32a	44.4	CH_2	1.50, m	31, 32b, 33	30, 31, 33
**Leu₇**	32b	44.4	CH_2	1.56, m	31, 32a, 33	31, 33
	33	25.9	CH	1.57, m	32a, 32b, 34, 35	
	34	23.4	CH_3	0.94, d(6.2)	33	32, 33, 35
	35	22.5	CH_3	0.94, d(6.0)	33	32, 33, 34
**HyGly₈**	36	172.56	C			
	37	73.4	CH	5.99, s		30, 31, 36

Position		δC, Type		δH (J in Hz)	COSY	^{1}H-^{13}C HMBC
	38	172.35	C			
	39	65.1	CH	3.60, d (10.5)	40	38, 40, 41, 48
	40	72.0	CH	4.69, d (10.5)	39	38, 40, 41, 42, 45, 46
	41	133.3	C			
**HyTyr₉**	42	121.9	CH	7.07, dd (8.5, 1.5)	43	40, 46, 44
	43	117.3	CH	6.85, d (8.5)	42	40, 41, 44, 45, 46
	44	148.9	C			
	45	149.2	C			
	46	120.7	CH	6.32, d (1.7)		40, 42, 45
	47	171.3	C			
	48	51.1	CH	4.12, m	49	42, 47, 49, 50
**Asn₁₀**	49a	36.1	CH_2	2.71, dd (16.0, 8.6)	49	47, 48, 50
	49b	36.1	CH_2	2.78, dd (16.0, 4.1)	49	47, 48, 50
	50	174.3	C			
	51	171.67	C			
	52	125.1	CH	6.93, d (15.7)	53	51, 53, 54
	53	139.9	CH	8.17, d (15,7)	52	51, 52, 54, 55
	54	136.60	C			
	55	129.2	CH	7.62, d (7.6)	56	57, 53
**Cinn**	56	129.6	CH	7.17, t (7.3)	55, 57	54, 58
	57	131.2	CH	7.31, m	56, 58	55, 59
	58	126.8	CH	7.194, d (8.2)	57	54, 56
	59	138.30	C			
	60	57.8	CH	4.10, d (2.0)	61	59, 61
	61	59.5	CH	3.11, qd (5.2, 2.0)	60, 62	59, 60, 62
	62	18.26	CH_3	1.48, d (5.2)	61	60, 61

Supplementary Table 8. Conkatamycin (3) biosynthetic gene analysis.

Gene	Proposed function	NCBI similarity	Species	%ID
ktm1		hypothetical protein	*Streptomyces sp. SID5910*	78
ktm2		alpha/beta hydrolase	*unclassified Streptomyces*	98
ktm3		NAD-dependent epimerase/dehydratase family protein	*unclassified Streptomyces*	98
ktm4		aminotransferase class I/II-fold pyridoxal phosphate-dependent enzyme	*unclassified Streptomyces*	98
ktm5		toxin	*unclassified Streptomyces*	100
ktm6		helix-turn-helix domain-containing protein	*unclassified Streptomyces*	100
ktm7		alpha/beta fold hydrolase	*unclassified Streptomyces*	99
ktm8		AAA family ATPase	*unclassified Streptomyces*	100
ktm9		thioesterase	*unclassified Streptomyces*	100
ktm10		acyl--CoA ligase	*unclassified Streptomyces*	100
ktm11		carbon-nitrogen hydrolase family protein	*unclassified Streptomyces*	100
ktm12		methylmalonyl-CoA mutase family protein	Streptomyces sp. WAC 01438	100
ktm13		cobalamin B12-binding domain-containing protein	*unclassified Streptomyces*	100
ktm14		WHG domain-containing protein	*unclassified Streptomyces*	100
ktm15		hypothetical protein	*unclassified Streptomyces*	100
ktm16		ACP S-malonyltransferase	*unclassified Streptomyces*	99
ktm17	PKS	hypothetical protein DLM49_24280	Streptomyces sp. WAC 01438	99
ktm18	PKS	SDR family NAD(P)-dependent oxidoreductase	Streptomyces sp. WAC 01438	99
ktm19	PKS	polyketide synthase	Streptomyces sp. WAC 01438	99
ktm20	PKS	type I polyketide synthase	*unclassified Streptomyces*	100
ktm21		DUF1453 family protein	*unclassified Streptomyces*	98
ktm22	Regulation	response regulator transcription factor	*unclassified Streptomyces*	100
ktm23		fatty acyl-AMP ligase	*unclassified Streptomyces*	100
ktm24		FAD-dependent oxidoreductase	*unclassified Streptomyces*	100
ktm25		MFS transporter	*unclassified Streptomyces*	100
ktm26		phosphotransferase	Streptomyces sp. WAC 01438	100
ktm27	Hexylmalonyl-CoA biosynthesis	crotonyl-CoA carboxylase/reductase	*unclassified Streptomyces*	100
ktm28	Transfer of GlcA	glycosyltransferase	*unclassified Streptomyces*	100
ktm29		PIG-L family deacetylase	*unclassified Streptomyces*	98
ktm30		nucleotide sugar dehydrogenase	Streptomyces sp. WAC 01438	99
ktm31		hypothetical protein	*unclassified Streptomyces*	100
ktm32		hypothetical protein	*unclassified Streptomyces*	100
ktm33	Regulation	histidine kinase	*unclassified Streptomyces*	100

Supplementary Table 9. NMR spectroscopic data of conkatamycin (**3**) in CD_3OD (^1H: 600MHz, ^{13}C:151 MHz).

Position	δ_C, Type		δ_H (*J* in Hz)	COSY	^1H-^{13}C HMBC
1	175.5	C			
2	138.1	C			
3	29.1	CH_2	2.42, t(7.5)	4	1,2,4,5
4	29.2	CH_2	1.44, m	3,5	2,3,7,8
5	40.4	CH_2	1.22, m	4,6	6,7,8
6	29.5	CH	1.54, m	5,7,8	5,7,8
7	23.5	CH_3	0.88, d(6.5)	6	5,6,7
8	23.5	CH_3	0.88, d(6.5)	6	5,6,8
9	137.2	CH	7.07, d(11.3)	10	1,2,3,10,11
10	129.1	CH	6.50, dd (14.7, 11.0)	9,11	12
11	139.5	CH	6.47, dd (14.7, 10.2)	10,12	12,13
12	132.3	CH	6.29 dd(14.7, 10.0)	11,13	11,14
13	137.2	CH	6.23, dd(15.4, 10.1)	12,14	14,15
14	131.0	CH	6.10, dd(15.0, 9.9)	13,15	12,13,16
15	140.0	CH	5.5, dd(14.9, 8.8)	14,16	13,16,17,18
16	41.5	CH	2.52, m	15,17,18	14,15,17,18
17	18.4	CH_3	1.19, d(6.4)	16	15,16,18
18	94.5	CH	3.60, d(9.5)	16	16,17,20,21,34
19	135.4	C			
20	12.5	CH_3	1.62, m		19,21
21	137.4	CH	4.99, d(9.5)	22	18,20,22,24
22	31.1	CH	2.46, m	21,23,24a,24b	
23	14.9	CH_3	0.90, d(6.1)	22	21,22,24
24a	46.6	CH_2	0.96, m	24b,25	
24b	46.6	CH_2	1.22, m	22,24a	
25	32.3	CH	1.35, obs	25,26,27a,27b	
26	20.0	CH_3	0.70, d(6.5)	25	24,25,27
27a	42.9	CH_2	1.78, m	26,27b,28	24,25,26,28
27b	42.9	CH_2	1.90, m	26,27a,28	24,25,26,28
28	131.9	CH	5.40, m	27a,27b,29	27,30
29	131.3	CH	5.37, m	28,30	27,30
30	31.1	CH_2	2.09, m	29,31	29,31,32
31	30.4	CH_2	1.64, m	30,32	29,30,32
32	42.1	CH_2	3.16, m	31	31,33
33	158.9	C			
34	104.0	CH	4.21, d(7.7)	35	18,36
35	75.6	CH	3.27, obs		34,37
36	77.5	CH	3.41, m		38
37	78.9	CH	3.34, obs		38
38	74.2	CH	3.40, m		36
39	n.o.[1]	C			

[1]Not observed.

Supplementary Table 10. MIC values of Conkatamycin (3) against antibiotic-resistant strains of *Staphylococcus aureus.*

**Strain**	**Phenotype (resistance)**	**MIC (μg/mL)**
S. aureus NRS100	Tetracycline, Oxacillin, Methicillin	4
S. aureus NRS108	Gentamicin, Oxacillin	8
S. aureus NRS281	Erythromycin, Spectinomycin	4
S. aureus NRS22	Vancomycin	8
S. aureus BAA-42	Methicillin, Oxacillin, Penicillin	8
S. aureus BAA-1556	Methicillin, Mupirocin	8
S. aureus BAA-1717	Methicillin, Bacitracin	8
S. aureus NRS140	Erythromycin, Spectinomycin	4
S. aureus HU25	Oxacillin	8
S. aureus WCMC039967	Ciprofloxacin, Levofloxacin	4

Supplementary Table 11. MIC values of conkatamycin (**3**) at pH 5 against antibiotic resistant *S. aureus* strains.

***S. aureus* strain**	**Antibiotic resistance**	**MIC (µg/mL)**
NRS100	Tetracycline, Oxacillin, Methicillin	0.25
NRS108	Gentamicin, Oxacillin	0.25
NRS281	Erythromycin, Spectinomycin	0.25
NRS22	Vancomycin	0.25
ATCC BAA-42	Methicillin, Oxacillin, Penicillin	0.25
ATCC BAA-1721	Hyper-virulent	0.5
NRS 146	Vancomycin	0.5
NRS140	Erythromycin, Spectinomycin	0.5
COL (Anu 189)		0.5
HAR22	Oxacillin	0.5
WCMC039967	Ciprofloxacin, Levofloxacin	0.5

Supplementary Table 12. MIC values of conkatamycin (**3**) at pH 5 against *S. aureus* in the presence of various additives. MIC does not increase in presence of key cell wall components.

**Material added to media**	**Concentration (mg/mL)**	**MIC (µg/mL)**	
		**Repeat 1**	**Repeat 2**
No additive control	N/A	0.25	0.25
Peptidoglycan	0.01	0.5	0.5
Lipoteichoic acid (G+) *S.aureus*	0.1	0.5	0.5
UDP MurNAc	0.1	0.25	0.25
N-Acetylmuramic acid	0.1	0.25	0.25
N-Acetyl-D-glucosamine	0.1	0.25	0.25
N-Acetylmuramyl-L-alanyl-D-isoglutamine hydrate	0.1	0.25	0.25
$CaCl_2$-2H2O	15 mM	0.25	0.25
ZnCl2	15 mM	0.25	0.25

Supplementary Table 13. MIC values (μg/mL) of conkatamycin (**3**) against *S. aureus* at different pHs.

	**Conkatamycin**	**Vancomycin**
pH 5.0	1	1
pH 6.0	8	1
pH 7.0	8	1

Supplementary Table 14. BGCs successfully expressed in either *S. albus* or *S. lividans*.[a]

BGC	S. albus J1074	S. lividans RedStrep 1.7	
107M20	green	green	pentamycin
109B1	green	green	pactamides
13O10	green	green	rakicidin D
34B15	green	green	lydiamycin A
56D17	green	green	prolinolexin (1)
100F3	green	green	
134I9	green	green	
140L12	green	green	
119J3	green	green	cinnamexin (2)
62J20	red	green	JBIR-100
141E17	red	green	conkatamycin (3)
81O18	green	red	bafilomycin B1
21J18	green	red	
62L12	green	red	
42E16	green	red	
49O24	green	red	
119I12	green	red	
139J16	green	red	
44L23	green	red	
94K22	green	white	antimycin
129H18	green	white	tetramycin A
96P24	green	white	cycloheximide
117M12	green	white	
5L18	green	white	
82F14	green	white	

[a]BGCs that produced clone-specific molecules are colored coded as follows: green = new peak detected by LCMS, red = no new peaks detected by LCMS, white = not successfully conjugated. New molecules characterized in this study or known natural products associated with the BGCs are indicated.

Supplementary Table 15. Comparison of current BGC mobilization methods.

	Requires ad hoc design (primers, guides, receiver vector, etc.)	All BGC classes[a]	Efficient for large NRPS/PKS	Repetitive sequence stability	Requires genome sequence	Has been used for metagenomic BGCs[b]	Economies of scale[c]
TAR[2]	yes	yes	yes	no	yes	no	no
RecET LLHR[3]	yes	yes	no	no	yes	no	no
ExoCET[4]	yes	yes	no	no	yes	no	no
CATCH[5]	yes	yes	no	yes	yes	no	no
CAPTURE[6]	yes	yes	yes	yes	yes	no	no
CAT-FISHING[7]	yes	yes	yes	yes	yes	no	no
BAC library and specific PCR screening[8,9]	yes	yes	yes	yes	yes	no	no
Cosmid or Fosmid library and screening with degenerate primers[10]	yes	no	yes	no (TAR step)	no	yes	no
CONKAT-seq[1] (cosmid pools)	yes	no	yes	no (TAR step)	no	yes	no
This work: CONKAT-seq with host-specific PACs	no	no	yes	yes	no	no	yes

[a] Methods relying on degenerate primers do not require prior sequence information but may not be suitable for some BGC classes.

[b] Metagenomic BGCs are BGCs cloned directly from environmental DNA or without requiring access to an axenic microbial culture.

[c] Economies of scale occur when increasing output leads to a lower average cost and time per BGC mobilized.

Supplementary Table 16. Assessment of capture and detection performance for NRPS or PKS BGCs based on available reference genomes.[a]

Genome	contig	BGC Start	BGC Stop	corresponding CONKAT network
Streptomyces ambofaciens ATCC 23877	NZ_CP012382	352106	409148	312
Streptomyces ambofaciens ATCC 23877	NZ_CP012382	5967338	6143205	72
Streptomyces ambofaciens ATCC 23877	NZ_CP012382	7565298	7616302	47
Streptomyces ambofaciens ATCC 23877	NZ_CP012382	7666255	7834600	47
Streptomyces anulatus YINM00001	NZ_CP086102	195788	260857	12
Streptomyces anulatus YINM00001	NZ_CP086102	287906	392629	12
Streptomyces anulatus YINM00001	NZ_CP086102	479531	531991	-
Streptomyces anulatus YINM00001	NZ_CP086102	685699	790947	-
Streptomyces anulatus YINM00001	NZ_CP086102	830794	877591	121
Streptomyces anulatus YINM00001	NZ_CP086102	1624226	1702487	145
Streptomyces anulatus YINM00001	NZ_CP086102	3934308	4072949	36
Streptomyces anulatus YINM00001	NZ_CP086102	5085934	5173228	-
Streptomyces anulatus YINM00001	NZ_CP086102	6107104	6175310	63
Streptomyces anulatus YINM00001	NZ_CP086102	7997552	8047034	40
Streptomyces anulatus YINM00001	NZ_CP086102	8099303	8191692	104
Streptomyces aureoverticillatus JCM 4347	NZ_BMSY01000002	546271	600783	-
Streptomyces aureoverticillatus JCM 4347	NZ_BMSY01000004	224768	303948	105
Streptomyces aureoverticillatus JCM 4347	NZ_BMSY01000004	453654	523986	-
Streptomyces aureoverticillatus JCM 4347	NZ_BMSY01000004	540657	728972	157
Streptomyces aureoverticillatus JCM 4347	NZ_BMSY01000009	57690	108769	13
Streptomyces aureoverticillatus JCM 4347	NZ_BMSY01000012	158142	210466	-
Streptomyces aureoverticillatus JCM 4347	NZ_BMSY01000013	0	50802	156
Streptomyces aureoverticillatus JCM 4347	NZ_BMSY01000014	87465	137827	-
Streptomyces aureoverticillatus JCM 4347	NZ_BMSY01000025	0	43024	-
Streptomyces geysiriensis JCM 4962	NZ_BMWF01000008	103339	151500	222
Streptomyces geysiriensis JCM 4962	NZ_BMWF01000008	245690	296643	270
Streptomyces geysiriensis JCM 4962	NZ_BMWF01000019	105209	175831	181
Streptomyces geysiriensis JCM 4962	NZ_BMWF01000011	0	43687	247
Streptomyces goshikiensis JCM 4640	NZ_BMVE01000003	642893	698120	-
Streptomyces goshikiensis JCM 4640	NZ_BMVE01000007	230072	467802	261
Streptomyces goshikiensis JCM 4640	NZ_BMVE01000010	10936	130266	88
Streptomyces zaomyceticus JCM 4864	NZ_BNBZ01000002	95922	185084	125
Streptomyces zaomyceticus JCM 4864	NZ_BNBZ01000002	294825	454606	-
Streptomyces zaomyceticus JCM 4864	NZ_BNBZ01000011	2	55130	-
Streptomyces tubercidicus NBRC 13090	NZ_BLIR01000001	393736	495604	107
Streptomyces tubercidicus NBRC 13090	NZ_BLIR01000003	1464867	1635943	140
Streptomyces tubercidicus NBRC 13090	NZ_BLIR01000003	1720831	1796359	-

Genome	contig	BGC Start	BGC Stop	corresponding CONKAT network
Streptomyces gougerotii JCM 4136	NZ_BMSC01000001	48346	127988	62
Streptomyces gougerotii JCM 4136	NZ_BMSC01000007	2	56563	3
Streptomyces gougerotii JCM 4136	NZ_BMSC01000007	78144	128437	206
Streptomyces gougerotii JCM 4136	NZ_BMSC01000008	76196	137468	266
Streptomyces gougerotii JCM 4136	NZ_BMSC01000009	43152	94653	5
Streptomyces gougerotii JCM 4136	NZ_BMSC01000009	110731	169212	5
Streptomyces gougerotii JCM 4136	NZ_BMSC01000019	0	45236	197
Streptomyces gougerotii JCM 4136	NZ_BMSC01000008	194003	275873	5
Streptomyces gougerotii JCM 4136	NZ_BMSC01000009	119	42635	5
Streptomyces lateritius JCM 4389	NZ_BMTO01000003	230776	297687	184
Streptomyces lateritius JCM 4389	NZ_BMTO01000014	37714	83513	102
Streptomyces pluricolorescens JCM 4602	NZ_BMUW01000007	114399	165340	172
Streptomyces pluricolorescens JCM 4602	NZ_BMUW01000009	51657	104807	149
Streptomyces pluricolorescens JCM 4602	NZ_BMUW01000009	145637	211115	149
Streptomyces longispororuber JCM 4784	NZ_BNBT01000004	22432	69273	-
Streptomyces longispororuber JCM 4784	NZ_BNBT01000015	18972	74586	112
Streptomyces longispororuber JCM 4784	NZ_BNBT01000031	0	66422	-
Streptomyces longispororuber JCM 4784	NZ_BNBT01000040	5263	59565	113
Streptomyces longispororuber JCM 4784	NZ_BNBT01000054	0	50300	-
Streptomyces longwoodensis DSM 41677	NZ_KQ948550	597710	678881	273
Streptomyces longwoodensis DSM 41677	NZ_KQ948552	290861	416086	-
Streptomyces longwoodensis DSM 41677	NZ_KQ948558	0	45570	252
Streptomyces longwoodensis DSM 41677	NZ_KQ948558	147722	210080	166
Streptomyces longwoodensis DSM 41677	NZ_KQ948565	0	71884	-
Streptomyces longwoodensis DSM 41677	NZ_KQ948566	11152	89874	105
Streptomyces massasporeus JCM 4139	NZ_BMSE01000001	450793	525529	260
Streptomyces massasporeus JCM 4139	NZ_BMSE01000033	9147	59955	-
Streptomyces bellus strain JCM 4292	NZ_BMSO01000023	116910	177810	28
Streptomyces bellus strain JCM 4292	NZ_BMSO01000034	0	61040	167
Streptomyces bellus strain JCM 4292	NZ_BMSO01000018	156434	207372	70
Streptomyces badius JCM 4350	NZ_BMSZ01000003	1823	66122	-
Streptomyces badius JCM 4350	NZ_BMSZ01000010	75128	126099	44
Streptomyces badius JCM 4350	NZ_BMSZ01000013	128530	185664	-
Streptomyces badius JCM 4350	NZ_BMSZ01000025	8942	66291	64

total BGCs : 71 **total detected : 51**

[a]NRPS or PKS BGCs with more than 2 domains detected by antismash V6.01 on contigs >40 kbps associated with 15 sequenced strains included in the clone library

Supplementary Table 17. Comparison of the scalability of different BGC cloning methods.*

**Direct cloning**[2–7]	Genomic sequencing ≈1 week (for n)	-Capture vector preparation -Genomic digestion/ligation/transformation ≈1 week (for each)	PCR screening ≈1 day (for each)	n=1 BGC ≈2 week	n=100 BGCs ≈1 year	n=1000 BGCs ≈10 years
**BAC/PAC library and specific PCR screening**[8,9]	X	Library construction ≈1 weeks (for each)	Multidimensional pooling and PCR screening ≈1 week (for each)	n=1 BGC ≈2 week	n=100 BGCs ≈4 years	n=1000 BGCs ≈40 years
**CONKAT-seq with host-specific PACs**	X	Large library construction ≈1-3 weeks (for n)	Multidimensional pooling, PCR and sequencing ≈2 week (for n)	n=1 BGC ≈1-2 months	n=100 BGCs ≈1-2 months	n=1000 BGCs ≈1-2 months

*for a process run in series (parallelization of certain steps could shorten required time)

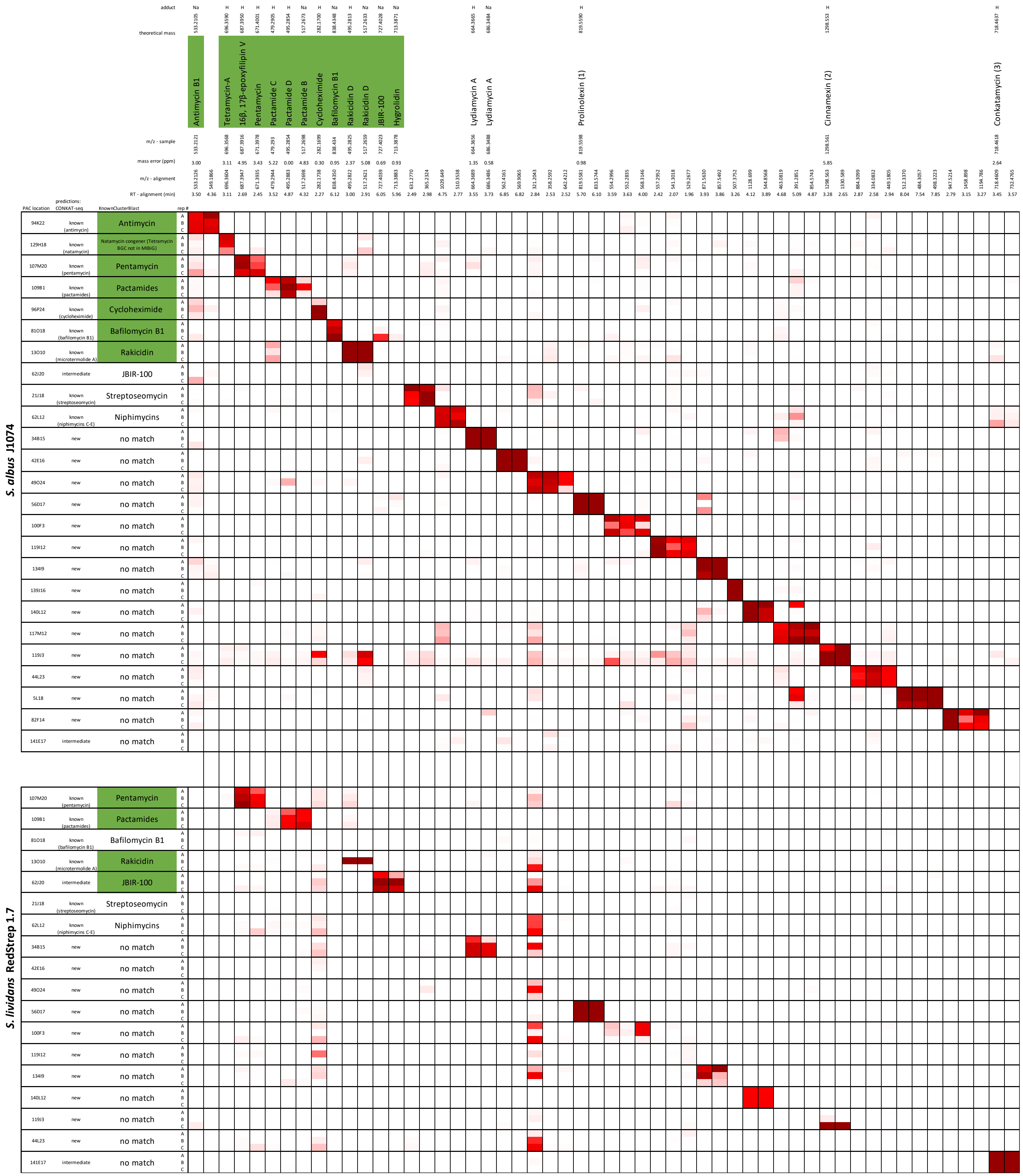

Supplementary Figure 1. Representative differential features detected by untargeted metabolomics analysis.

BGC-specific mass features were identified through an all-versus-all comparison for each host. 69 BGCs (13 known, 56 unknown) were screened in *S. albus* J1074 and 23 of them (9 known, 14 unknown) generated new specific features in multiple replicates (n ≥ 3). 56 BGCs (11 known, 45 unknown) were screened in *S. lividans* RedStrep 1.7 and 11 of them (4 known, 7 unknown) generated BGC-specific features. Chromatograms of either hosts harboring BGCs which generated specific features in triplicate in at least one of the hosts were aligned in a single matrix and gap-filled (10ppm tolerance, 30 seconds retention time tolerance). Between 1 and 3 representative features were selected for each BGC to be part of a heatmap visualization (dark red indicates high peak area values). For strains harboring a known BGCs in which a close match (<10ppm error) to the reported product (or close congener) could be found, the name of the predicted and reported products are highlighted in green. Source data are provided as a Source Data file.

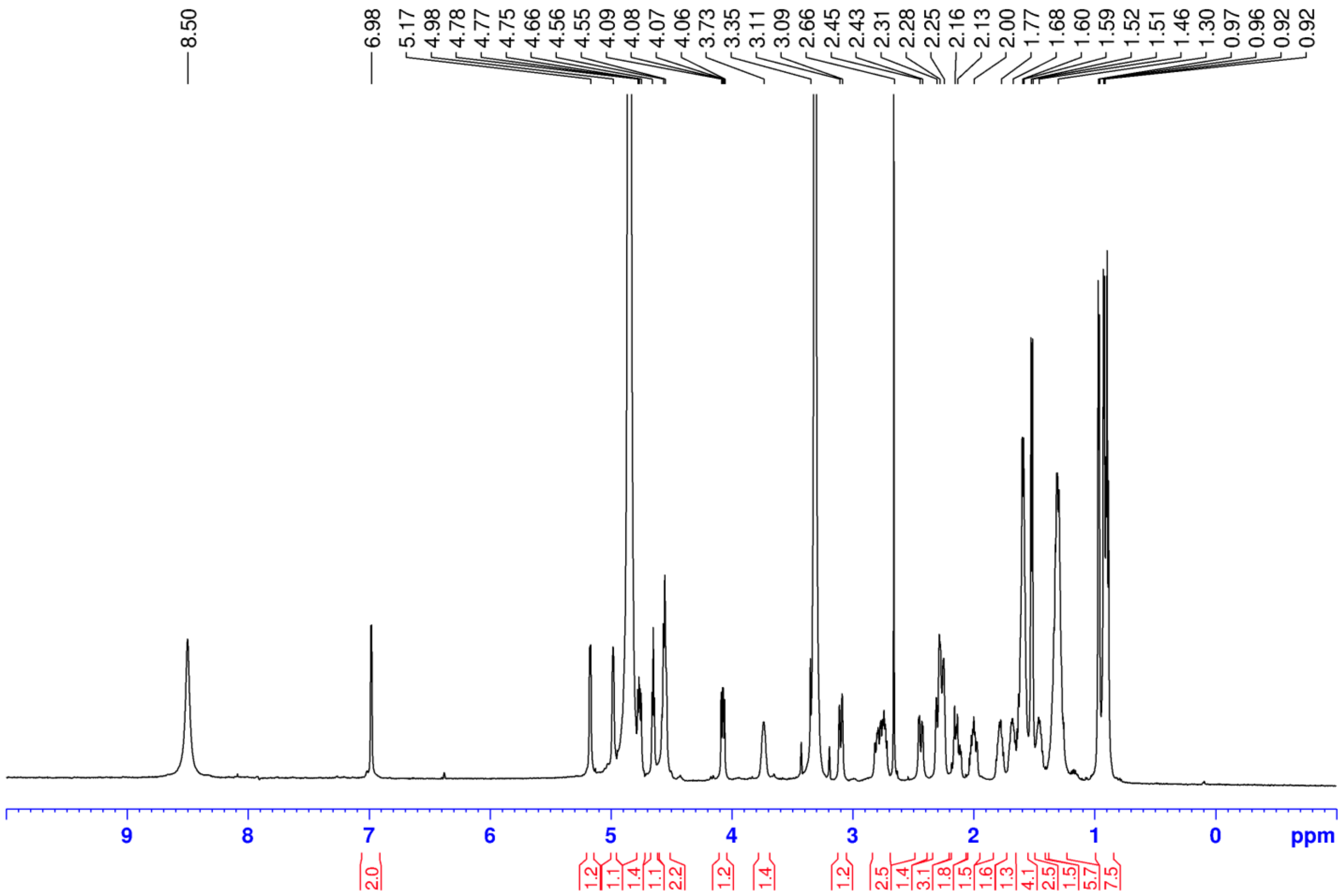

Supplementary Figure 2. ^{1}H NMR spectrum of lydiamycin A in CD_3OD. Lydiamycin A signals were acquired at 600 MHz.

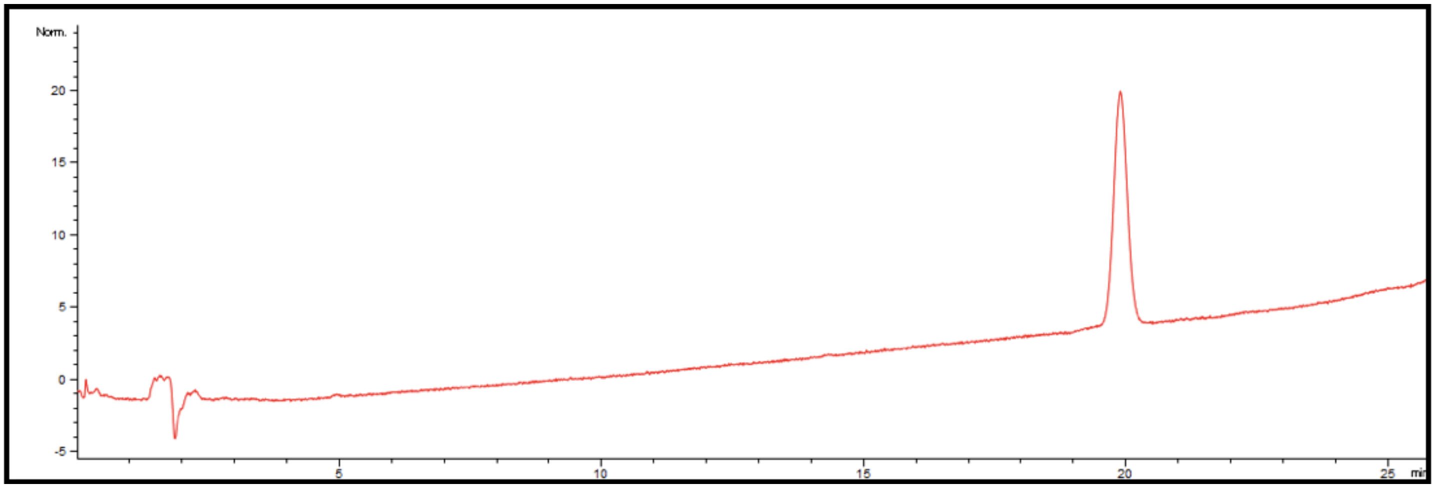

Supplementary Figure 3. Analytical HPLC-DAD trace (254 nm) for purified prolinolexin (1). We suspect that this peak represents in fact a mixture of two isomers due to the *cis-trans* isomerization of the Pro-OH amide bond.

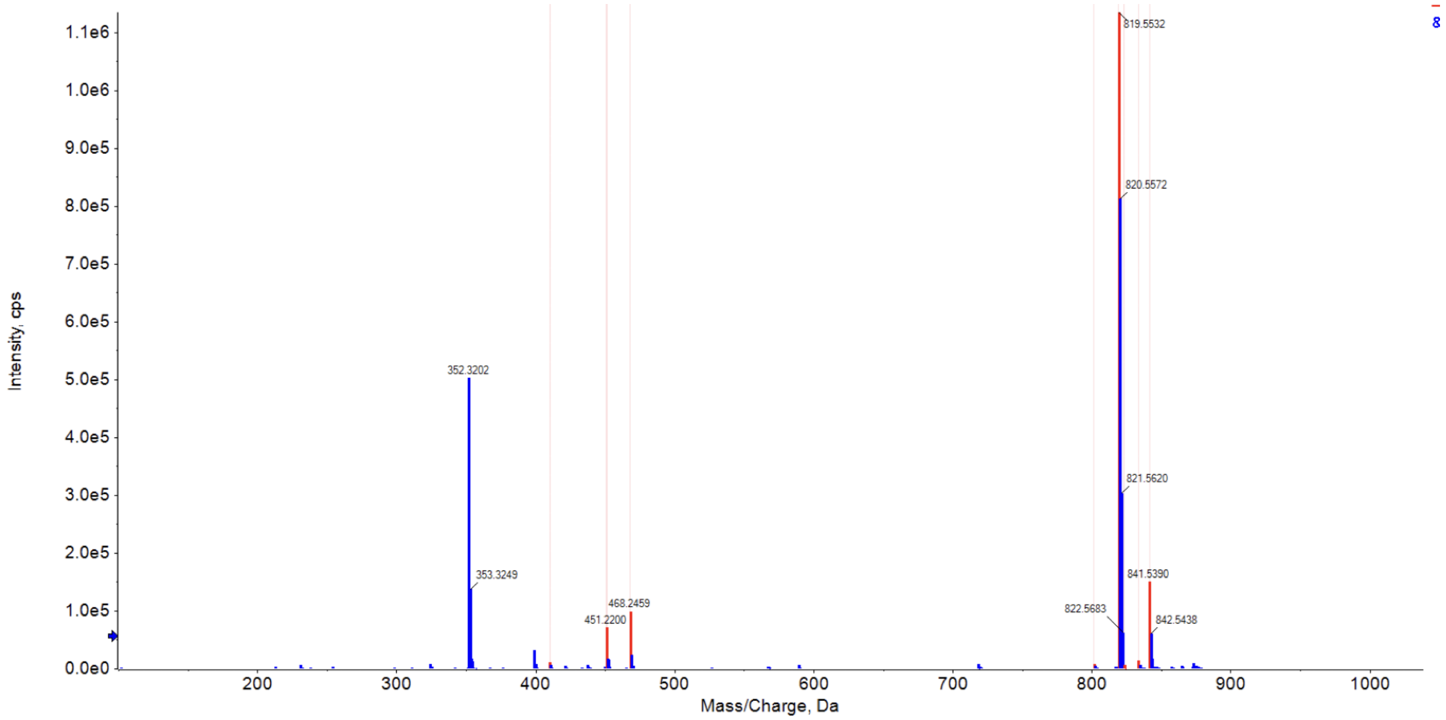

Supplementary Figure 4. ESI+ high-resolution mass spectrum of prolinolexin (**1**). Prolinolexin was determined to have a molecular formula of $C_{43}H_{74}N_6O_9$ based on *m/z* 819.5532, which represents its protonated adduct (calculated mass for $C_{43}H_{75}N_6O_9^+$ = 819.5590, Δ = 7.1 ppm).

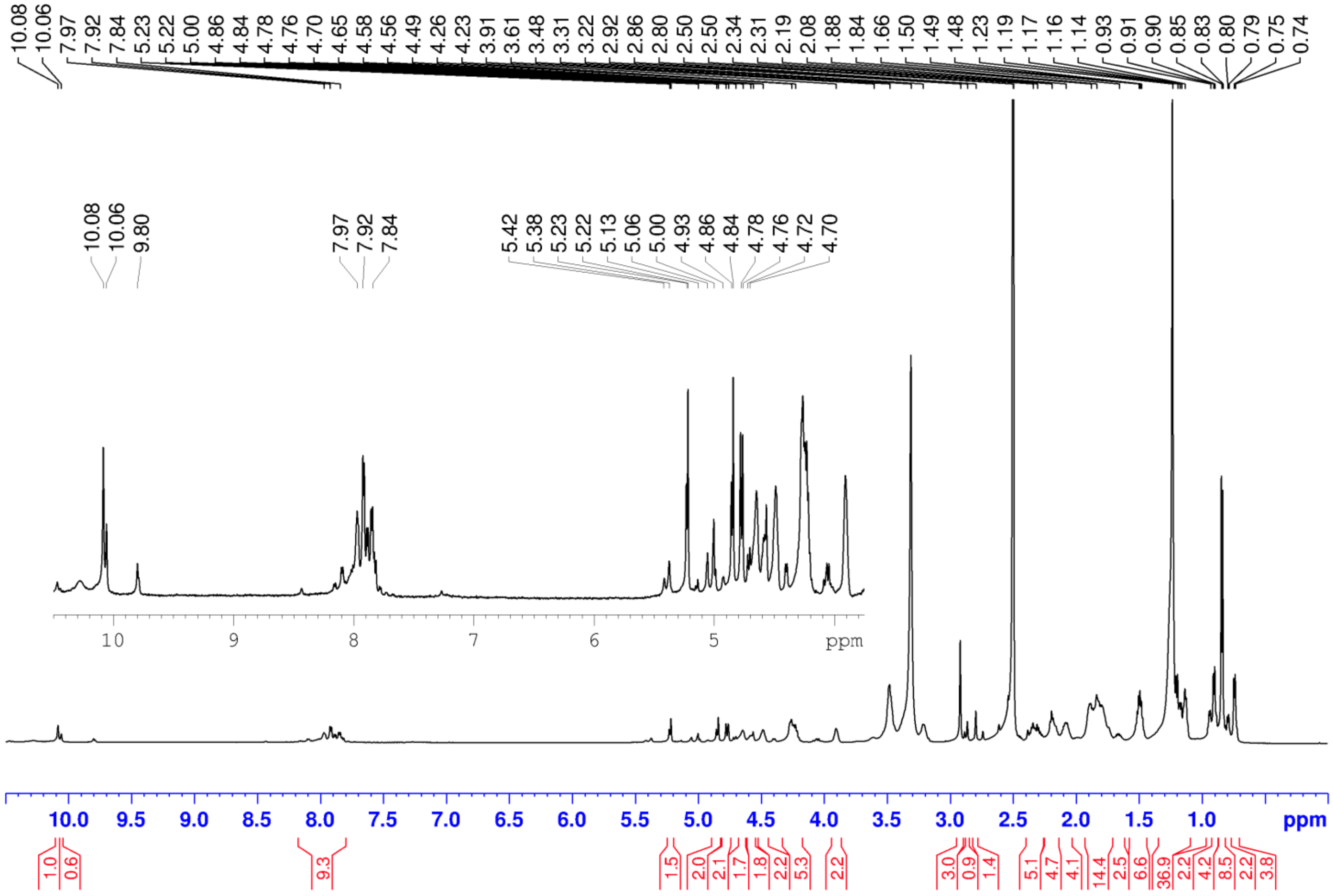

Supplementary Figure 5. ^{1}H NMR spectrum of **1** in DMSO-d_6. Prolinolexin signals were acquired at 600 MHz.

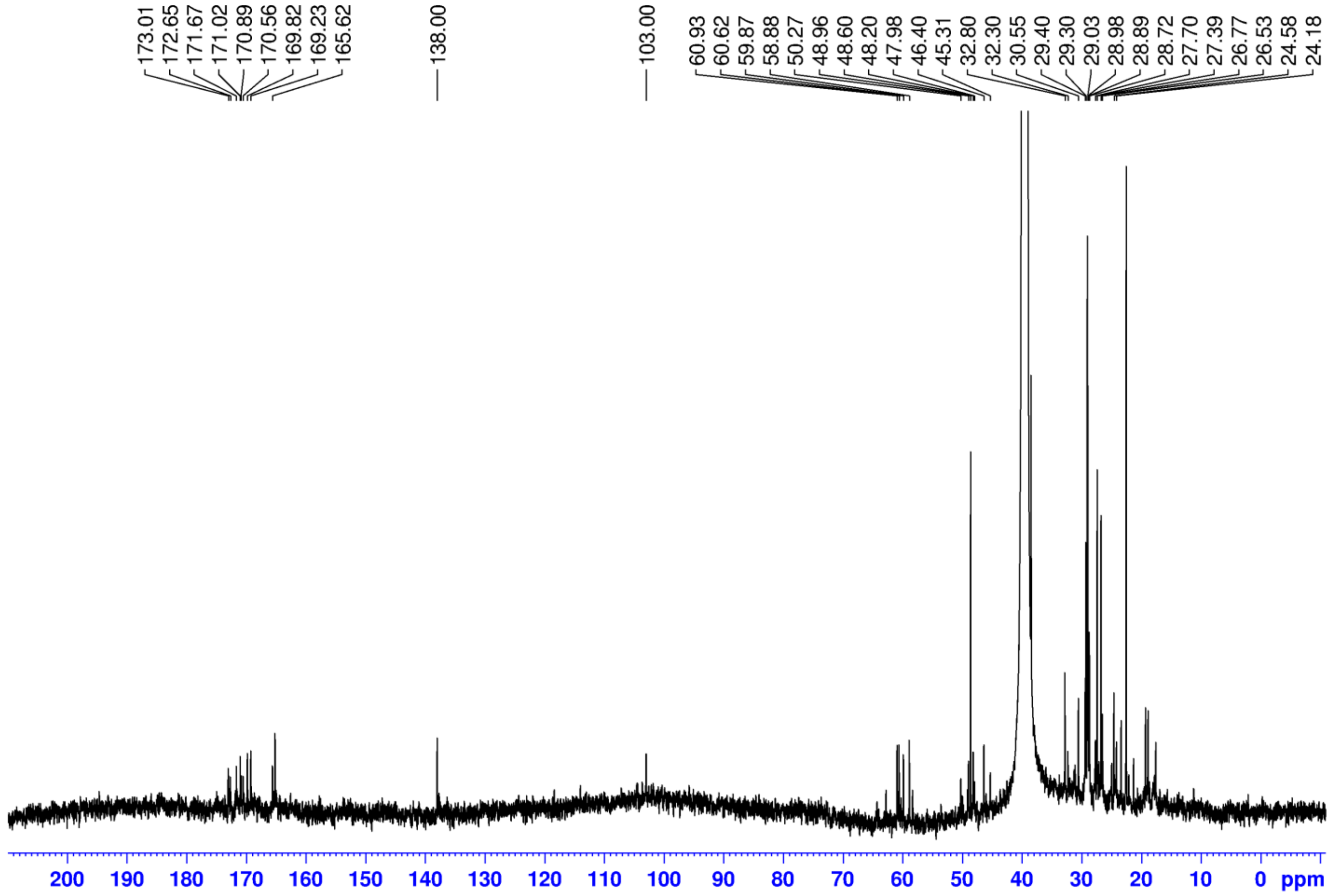

Supplementary Figure 6. ^{13}C NMR spectrum of **1** in DMSO-d_6. Prolinolexin signals were acquired at 151 MHz.

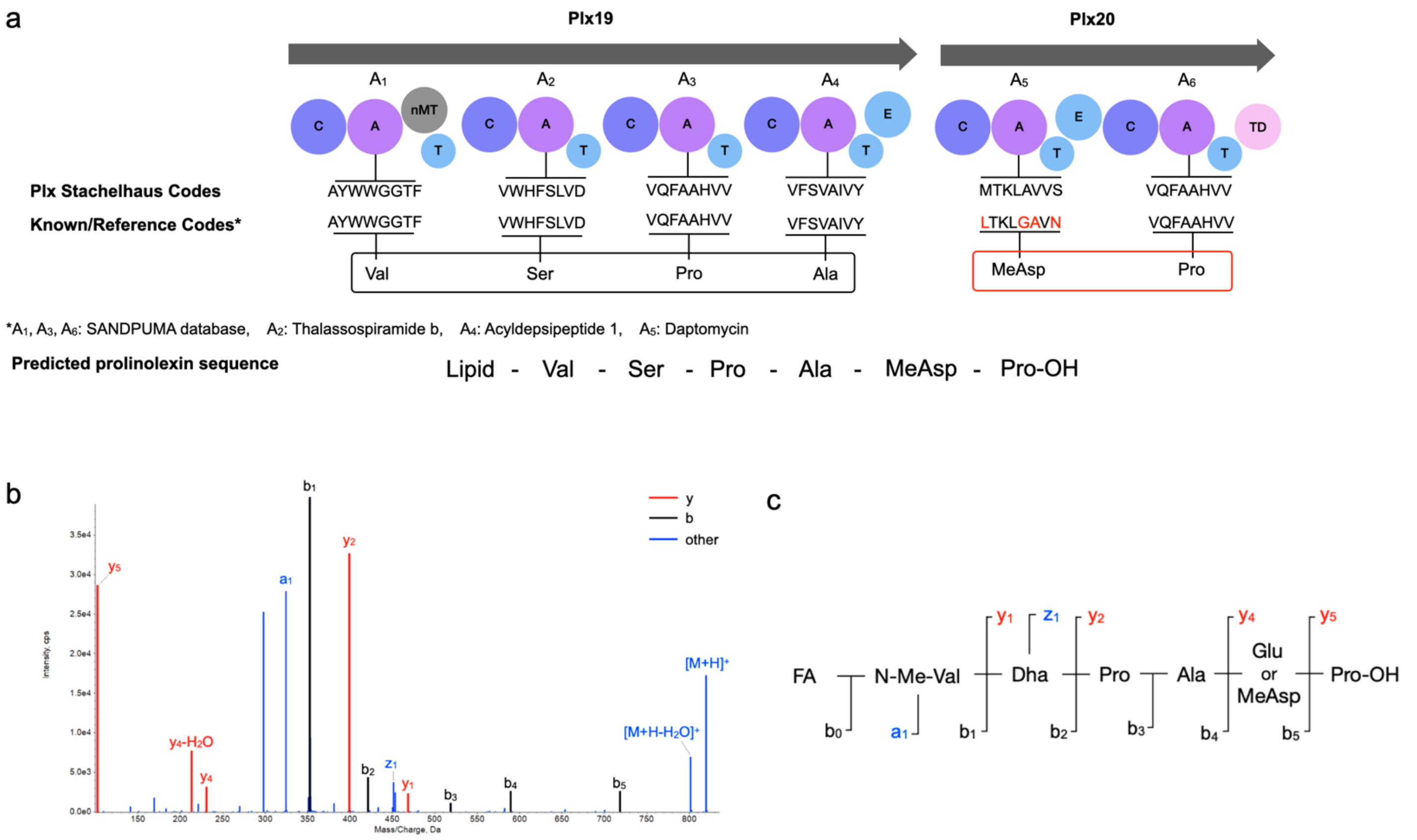

Supplementary Figure 7. Determination of the peptide sequence of (**1**). (**a**) Adenylation (A) domain analysis of the *plx* gene cluster to generate a predicted NRPS product of *plx19-20.* (**b**) MS2 spectrum of prolinolexin (*m/z* 819.5590) and (**c**) its peptide sequence, both annotated with assigned prolinolexin fragments.

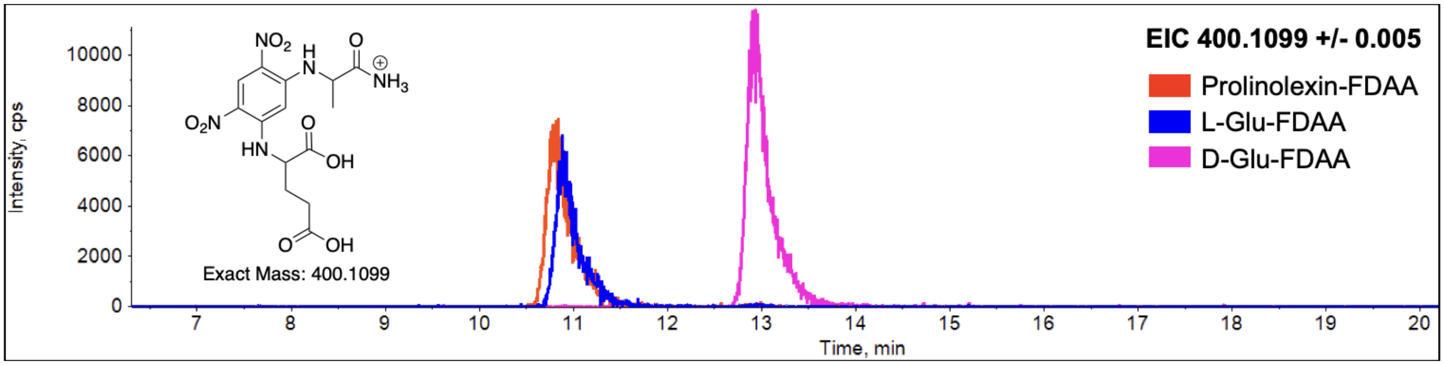

Supplementary Figure 8. Marfey's analysis of prolinolexin (**1**) to establish the presence of Glu in the peptide. Extracted ion chromatograms (EICs) of L-Glu, D-Glu and hydrolyzed **1** individually deriviatized with 1-fluoro-2-4-dinitrophenyl-5-L-alaninamide (L-FDAA).

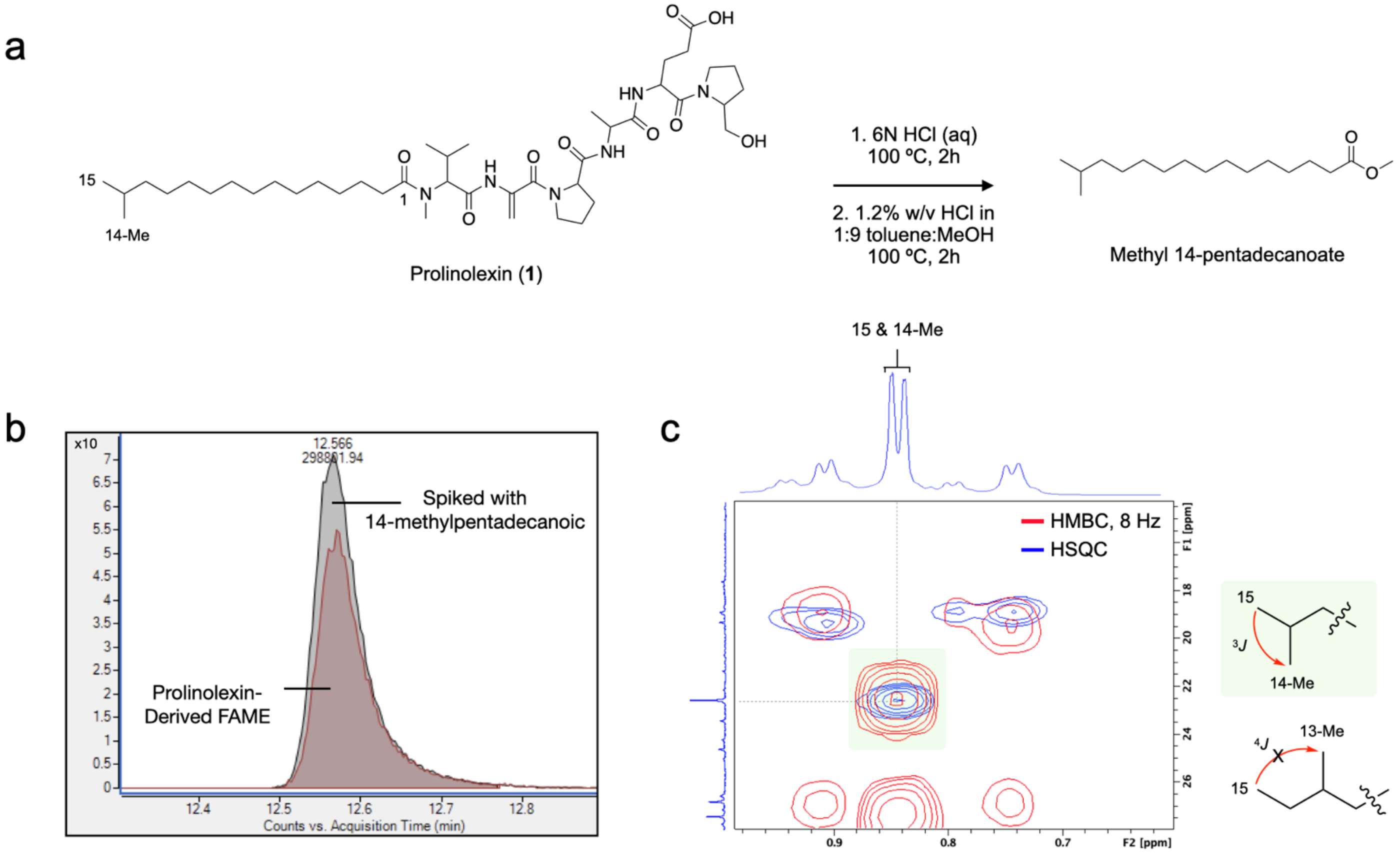

Supplementary Figure 9. Characterization of 14-methylpentadecanoyl moiety in prolinolexin. (a) Preparation of a fatty acid methyl ester (FAME) from prolinolexin. (b) GC-qTOF comparison of the prolinolexin-derived FAME to three isobaric FAME standards. (c) 2D NMR data supporting the presence of an *iso*-fatty acyl tail appended to **1**.

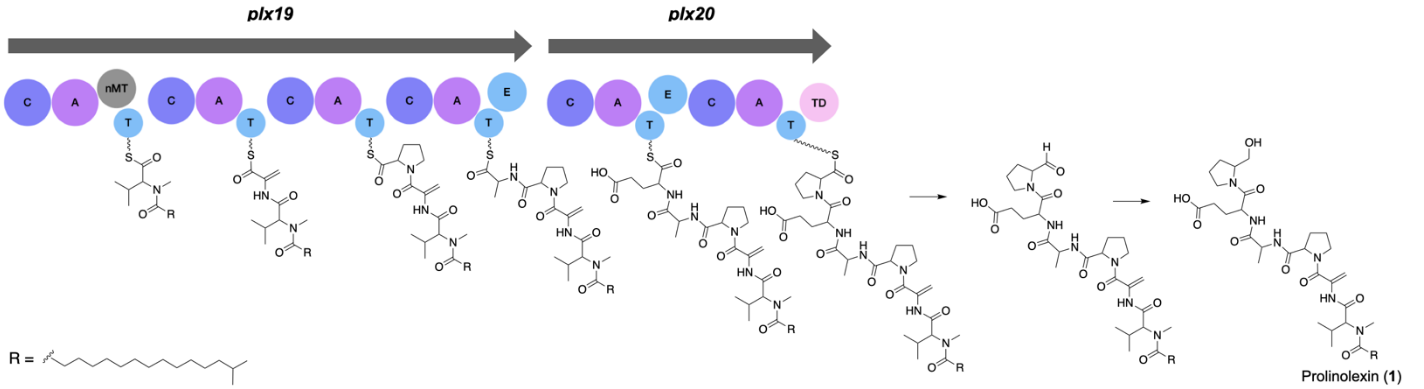

Supplementary Figure 10. Proposed NRPS assembly line biosynthesis of prolinolexin (**1**). The first and last modules in the NRPS assembly line were assigned based on the presence of a C_{START} domain in Plx19 and a thioreductase domain in Plx20.

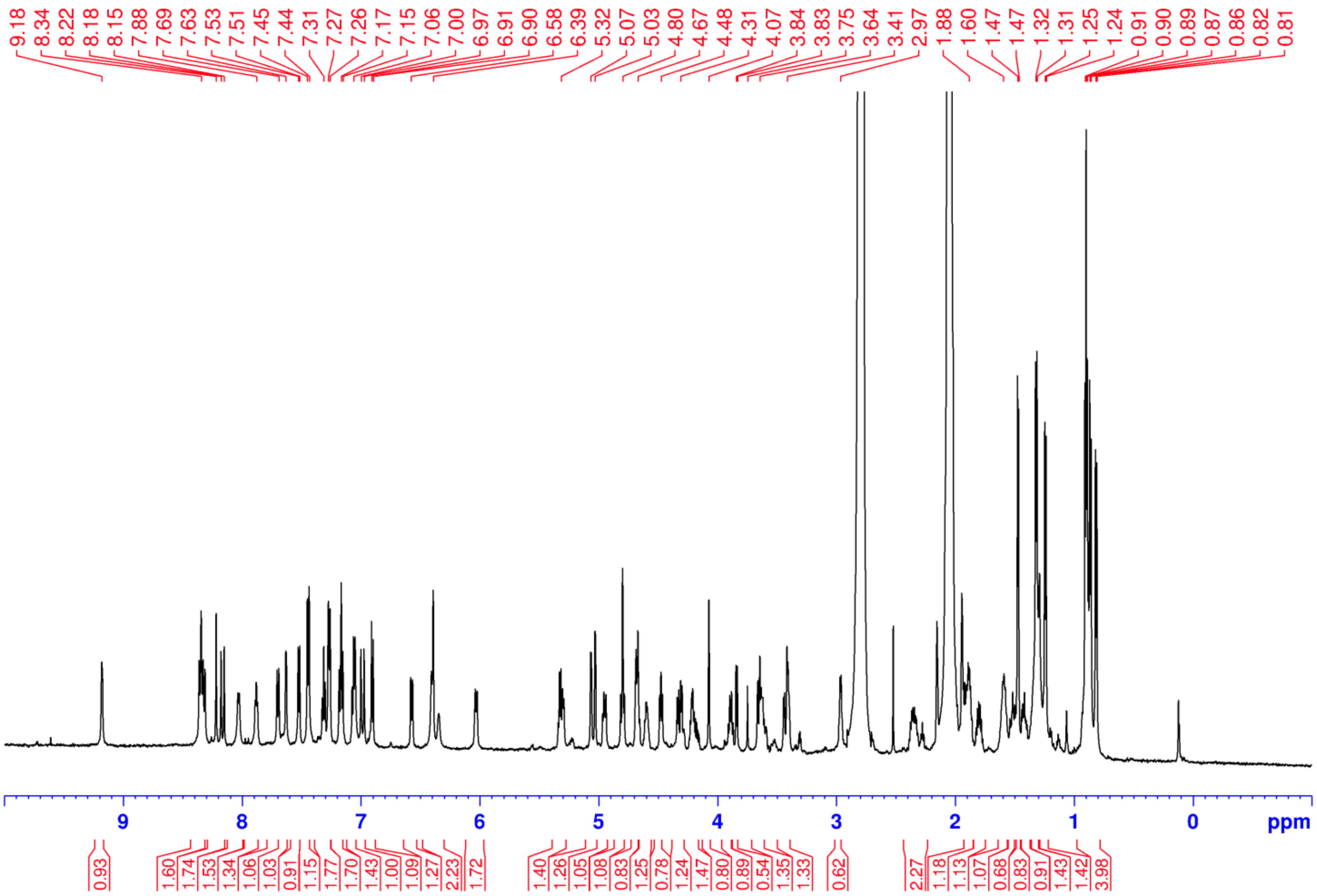

Supplementary Figure 11. ^{1}H NMR spectrum of **2** in CD_3OD. Cinnamexin signals were acquired at 600 MHz.

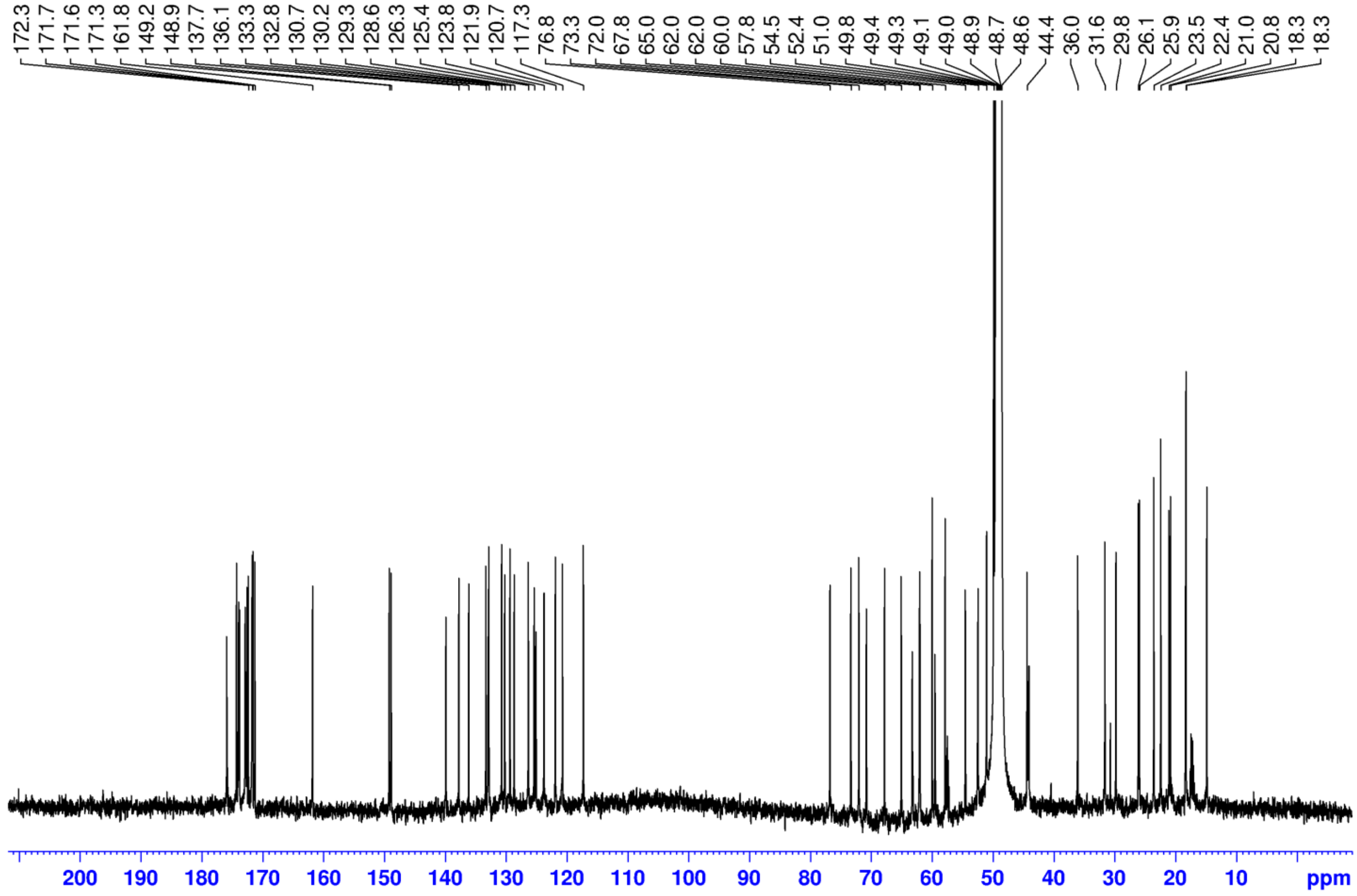

Supplementary Figure 12. ^{13}C NMR spectrum of **2** in CD_3OD. Cinnamexin signals were acquired at 151 MHz.

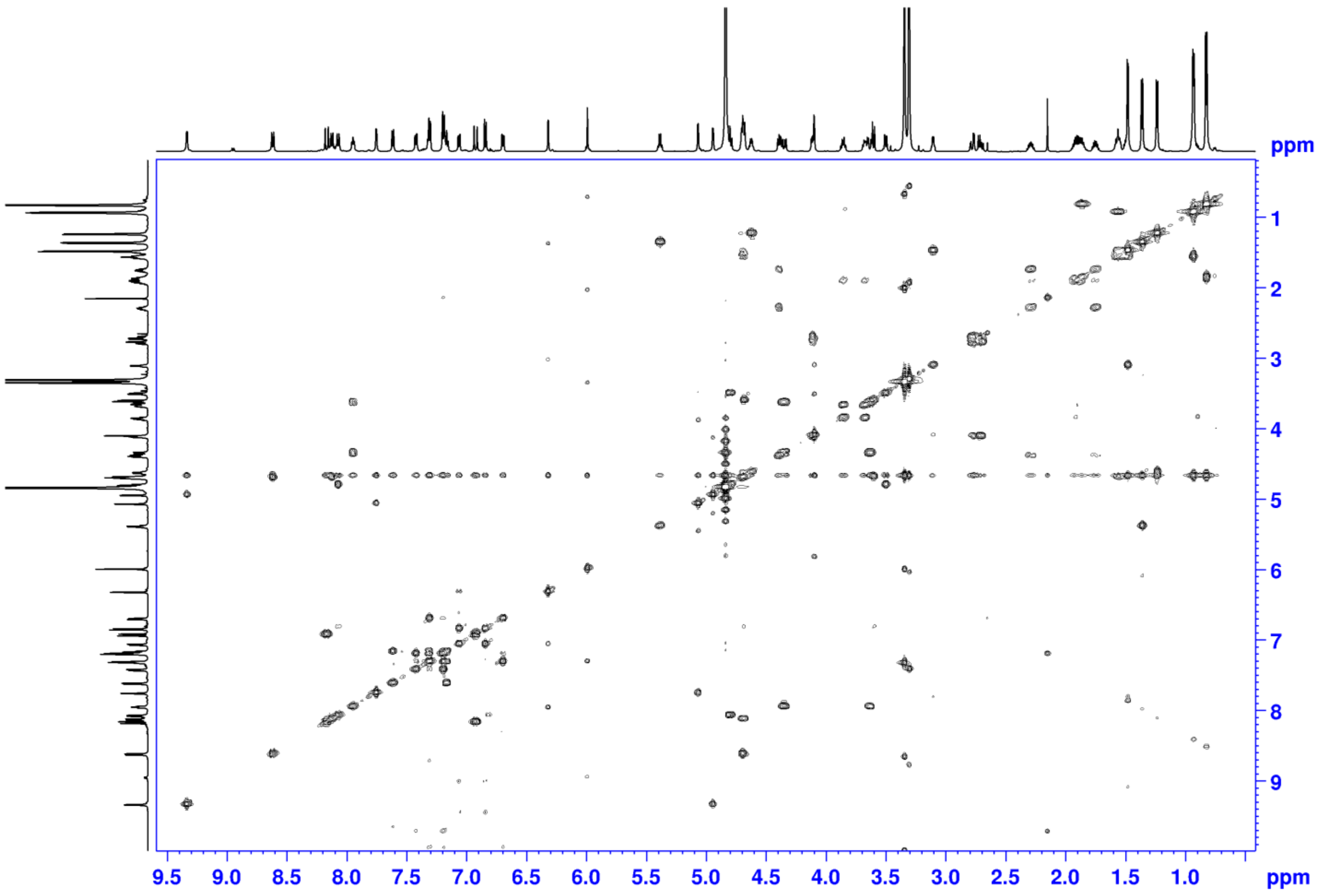

Supplementary Figure 13. COSY NMR spectrum of **2** in CD_3OD. Cinnamexin signals were acquired at 600 MHz.

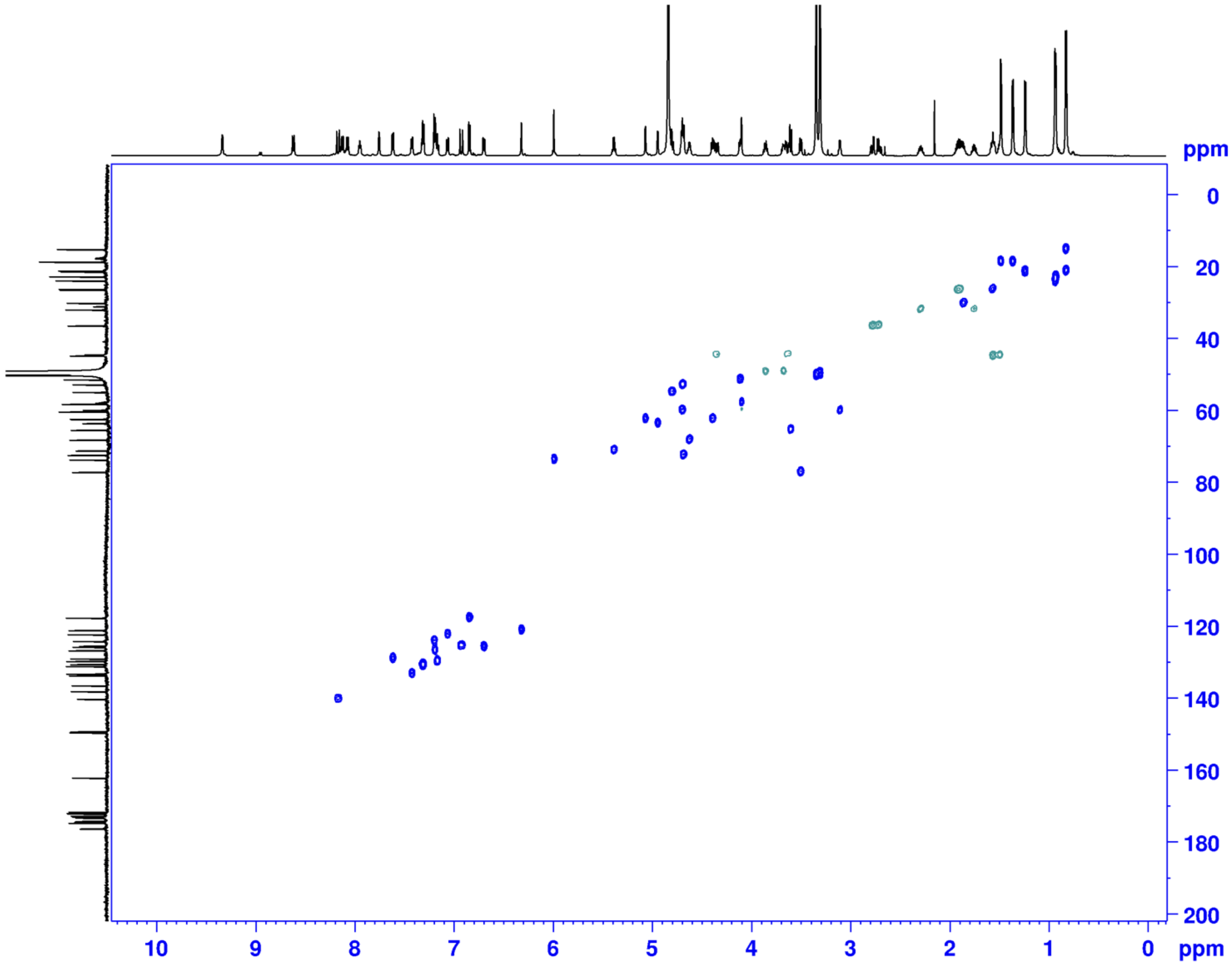

Supplementary Figure 14. Multiplicity-edited HSQC NMR spectrum of **2** in CD_3OD (^{1}H: 600MHz, ^{13}C:151 MHz). Blue contours correspond to methyls and methines, and teal contours correspond to methylenes.

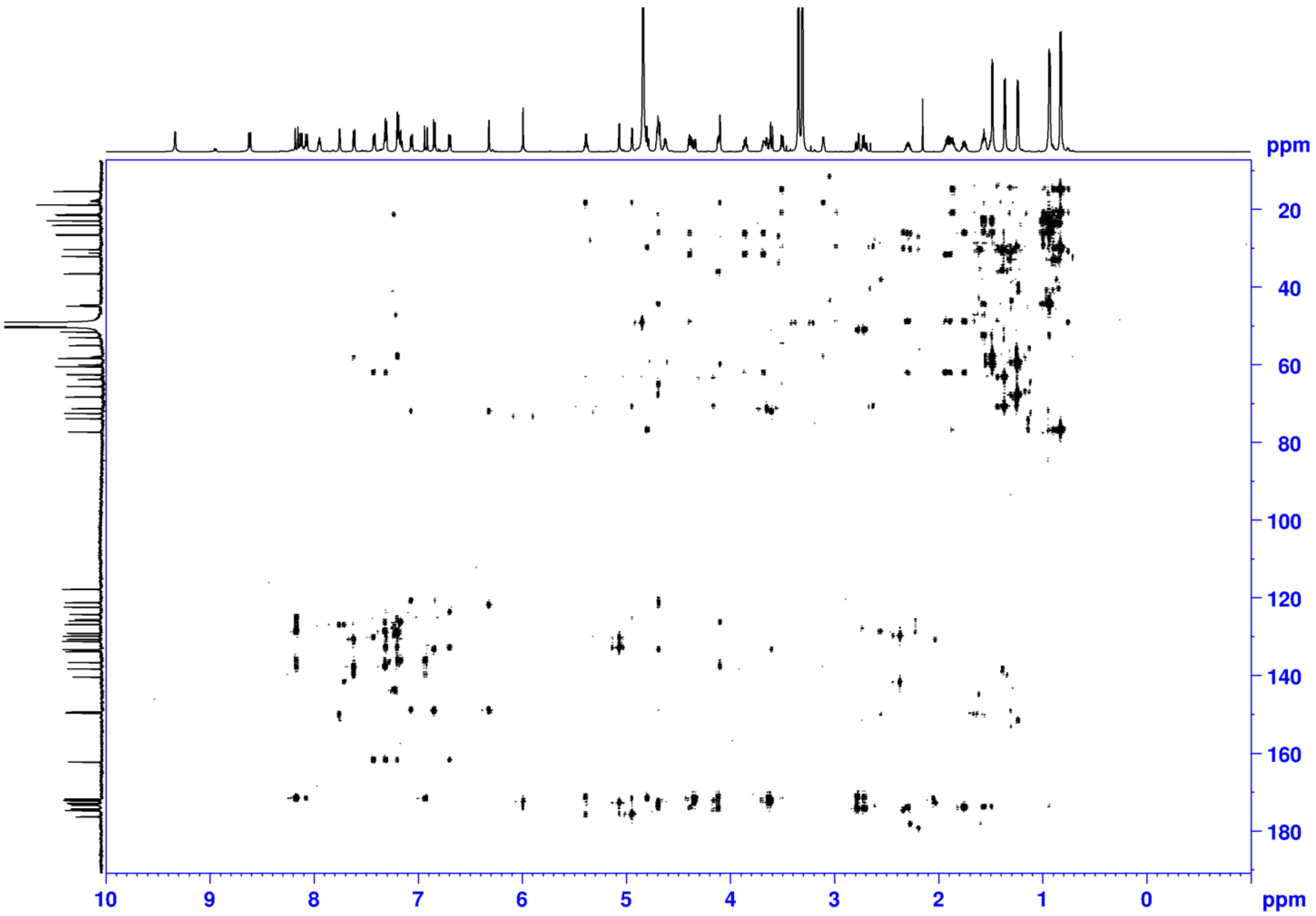

Supplementary Figure 15. HMBC NMR spectrum of **2** in CD_3OD. ^{1}H signals were acquired at 800MHz. ^{13}C signals were acquired at 201 MHz.

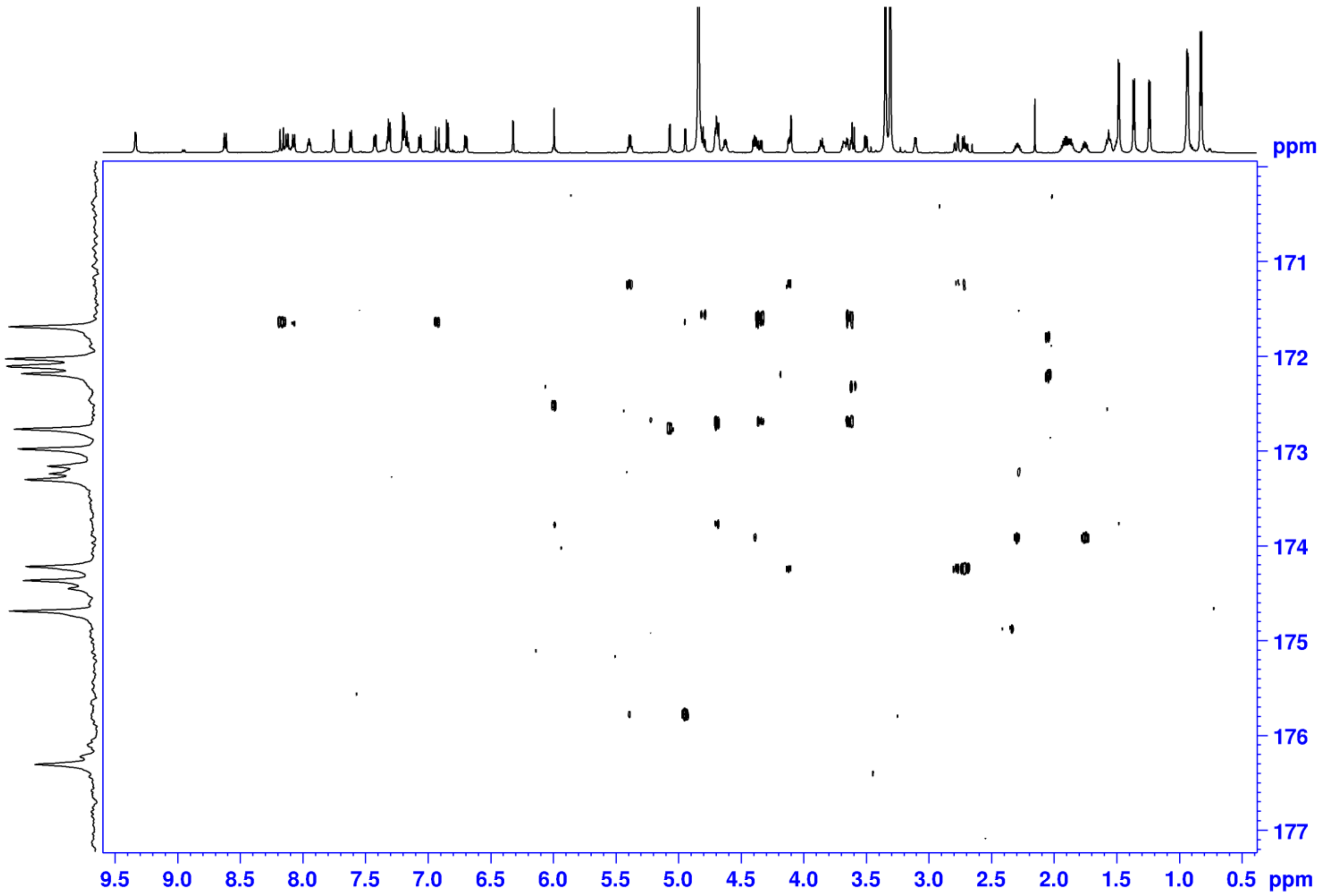

Supplementary Figure 16. Band-selective CT-HMBC (pulse program ‘shmbcctetgpl2nd’) of **2** in CD_3OD. ^{1}H signals were acquired at: 800MHz. ^{13}C signals were acquired at 201 MHz.

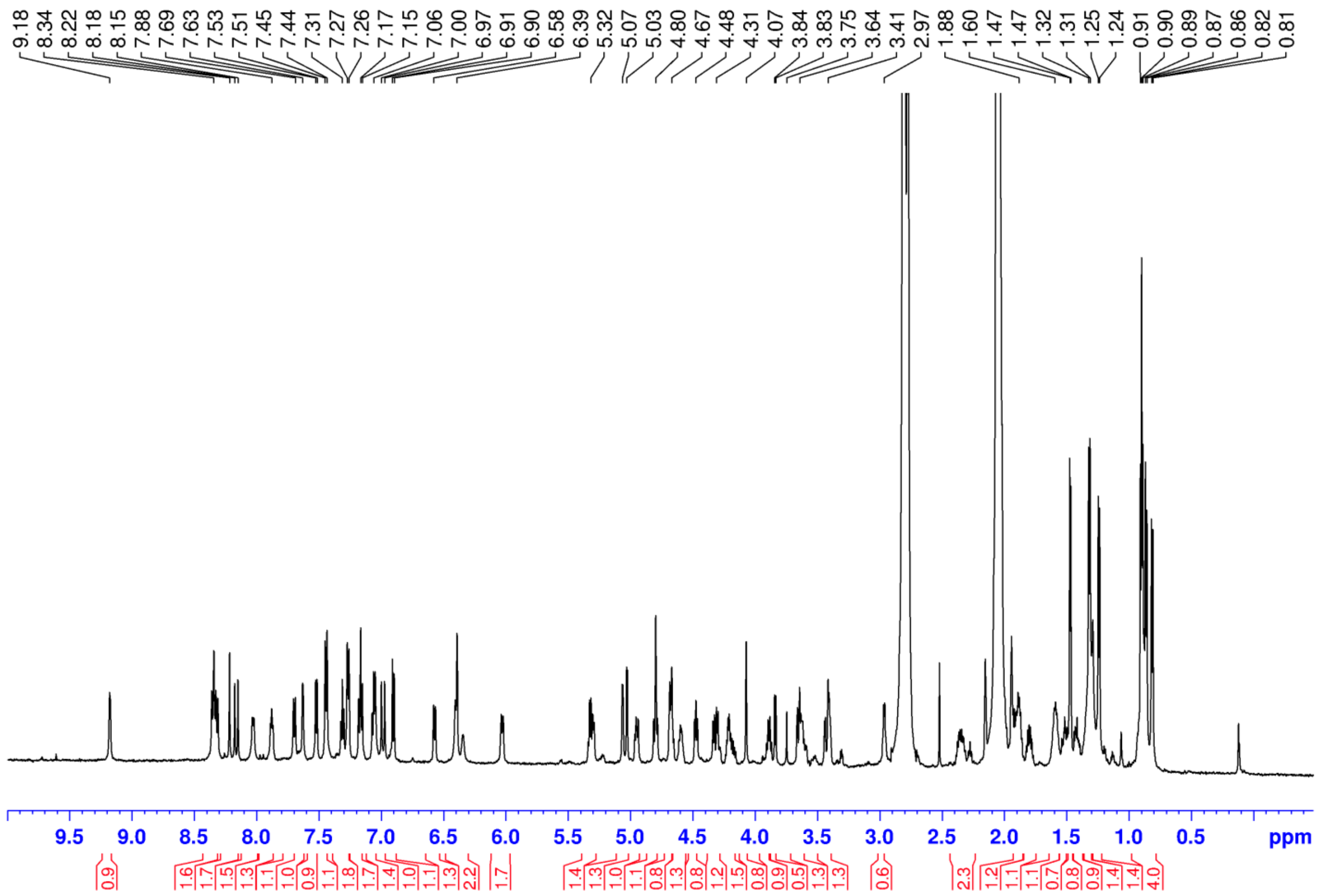

Supplementary Figure 17. ^{1}H NMR spectrum of **2** in acetone-d_6. Cinnamexin signals were acquired at 600 MHz.

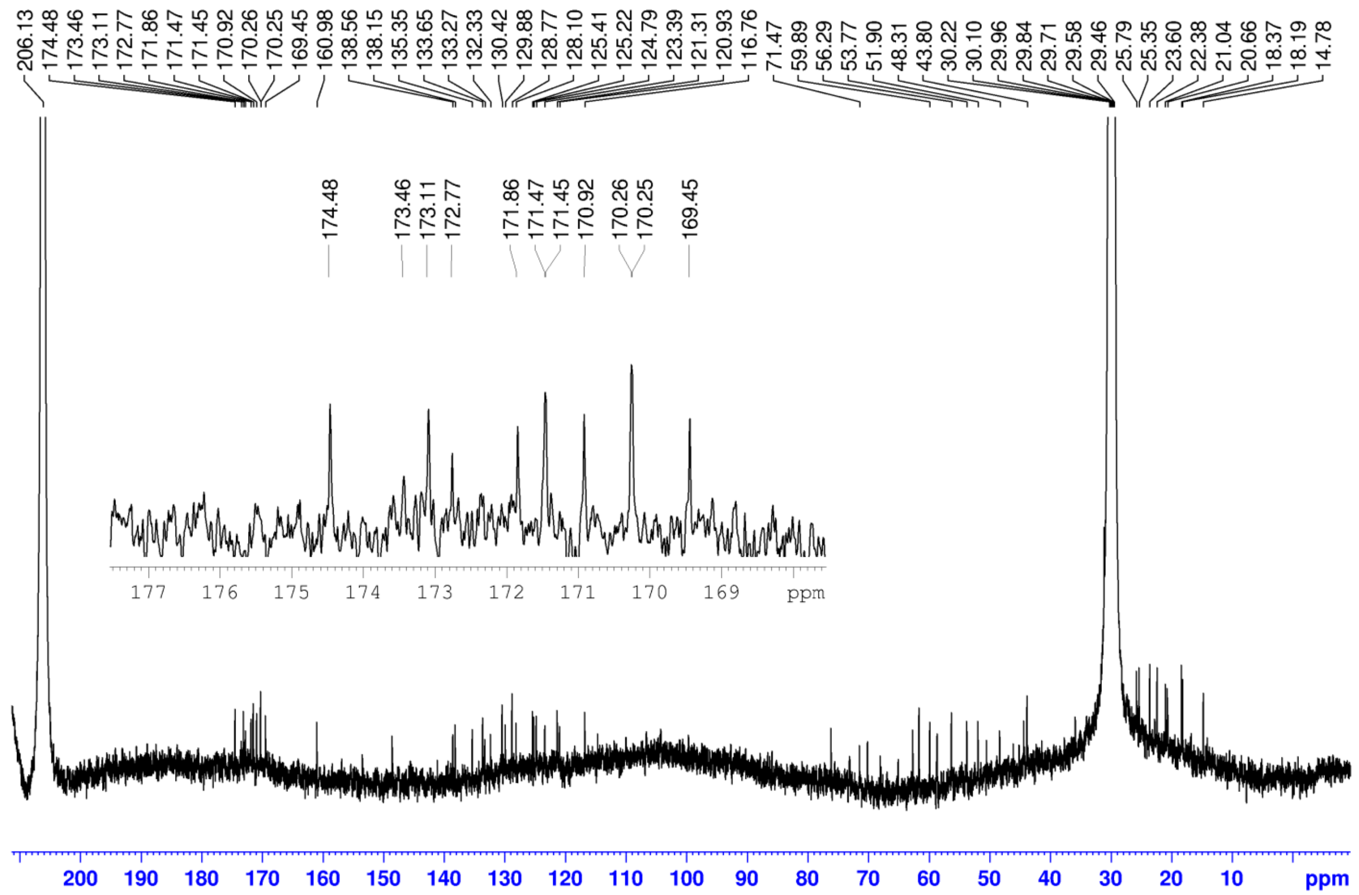

Supplementary Figure 18. ^{13}C NMR spectrum of **2** in acetone-d_6. Cinnamexin signals were acquired at 151 MHz.

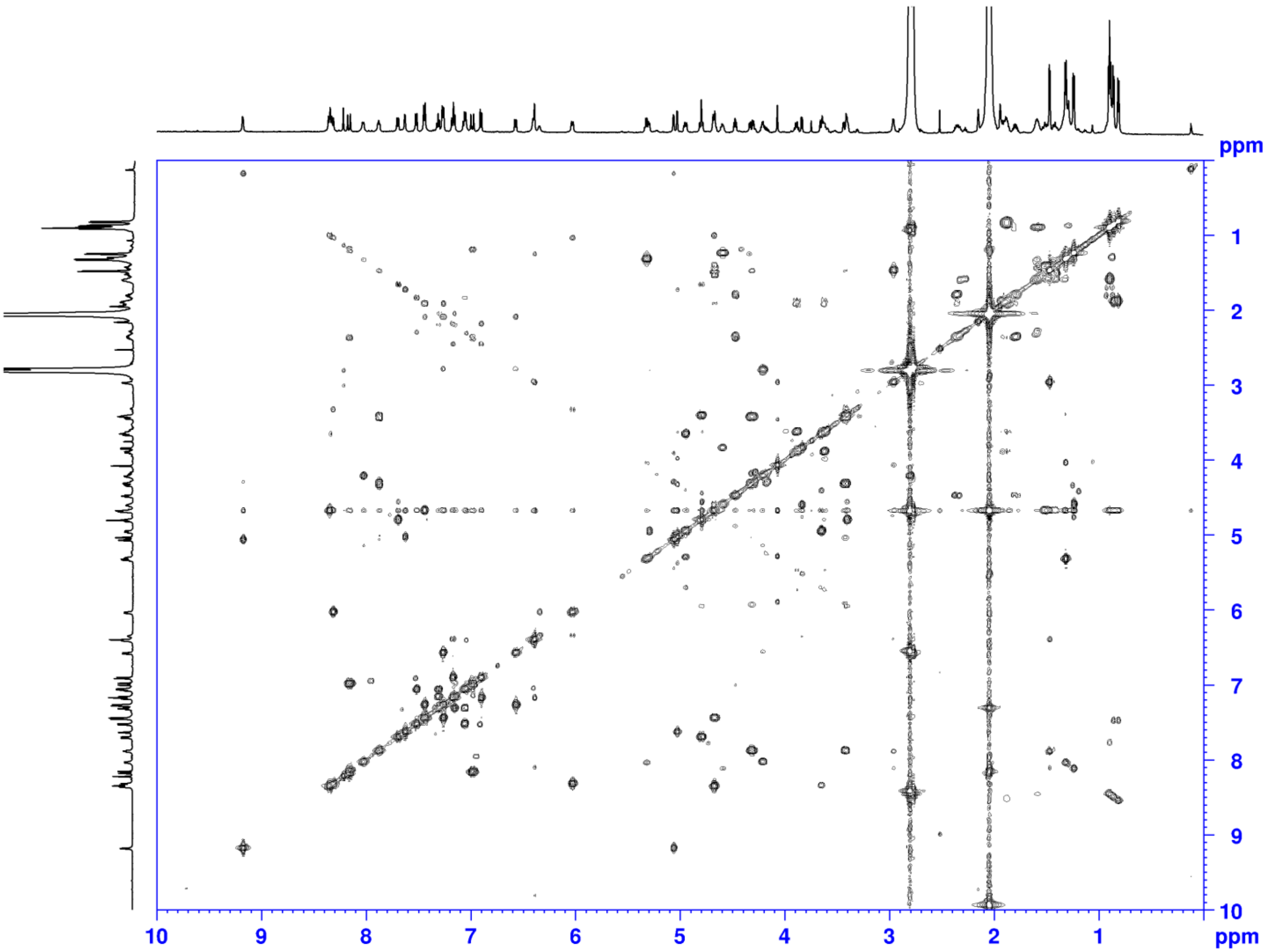

Supplementary Figure 19. COSY NMR spectrum of **2** in acetone-d_6. Cinnamexin signals were acquired at 600 MHz.

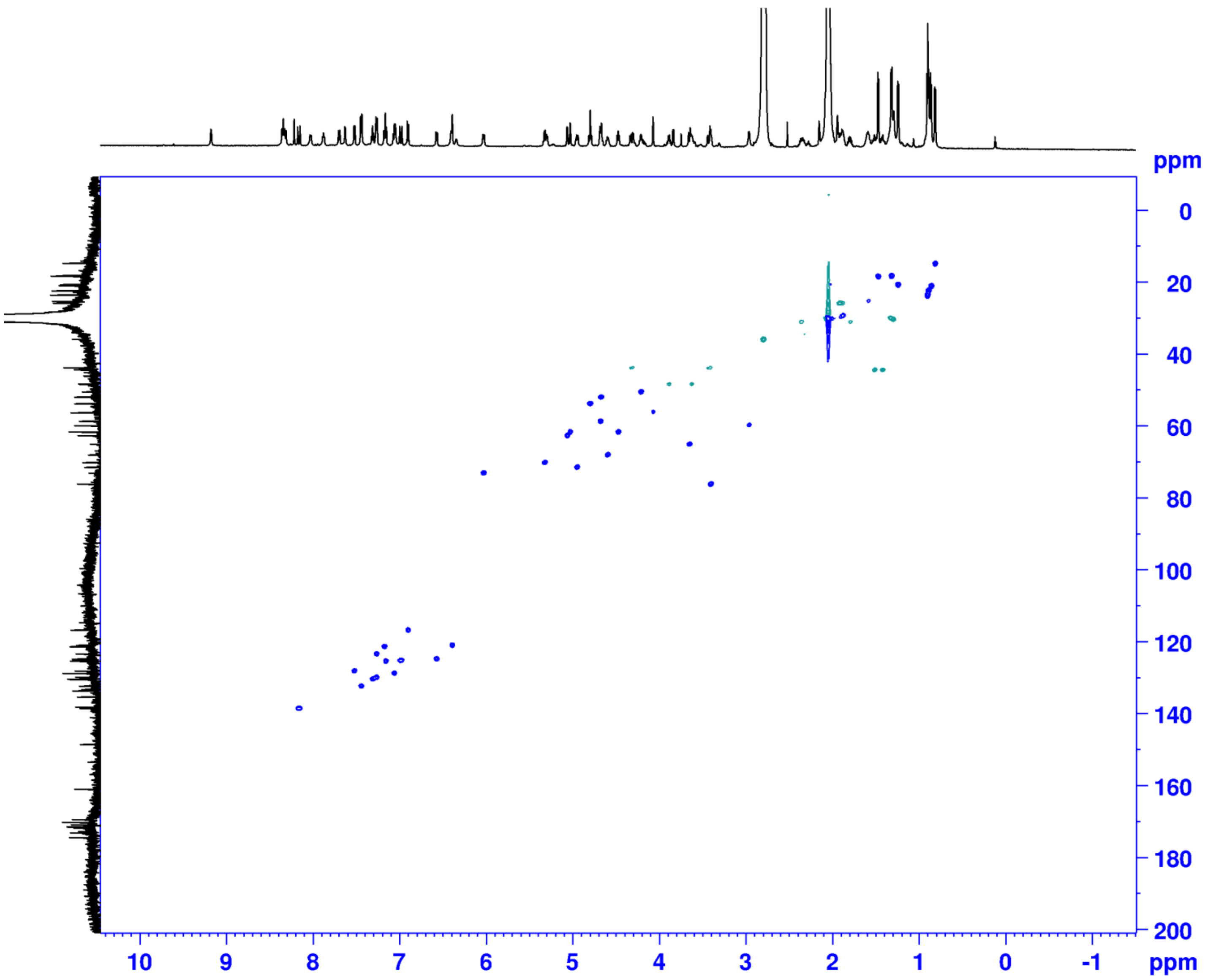

Supplementary Figure 20. Multiplicity-edited HSQC NMR spectrum of **1** in acetone-d_6 (^{1}H: 600MHz, ^{13}C:151 MHz). Blue contours correspond to methyls and methines, and teal contours correspond to methylenes.

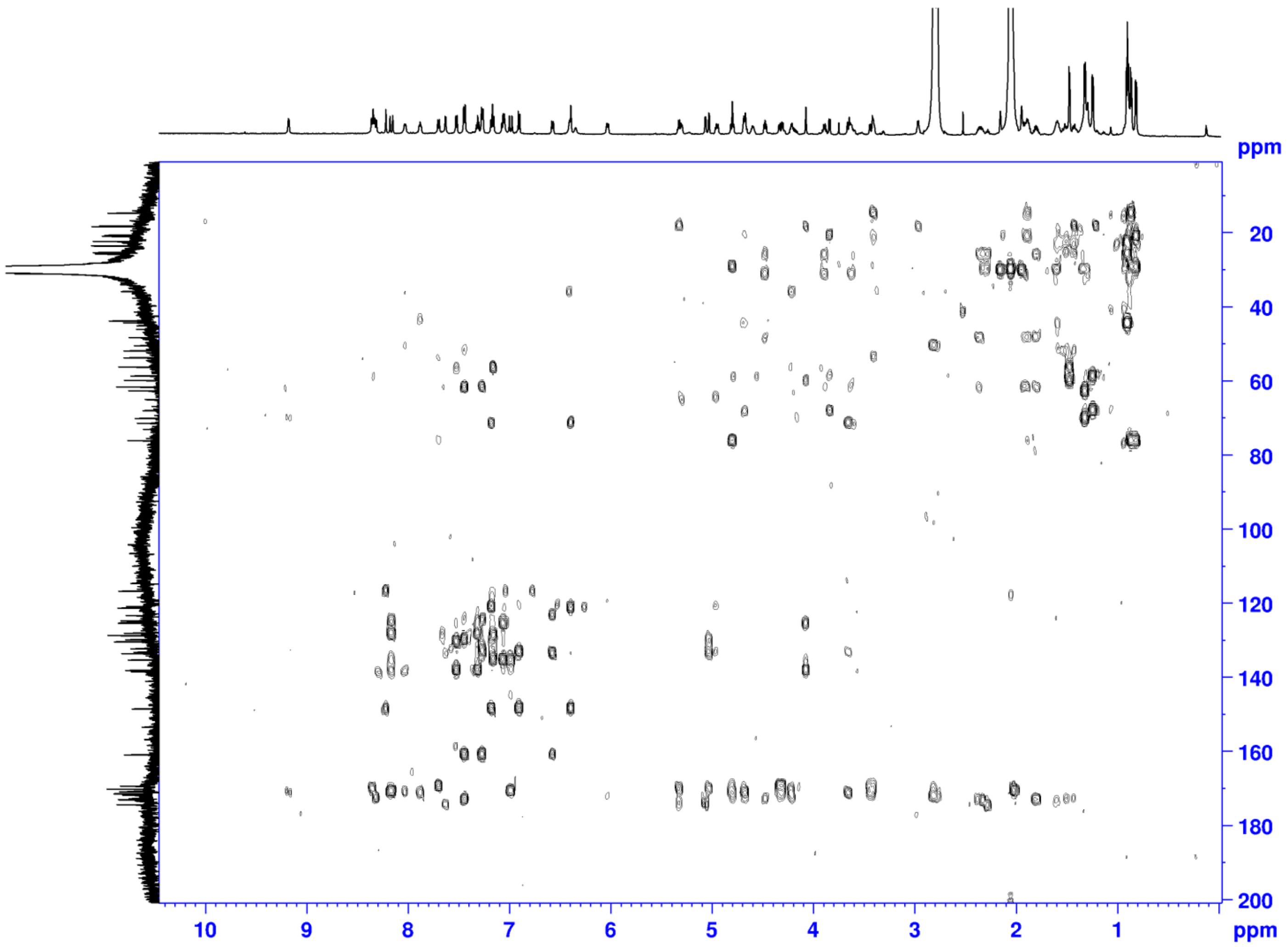

Supplementary Figure 21. HMBC NMR spectrum of **2** in acetone-d_6. ^{1}H signals were acquired at 600 MHz. ^{13}C signals were acquired at 151 MHz.

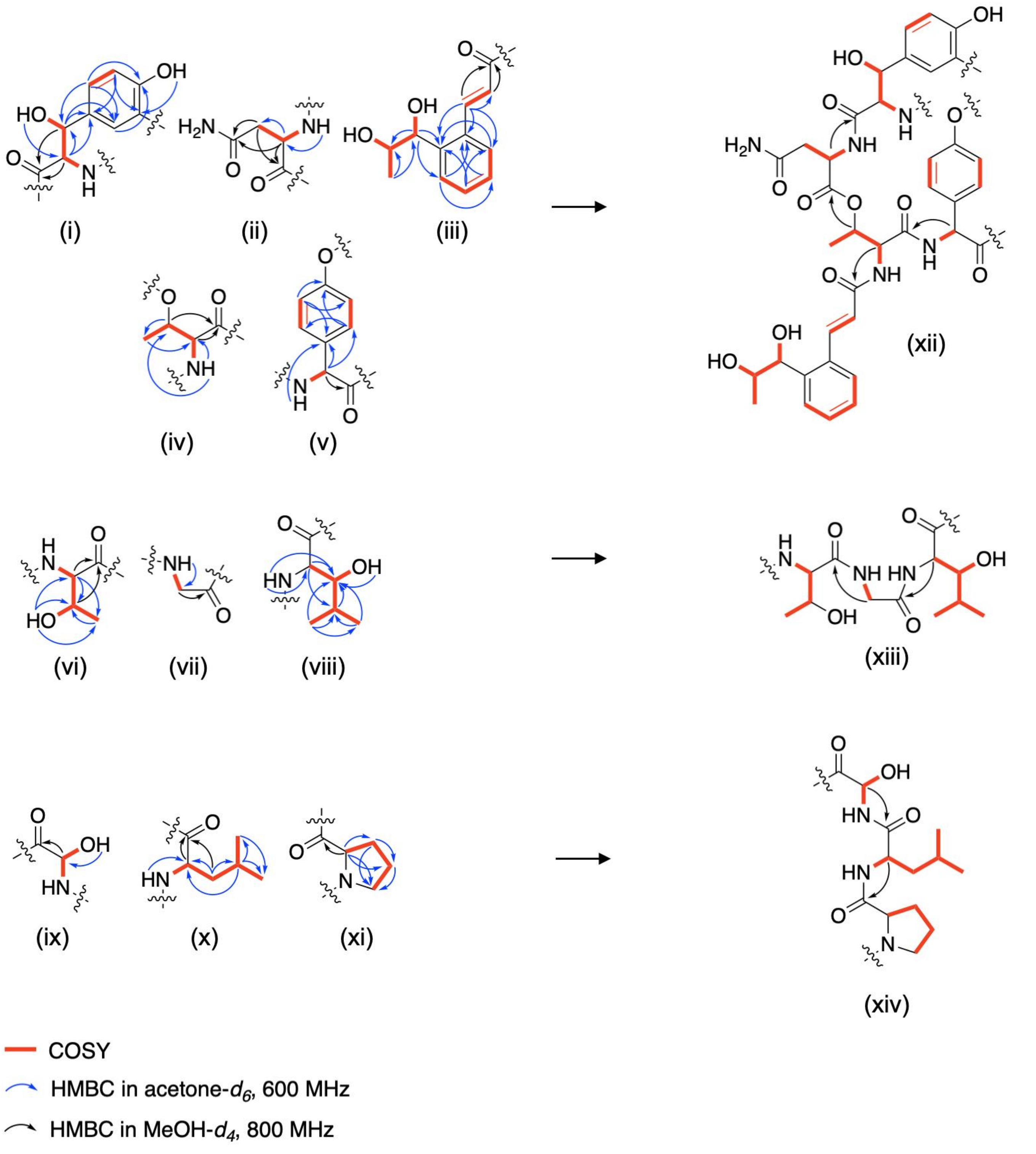

Supplementary Figure 22. Cinnamexin substructures established by NMR spectroscopy. 11 partial structures i-xi were first established from NMR data collected in acetone-d_6. NMR spectra collected in methanol-d_4 enabled substructures i-xi to be connected to generate the three new substructures xii, xiii and xiv.

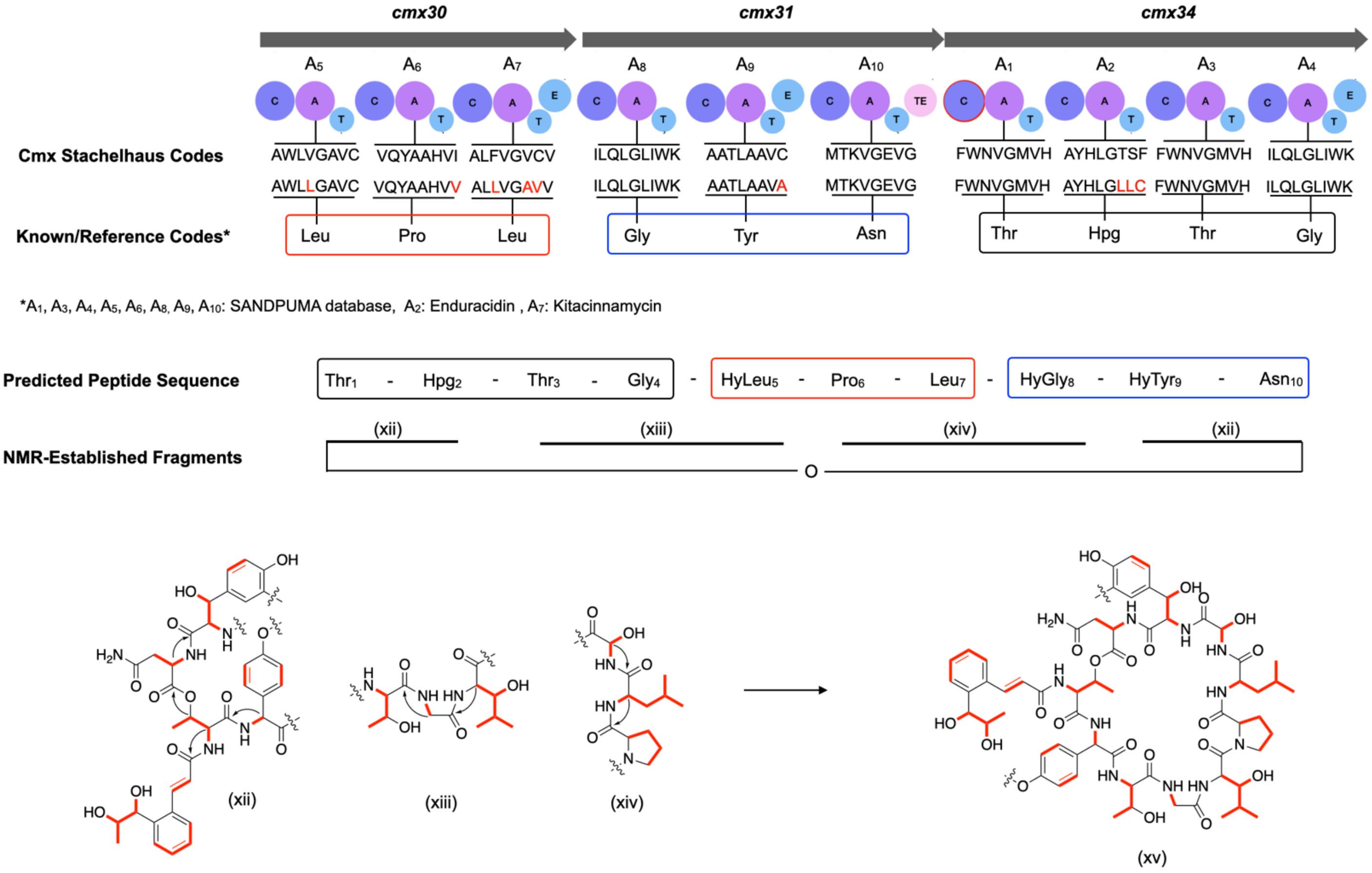

Supplementary Figure 23. Adenylation (A) domain analysis of the *cmx* gene cluster establishes the connectivity of cinnamexin peptide backbone from substructures (xii, xiii & xiv).

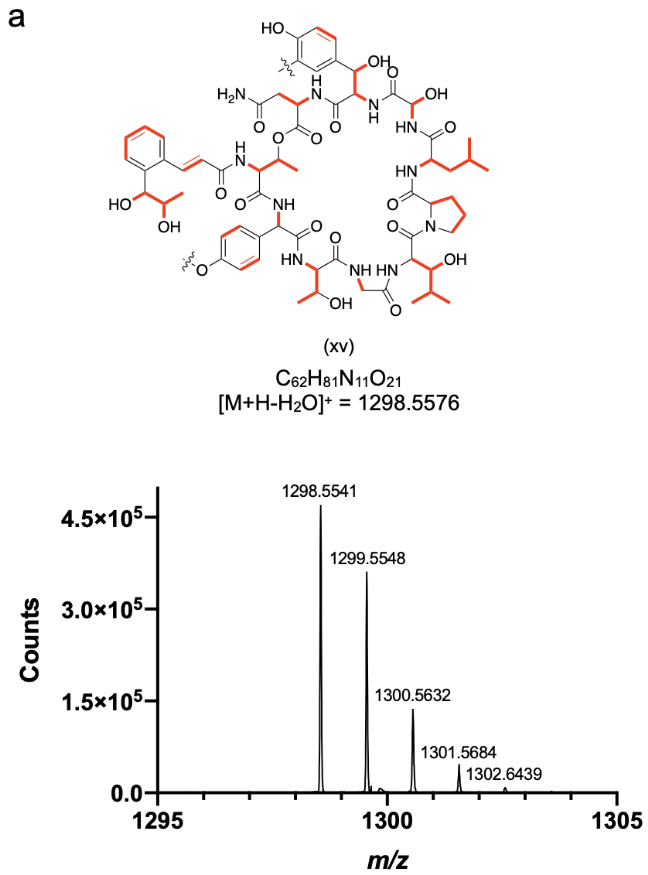

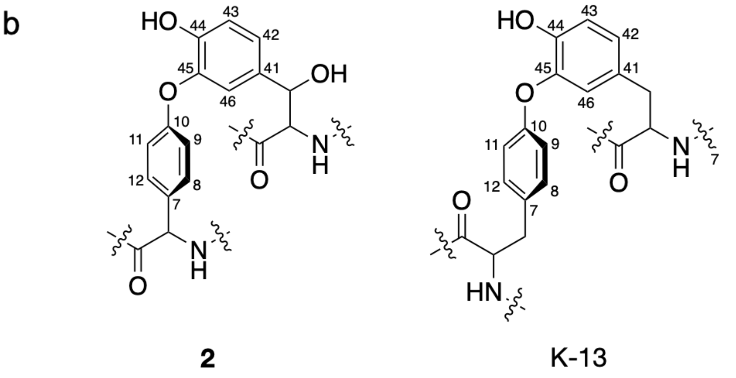

Residue	Position	δ_C in ppm	
		**2**	**K-13**
HyTyr	41	133.3	131.5
	42	122.3	125.6
	43	117.7	117.3
	44	149.3	147.1
	45	149.6	148.0
	46	121.1	119.4
Hpg	7	133.7	133.0
	8	133.3	132.1
	9	124.1	121.3
	10	162.2	158.2
	11	125.8	122.5
	12	130.6	131.2

Both in CD_3OD.

Supplementary Figure 24. Determination of the presence of a crosslink between between HyTyr and Hpg in **2**. (a) Comparison of substructure xv to the high-resolution mass spectrum of **2** indicates that additional atoms (e.g. a hydroxyl group at C-45) cannot be added while still satisfying the deduced molecular formula. (b) ^{13}C NMR chemical shifts of the Hpg and HyTyr side chains in **2** are consistent with the peptide natural product K-13, which bears a similar biaryl motif to that proposed in **2.**

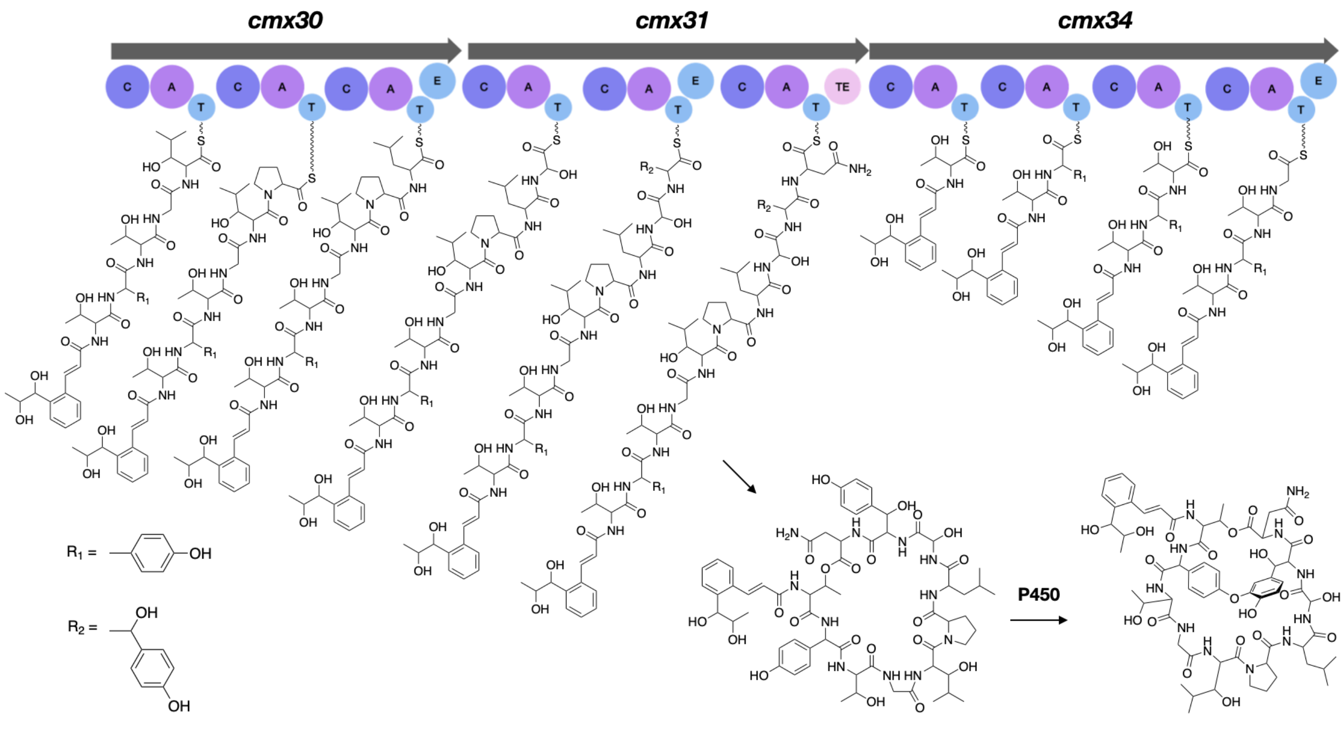

2

Supplementary Figure 25. Proposed NRPS assembly line biosynthesis of cinnamexin (**2**). The first and last modules in the NRPS assembly line were assigned based on the presence of a C_{START} domain in Cmx34 and thioesterase domain in Cmx31. The crosslink between HyTyr and Hpg is attributed to one of the P450s encoded by the BGC.

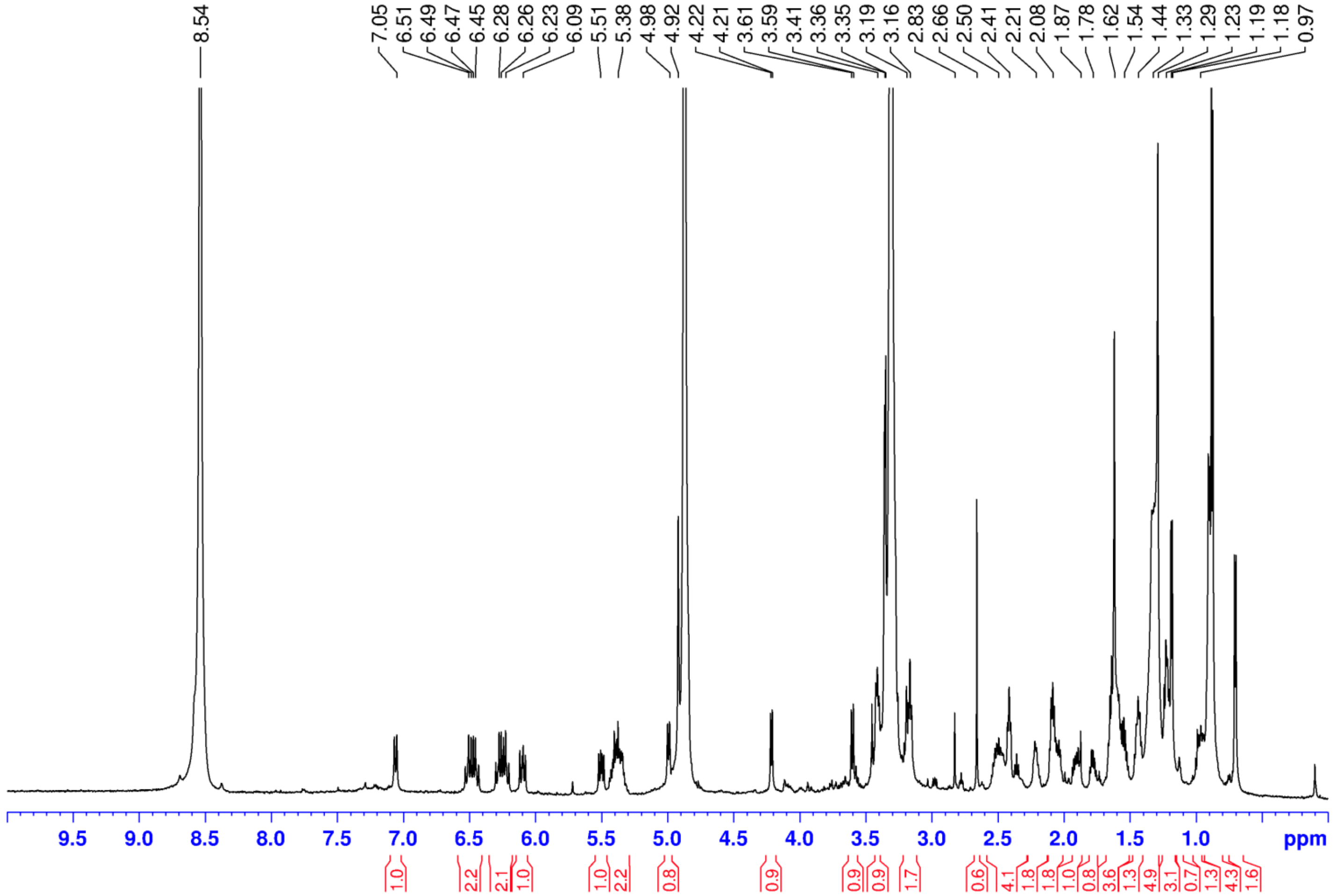

Supplementary Figure 26. ^{1}H NMR spectrum of **3** in CD_3OD. Conkatamycin signals were acquired at 600 MHz.

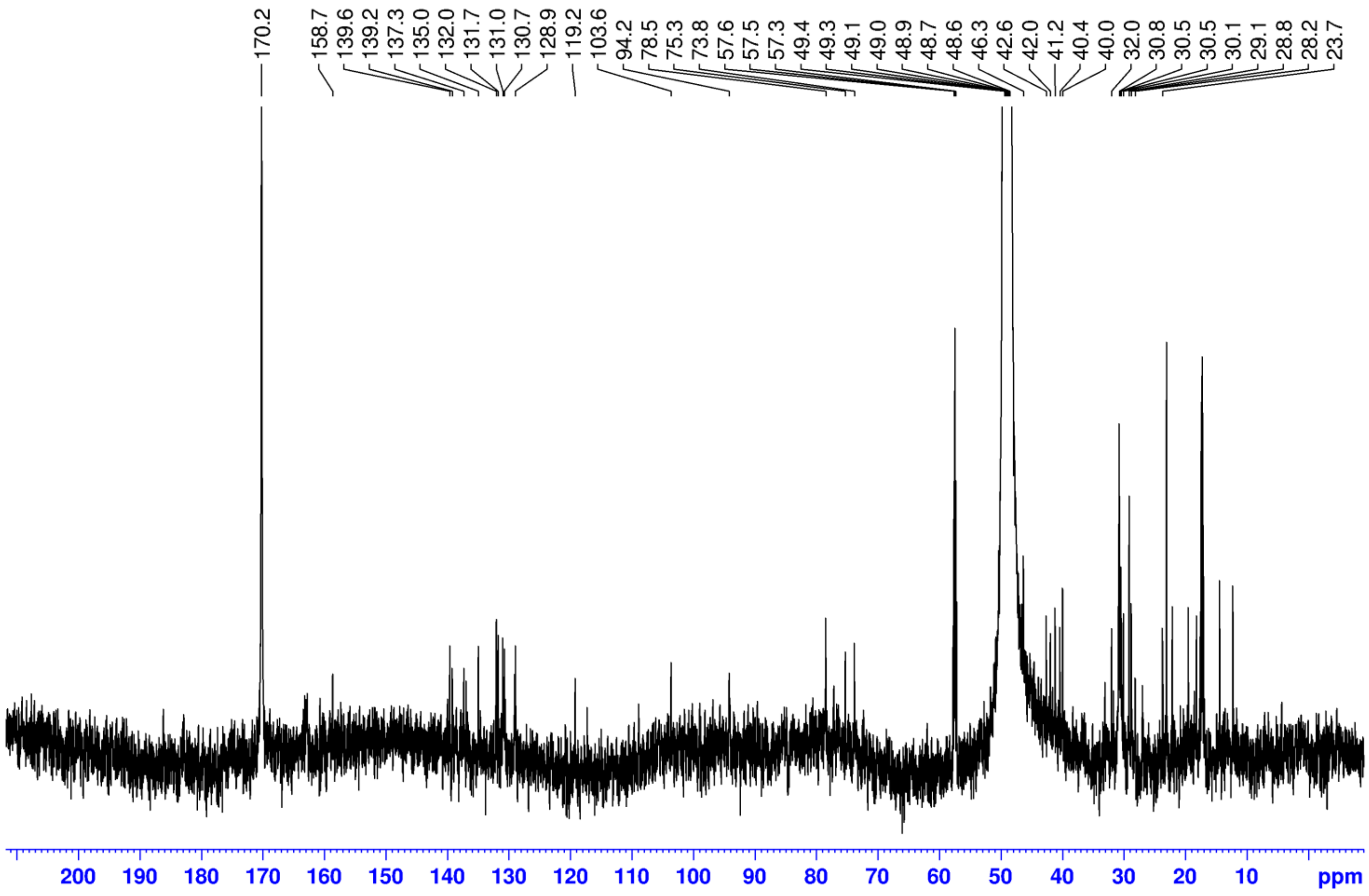

Supplementary Figure 27. ^{13}C NMR spectrum of **3** in CD_3OD. Conkatamycin signals were acquired at 151 MHz.

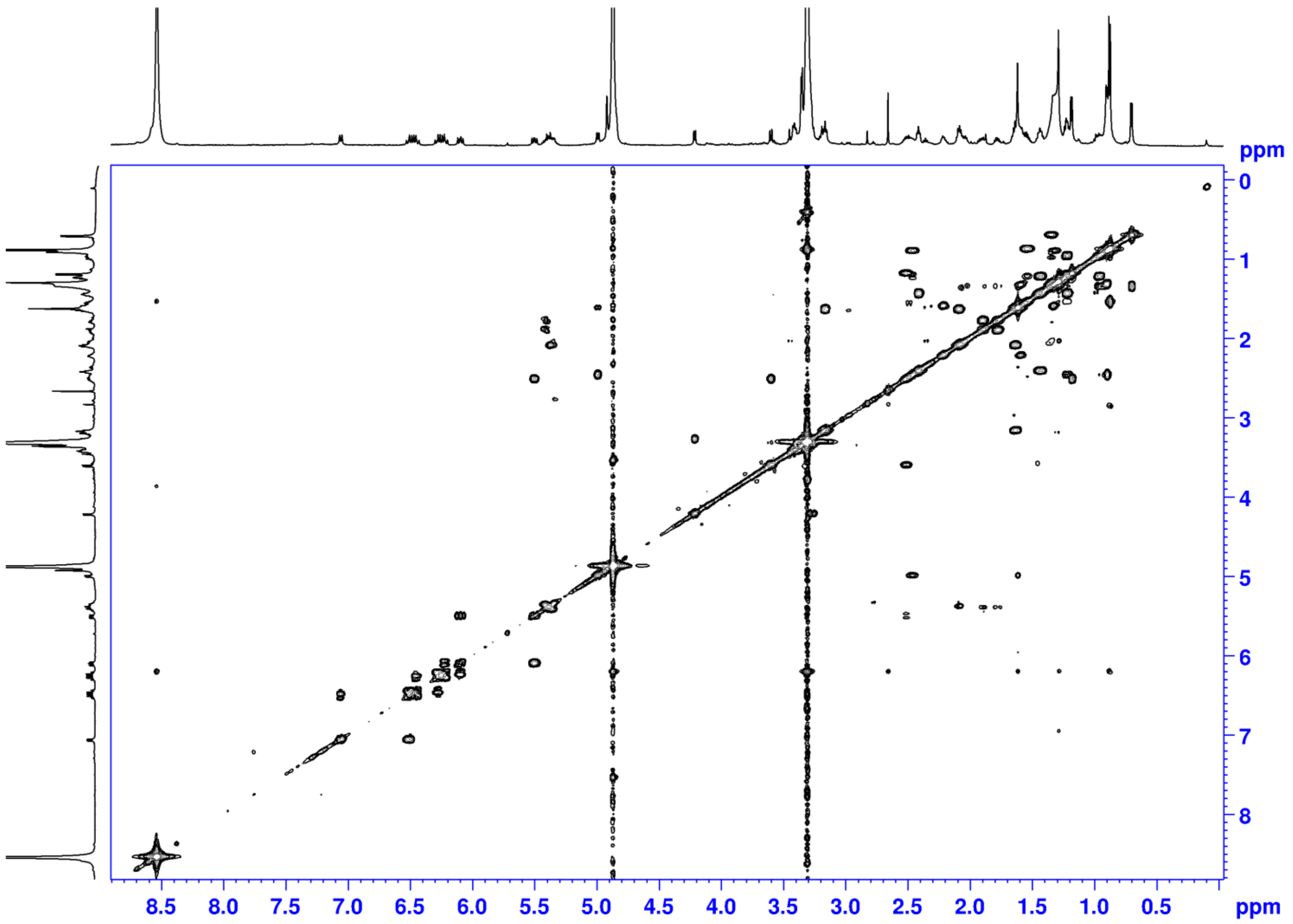

Supplementary Figure 28. COSY NMR spectrum of **3** in CD_3OD. Conkatamycin signals were acquired at 600 MHz.

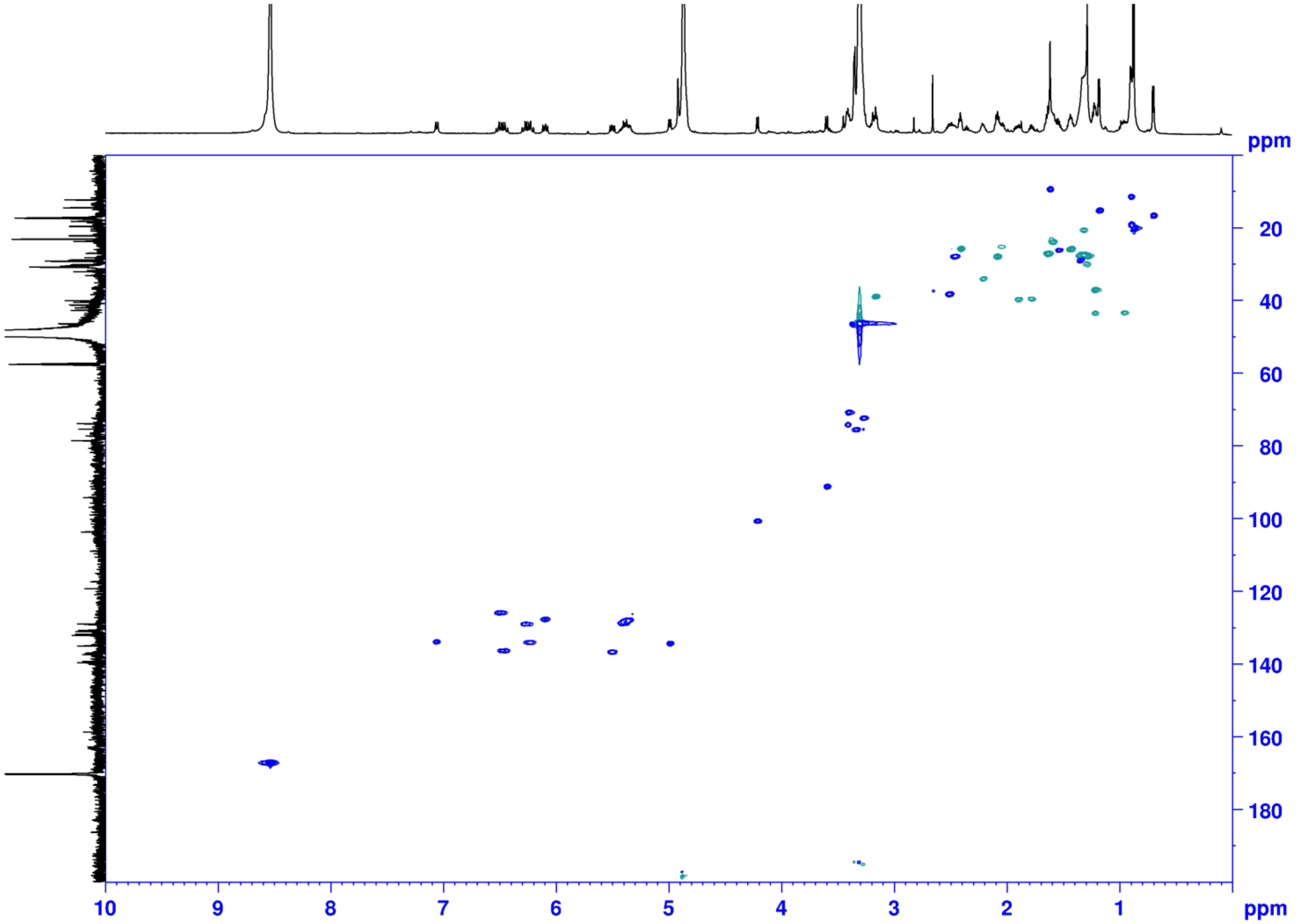

Supplementary Figure 29. Multiplicity-edited HSQC NMR spectrum of **3** in CD_3OD (^1H: 600MHz, ^{13}C:151 MHz). Blue contours correspond to methyls and methines, and teal contours correspond to methylenes.

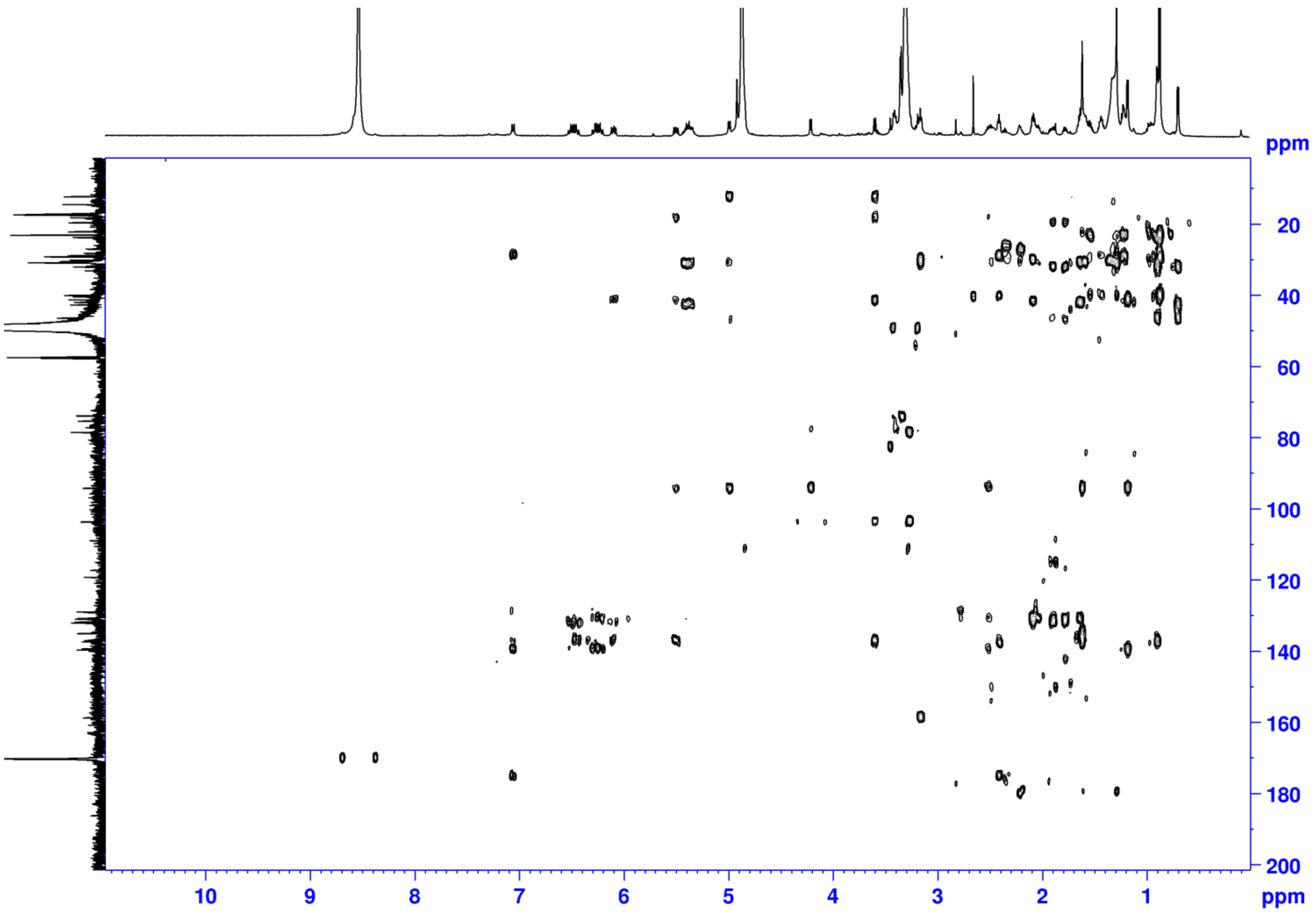

Supplementary Figure 30. HMBC NMR spectrum of **3** in CD_3OD. ^{1}H signals were acquired at 600MHz. ^{13}C signals were acquired at 151 MHz.

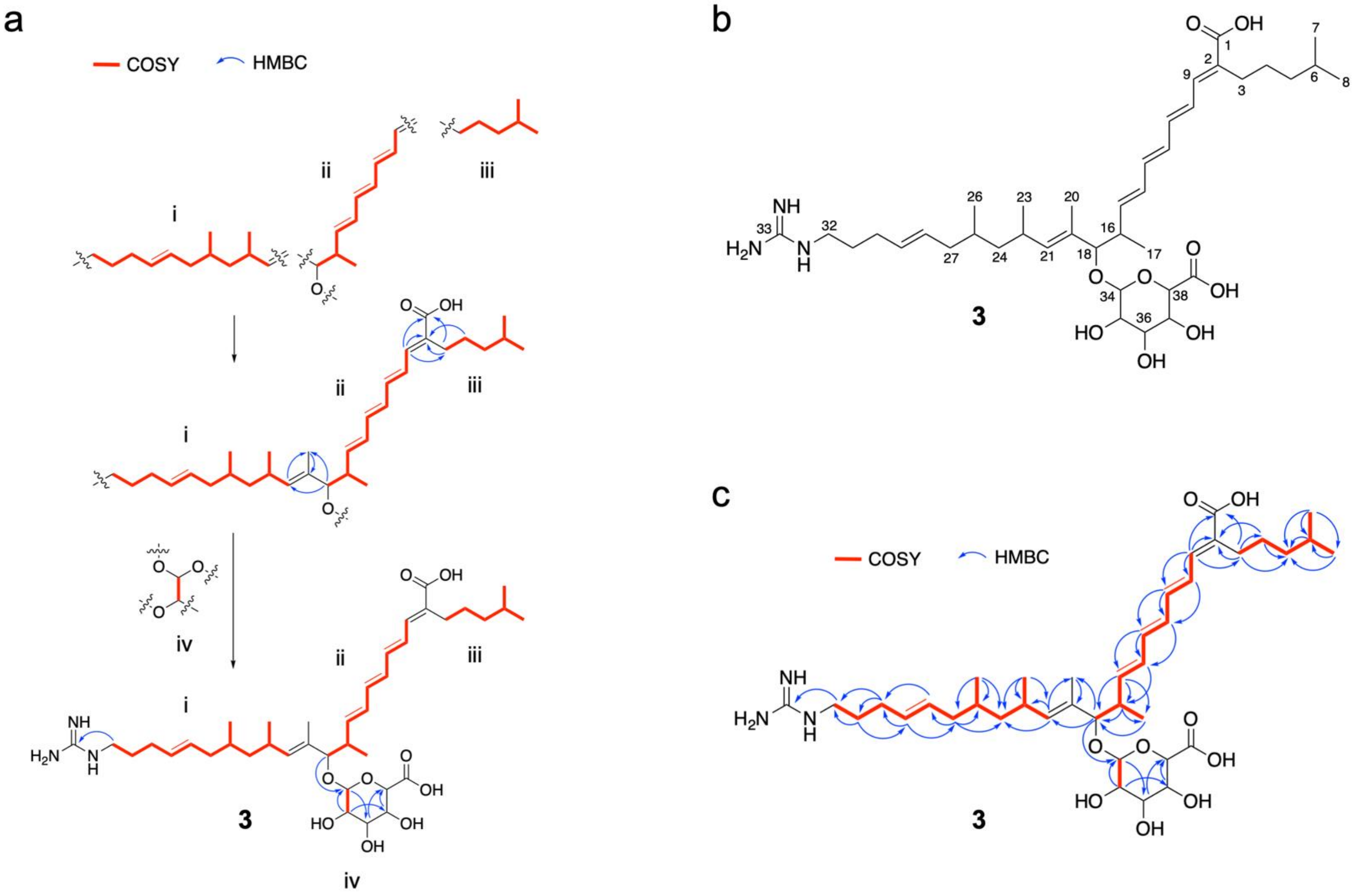

Supplementary Figure 31. NMR spectroscopic characterization of **3.** (a) The four observed ^{1}H-^{1}H spin systems observed in **3** with key HMBC correlations that establish their connectivity to one another and additional atoms. (b) The numbering scheme for **3**. (c) Compound **3** annotated with all COSY correlations and all non-redundant HMBC correlations.

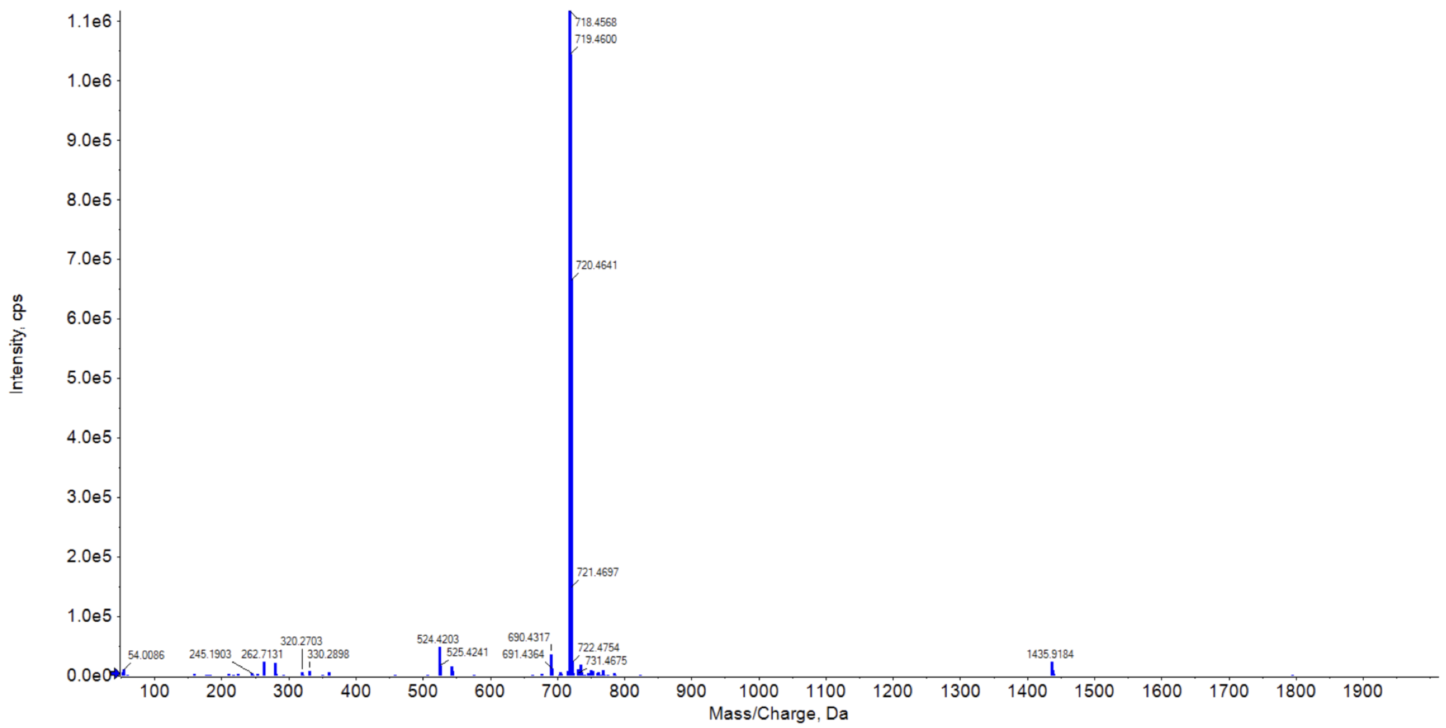

Supplementary Figure 32. ESI+ high-resolution mass spectrum of conkatamycin (**3**). Conkatamycin was determined to have a molecular formula of $C_{39}H_{63}N_3O_9$ based on *m/z* 718.4568, which represents its protonated adduct (calculated mass for $C_{39}H_{64}N_3O_9^+$ = 718.4637, Δ = 9.6 ppm). The fragment at *m/z* 524.4203 corresponds to the protonated aglycone of **3**, which is consistent with the loss of a substituent with the same mass as glucuronic acid.

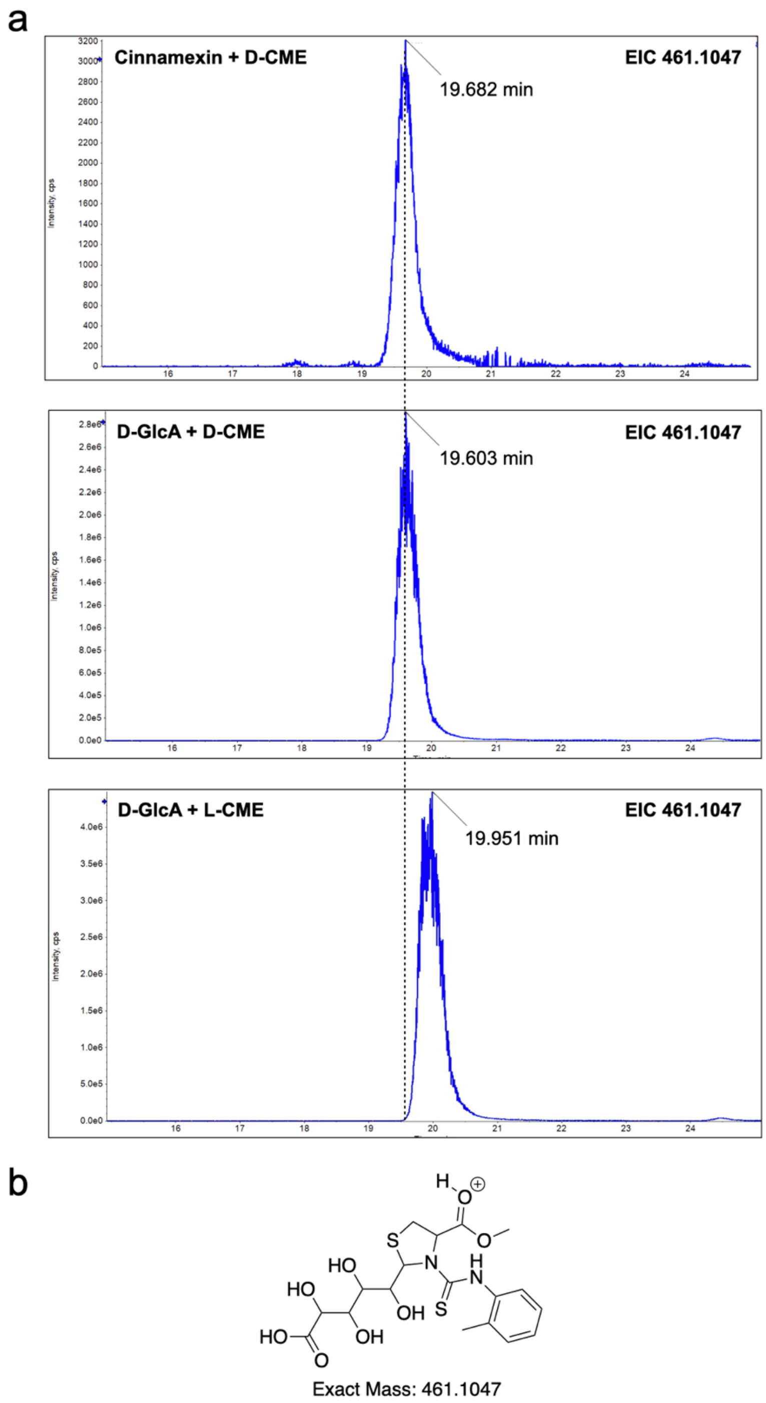

Supplementary Figure 33. Determination of the sugar constitution in conkatamycin (**3**) using Tanaka's method. (a) LC-HRMS comparison of hydrolyzed compound **3** to D-GlcA using Tanaka's method. (b) Structure of the protonated adduct of GlcA derivatized using Tanaka's method.

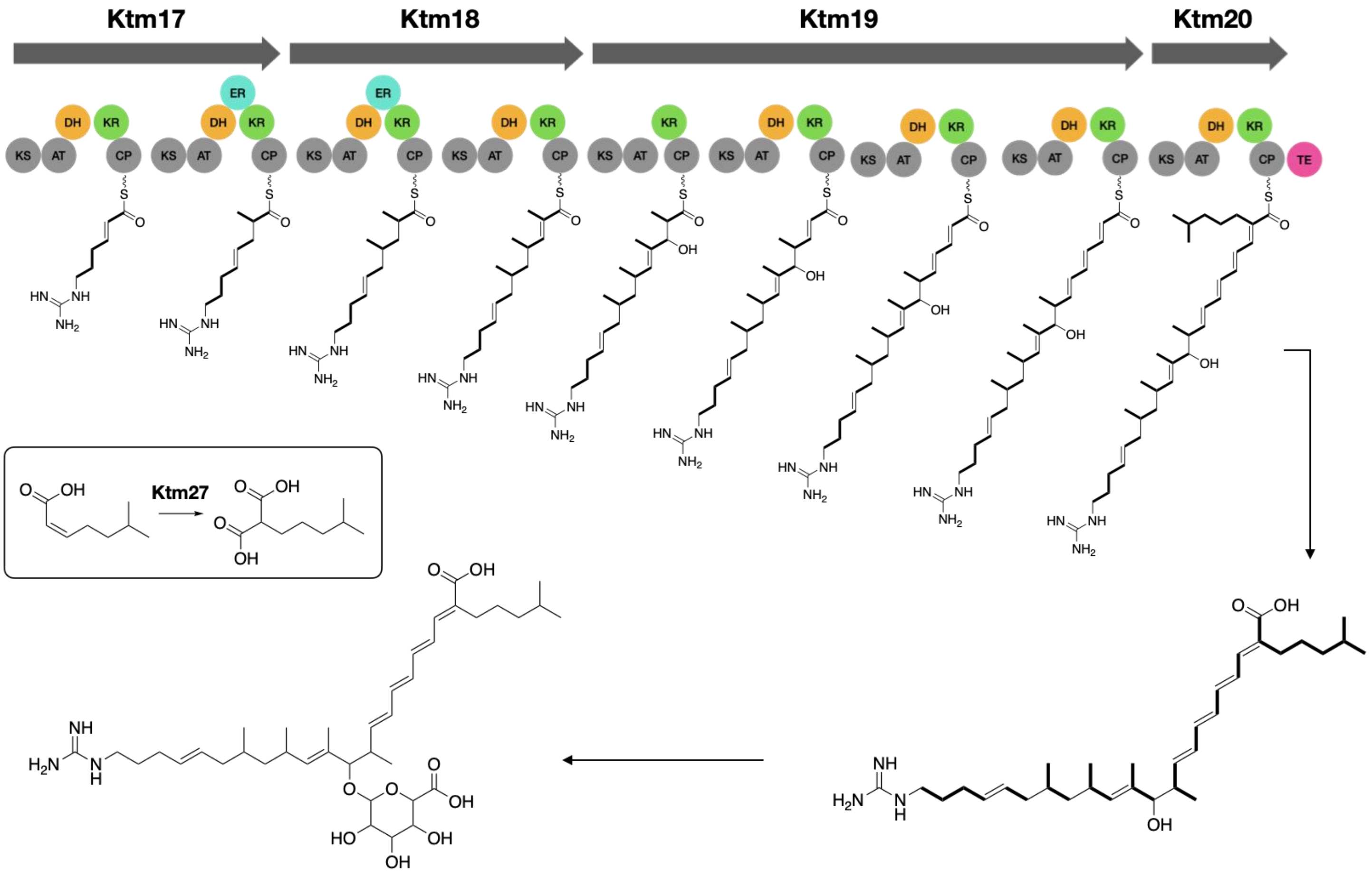

Supplementary Figure 34. Proposed PKS assembly line biosynthesis of conkatamycin (**3**). Ktm27 (predicted as a crotonyl-CoA_carboxylase/reductase) is proposed to be involved in the biosynthesis of the rare PKS building block (isohexylmalonyl-CoA).

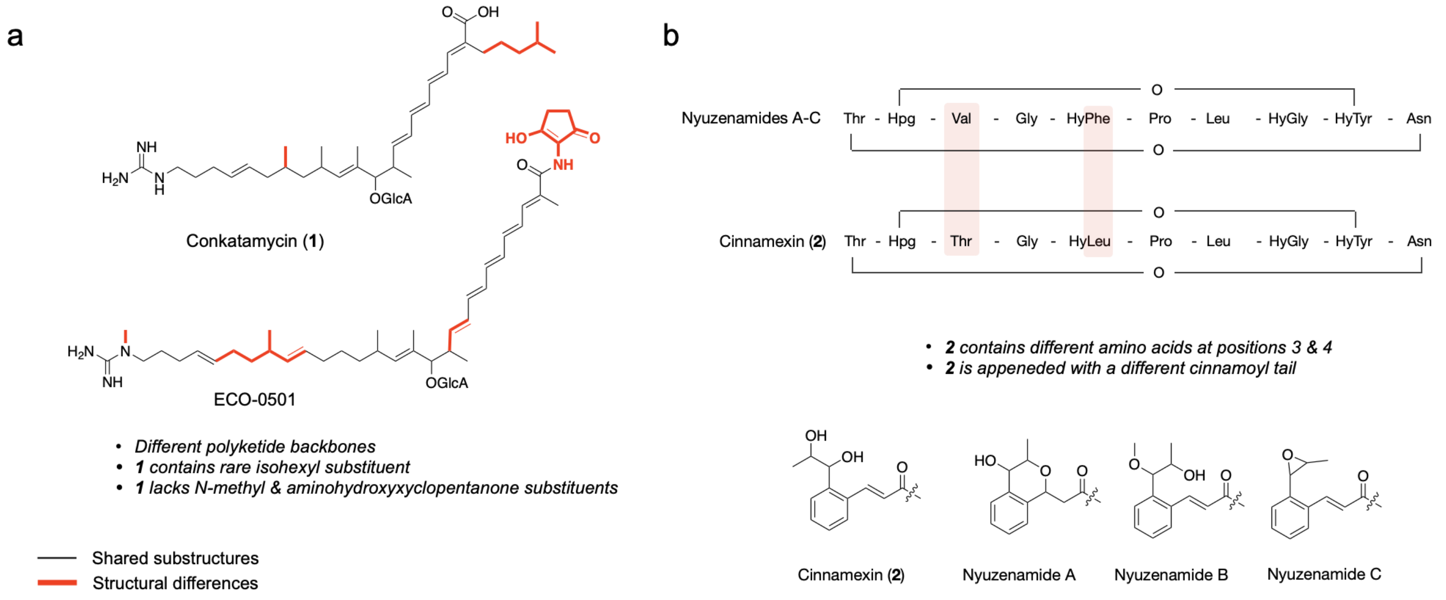

Supplementary Figure 35. Structural comparison of (a) conkatamycin (**3**) and (b) cinnamexin (**2**) to their most structurally related natural products resulting from a SciFinder Scholar search. No naturally occurring relative of prolinolexin (**1**) was found.

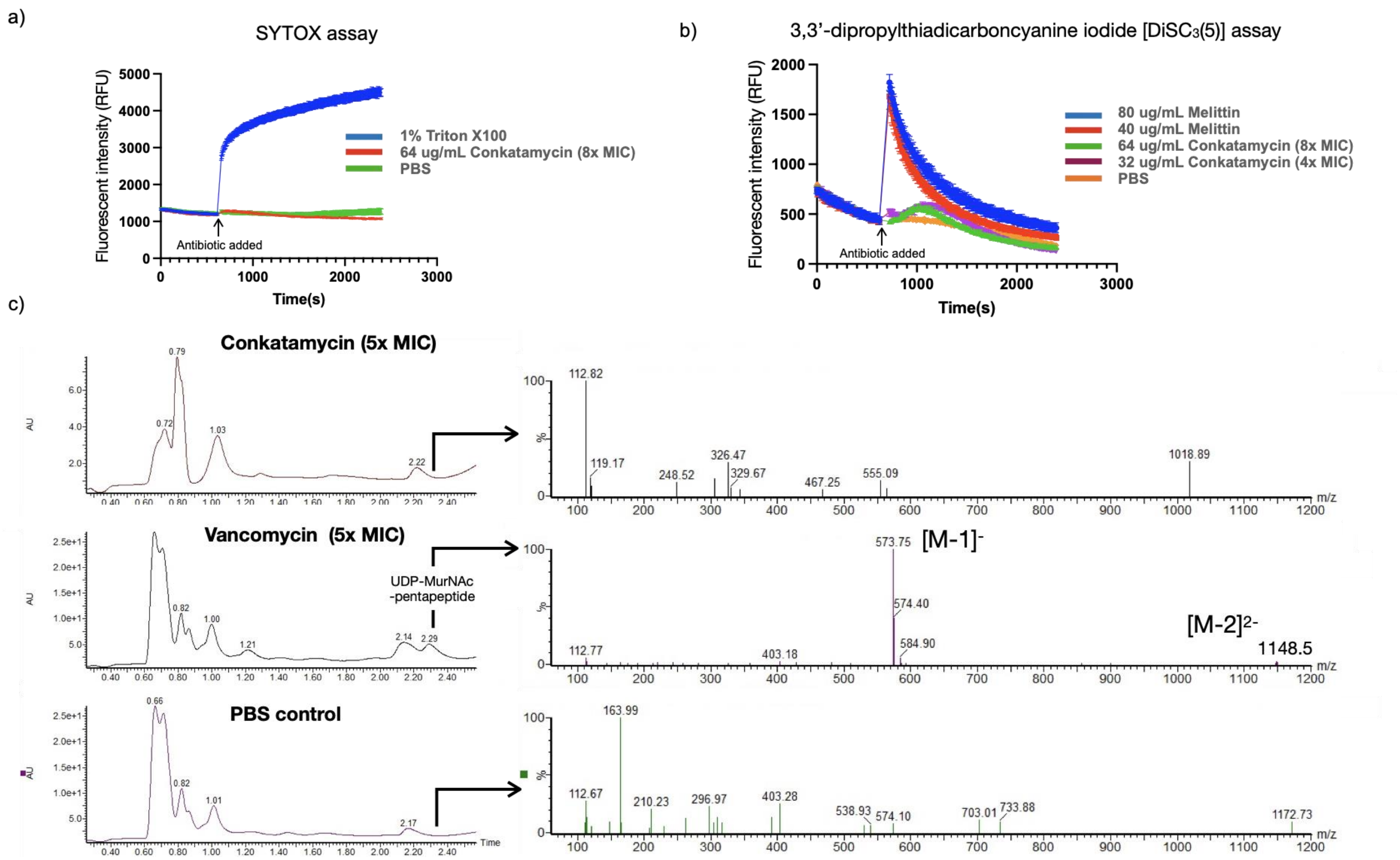

Supplementary Figure 36. Conkatamycin MOA analysis. Potent antibacterial activity, with no detectable resistance, can point to a detergent-like activity for an antibiotic. The possibility that conkatamycin might disrupt the bacterial membrane was therefore explored in more detail. No response was detected in either **a**) SYTOX based fluorescence assay for cell lytic activity and **b**) 3,3'-dipropylthiadicarboncyanine iodide [DiSC$_3$(5)] based fluorescence assay for membrane depolarization activity even when *S. aureus* was exposed to high concentrations (4x and 8x MIC) of conkatamycin. **c**) Another common MOA for antibiotics that fail to easily develop resistance in the laboratory is inhibition of cell wall biosynthesis. Disruption of cell wall biosynthesis often leads to an accumulation the lipid II precursor UDP-MurNAc-pentapeptide in antibiotic treated cultures[10–12]. LCMS analysis of UDP-MurNAc-pentapeptide accumulation in antibiotic treated *S. aureus* cultures revealed that unlike cells treated with the vancomycin control, conkatamycin treated cells (1x or 4x MIC) did not show elevated UDP-MurNAc-pentapeptide levels when compared to untreated cells.

Supplementary references

1. Libis, V. *et al.* Uncovering the biosynthetic potential of rare metagenomic DNA using co-occurrence network analysis of targeted sequences. *Nat. Commun.* **10**, 3848 (2019).
2. Yamanaka, K. *et al.* Direct cloning and refactoring of a silent lipopeptide biosynthetic gene cluster yields the antibiotic taromycin A. *Proc. Natl. Acad. Sci.* **111**, 1957–1962 (2014).
3. Fu, J. *et al.* Full-length RecE enhances linear-linear homologous recombination and facilitates direct cloning for bioprospecting. *Nat. Biotechnol.* **30**, 440–446 (2012).
4. Wang, H. *et al.* ExoCET: exonuclease *in vitro* assembly combined with RecET recombination for highly efficient direct DNA cloning from complex genomes. *Nucleic Acids Res.* **46**, e28–e28 (2018).
5. Jiang, W. *et al.* Cas9-assisted targeting of chromosome segments CATCH enables one-step targeted cloning of large gene clusters. *Nat. Commun.* **6**, 8101 (2015).
6. Enghiad, B. *et al.* Cas12a-assisted precise targeted cloning using *in vivo* Cre-lox recombination. *Nat. Commun.* **12**, 1171 (2021).
7. Liang, M. *et al.* Activating cryptic biosynthetic gene cluster through a CRISPR-Cas12a-mediated direct cloning approach. *Nucleic Acids Res.* **50**, 3581-3592 (2022).
8. Kepplinger, B. *et al.* Mode of action and heterologous expression of the natural product antibiotic vancoresmycin. *ACS Chem. Biol.* **13**, 207–214 (2018).
9. Tocchetti, A., Donadio, S. & Sosio, M. Large inserts for big data: artificial chromosomes in the genomic era. *FEMS Microbiol. Lett.* **365**, 64 (2018).
10. Hover, B. M. *et al.* Culture-independent discovery of the malacidins as calcium-dependent antibiotics with activity against multidrug-resistant Gram-positive pathogens. *Nat. Microbiol.* **3**, 415–422 (2018).
11. Ling, L. L. *et al.* A new antibiotic kills pathogens without detectable resistance. *Nature* **517**, 455–459 (2015).
12. Schneider, T. *et al.* The lipopeptide antibiotic Friulimicin B inhibits cell wall biosynthesis through complex formation with bactoprenol phosphate. *Antimicrob. Agents Chemother.* **53**, 1610-1618 (2009).
