# Supplementary material for: Multiplexed mobilization and expression of biosynthetic gene clusters
Source: Nat Commun. 2022 Sep 6;13:5256. doi: 10.1038/s41467-022-32858-0 (PMC9448795; doi:10.1038/s41467-022-32858-0)
Supplement: Supplementary file 2 — Reporting Summary [file 41467_2022_32858_MOESM2_ESM.pdf]

nature research

Corresponding author(s): Sean F. Brady
Last updated by author(s): Aug 16, 2022

Reporting Summary

Nature Research wishes to improve the reproducibility of the work that we publish. This form provides structure for consistency and transparency in reporting. For further information on Nature Research policies, see Authors & Referees and the Editorial Policy Checklist .

Statistics

For all statistical analyses, confirm that the following items are present in the figure legend, table legend, main text, or Methods section.

n/a	Confirmed	
☒	☐	The exact sample size (*n*) for each experimental group/condition, given as a discrete number and unit of measurement
☒	☐	A statement on whether measurements were taken from distinct samples or whether the same sample was measured repeatedly
☒	☐	The statistical test(s) used AND whether they are one- or two-sided<br>*Only common tests should be described solely by name; describe more complex techniques in the Methods section.*
☒	☐	A description of all covariates tested
☒	☐	A description of any assumptions or corrections, such as tests of normality and adjustment for multiple comparisons
☒	☐	A full description of the statistical parameters including central tendency (e.g. means) or other basic estimates (e.g. regression coefficient) AND variation (e.g. standard deviation) or associated estimates of uncertainty (e.g. confidence intervals)
☒	☐	For null hypothesis testing, the test statistic (e.g. *F*, *t*, *r*) with confidence intervals, effect sizes, degrees of freedom and *P* value noted<br>*Give P values as exact values whenever suitable.*
☒	☐	For Bayesian analysis, information on the choice of priors and Markov chain Monte Carlo settings
☒	☐	For hierarchical and complex designs, identification of the appropriate level for tests and full reporting of outcomes
☒	☐	Estimates of effect sizes (e.g. Cohen's *d*, Pearson's *r*), indicating how they were calculated

Our web collection on statistics for biologists contains articles on many of the points above.

Software and code

Policy information about availability of computer code

Data collection	N/A
Data analysis	Amplicon sequences were demultiplexed using a Python script available at https://github.com/brady-lab-rockefeller/paired-end-debarcoder. Generation of networks of biosynthetic domains and tracking of their physical location in the PAC library was done with a python script available at https://github.com/VincentLibis/multiplexed_BGC_recovery/blob/main/notebooks/CONKAT-seq_PAC_localization-v1.0.ipynb. Analysis was performed using Python 2.7.15 (Anaconda, Inc.) with the following module dependencies: numpy (1.14.3), pandas (0.23.0), networkx (2.1), BioPython (1.68), scipy (1.1.0) and statsmodels (0.9.0). Biosynthetic domains were compared to reference sequences using blastp (version 2.6.0+). Additional softwares used in the analysis are VSEARCH16 (version 2.9.1), minimap2 (version 2.17), SAMtools (version 1.9), antiSMASH (version 6.0), Flye (version 2.7.1). Visualization of networks was done with Cytoscape (version 3.8.2). Analysis of mass spectrometry data was done with MZmine2 (version 2.53).

For manuscripts utilizing custom algorithms or software that are central to the research but not yet described in published literature, software must be made available to editors/reviewers. We strongly encourage code deposition in a community repository (e.g. GitHub). See the Nature Research guidelines for submitting code & software for further information.

Data

Policy information about availability of data

All manuscripts must include a data availability statement. This statement should provide the following information, where applicable:
- Accession codes, unique identifiers, or web links for publicly available datasets
- A list of figures that have associated raw data
- A description of any restrictions on data availability

Sequence data that support the findings of this study have been deposited in GenBank with the accession numbers OL452059 to OL452062. The 15 whole genomes used as reference in the study are available on GenBank with the accession numbers NZ_CP012382.1, NZ_CP086102.1, NZ_BMSY00000000.1, NZ_BMWF00000000.1, NZ_BMVE00000000.1, NZ_BNBZ00000000.1, NZ_BLIR00000000.1, NZ_BMSC00000000.1, NZ_BMTO00000000.1, NZ_BNBT00000000.1,

NZ_KQ948550.1, NZ_BMSE00000000.1, NZ_BMSO00000000.1, NZ_BMSZ00000000.1, NZ_BMUW00000000.1. Source data are provided with this paper

Field-specific reporting

Please select the one below that is the best fit for your research. If you are not sure, read the appropriate sections before making your selection.

- [x] Life sciences
- [ ] Behavioural & social sciences
- [ ] Ecological, evolutionary & environmental sciences

For a reference copy of the document with all sections, see nature.com/documents/nr-reporting-summary-flat.pdf

Life sciences study design

All studies must disclose on these points even when the disclosure is negative.

Sample size	We selected sample size based on our previous experience with equivalent experiments, the published literature, feasibility and the level of variability between replicates. No statistical methods were used to predetermine samples size.
Data exclusions	No data was excluded
Replication	Biological assays: antibacterial n=2, cell wall feeding assay n=2, membrane lysis assay n=3, membrane depolarization assay n=3. All attempts at replication were successful
Randomization	None of the experiments involved allocation of samples to test groups
Blinding	None of the experiments involved allocation of samples to test groups

Reporting for specific materials, systems and methods

We require information from authors about some types of materials, experimental systems and methods used in many studies. Here, indicate whether each material, system or method listed is relevant to your study. If you are not sure if a list item applies to your research, read the appropriate section before selecting a response.

Materials & experimental systems

n/a	Involved in the study	
x		Antibodies
x		Eukaryotic cell lines
x		Palaeontology
x		Animals and other organisms
x		Human research participants
x		Clinical data

Methods

n/a	Involved in the study	
x		ChIP-seq
x		Flow cytometry
x		MRI-based neuroimaging
